# VaDiR: an integrated approach to Variant Detection in RNA

**DOI:** 10.1093/gigascience/gix122

**Published:** 2017-12-18

**Authors:** Lisa Neums, Seiji Suenaga, Peter Beyerlein, Sara Anders, Devin Koestler, Andrea Mariani, Jeremy Chien

**Affiliations:** 1 Department of Cancer Biology, University of Kansas Medical Center, 3901 Rainbow Blvd., Kansas City, KS 66160, USA; 2 Department of Bioinformatics and Biosystems Technology, University of Applied Sciences Wildau, Hochschulring 1, 15745 Wildau, Germany; 3 Obstetrics and Gynecology, Cancer Center, Mayo Clinic, 200 First St. SW, Rochester, MN 55905, USA; 4 Department of Biostatistics, University of Kansas Medical Center, 3901 Rainbow Blvd., Kansas City, KS 66160, USA; 5 Department of Internal Medicine, University of New Mexico Health Sciences Center, 2325 Camino de Salud NE, Albuquerque, NM 87131, USA

**Keywords:** RNA-seq, somatic variant calling, ovarian cancer, cancer genomes, transcriptome

## Abstract

**Background:**

Advances in next-generation DNA sequencing technologies are now enabling detailed characterization of sequence variations in cancer genomes. With whole-genome sequencing, variations in coding and non-coding sequences can be discovered. But the cost associated with it is currently limiting its general use in research. Whole-exome sequencing is used to characterize sequence variations in coding regions, but the cost associated with capture reagents and biases in capture rate limit its full use in research. Additional limitations include uncertainty in assigning the functional significance of the mutations when these mutations are observed in the non-coding region or in genes that are not expressed in cancer tissue.

**Results:**

We investigated the feasibility of uncovering mutations from expressed genes using RNA sequencing datasets with a method called Variant Detection in RNA(VaDiR) that integrates 3 variant callers, namely: SNPiR, RVBoost, and MuTect2. The combination of all 3 methods, which we called Tier 1 variants, produced the highest precision with true positive mutations from RNA-seq that could be validated at the DNA level. We also found that the integration of Tier 1 variants with those called by MuTect2 and SNPiR produced the highest recall with acceptable precision. Finally, we observed a higher rate of mutation discovery in genes that are expressed at higher levels.

**Conclusions:**

Our method, VaDiR, provides a possibility of uncovering mutations from RNA sequencing datasets that could be useful in further functional analysis. In addition, our approach allows orthogonal validation of DNA-based mutation discovery by providing complementary sequence variation analysis from paired RNA/DNA sequencing datasets.

## Background

Next-generation sequencing has enabled the discovery of novel variants in genetic sequences. However, even though the cost of sequencing has decreased in recent years, whole-genome sequencing (WGS) can still be prohibitively expensive in many cases [1]. Sequencing only exonic regions of the genome helps reduce cost, and multiple tools (such as MuTect2, provided by GATK [2], MuSE [3], SomaticSniper [4], and VarScan2 [5]) have been developed for somatic variant discovery using whole-exome sequencing (WES) data, and the performance of these tools was recently evaluated [6]. Still, the reagents used to capture exonic regions are costly and produce uneven coverage across the genome due to capture rate biases [7, 8], and only a fraction of the genes in an exome are actually expressed in any given cell [9]. For diseases like cancer, mutations in expressed regions are of greater interest than in non-exonic or unexpressed exonic regions because they are more likely to affect cellular function directly. The transcriptome is therefore an attractive subject of research in cancer and other human pathologies, and some of the cancer genes, such as FOXL2 in granulosa-cell tumors [10] and ARID1A in clear cell carcinomas of the ovary [11], were initially discovered through transcriptome sequencing.

The calling of variants with sequencing data from the transcriptome (RNA-seq) is more challenging because of the splice junctions. Tools like RVBoost [12], SNPiR [13], or GATK Haplotypecaller are created to address this problem. Somatic variant calling from RNA is more difficult because of RNA processing like RNA editing, allele-specific expression, variable levels of gene expression, and the heterogeneity of tumors, which leads to low variant frequencies of some mutations [14]. Tools such as RVBoost, SNPiR, and GATK Haplotypecaller can be used to perform germline variant calling from RNA, but their performance and limitations for somatic variant calling have not been studied previously. Nonetheless, these approaches have the potential to provide an orthogonal method to validate DNA sequence variations by complementing the analysis with RNA sequence analysis.

Additional challenges include the determination of detected mutations either as germline or somatic. In tumor tissues, somatic mutations differ from the germline variations of a patient that are different from the reference genome. To detect somatic sequence variations, it is necessary to compare DNA sequences from normal tissue, such as blood, with DNA or RNA sequences from tumor tissue. If germline sequence variations are not filtered out, it would be difficult to assign detected variations as either somatic or germline. Additionally, it would be improper to assign a variant discovered in the tumor tissue as a somatic mutation when this particular position has no sufficient coverage in germline sequencing.

It should be noted that the integrated approach used by RADIA [15], which combines the somatic variant sequence analysis from tumor DNA and RNA sequencing, allows the discovery of DNA sequence variations in expressed genes and a better characterization of the effect of mutations on gene expression and phenotypic alterations. However, its use of WES of tumor tissue introduces additional cost. RADIA uses the tumor DNA and normal DNA sequencing datasets in the main analysis, and RNA sequence analysis is used as an orthogonal supplement. DNA sequence variations are considered the ground truth, and RNA variants not supported by DNA sequencing are rejected as false-positives. Although somatic variants discovered only by RNA sequencing have the potential of being false-positives, some of these variants may represent missed calls from tumor DNA sequencing or RNA-editing sites that have not been annotated. A detailed comparison of somatic DNA and RNA variants from different tools will provide us with more precise processing and discovery of sequence variations from RNA and DNA sequencing.

In this study, following the recommendation and practices that are widely adopted in the field of bioinformatics [16, 17], we chose a validated dataset to perform a detailed comparison of somatic DNA and somatic RNA sequence variations from 21 pairs of whole-exome and mRNA sequencing from ovarian cancer genomes. We formulated an approach to utilize 3 publicly available tools, namely MuTect2, RVboost, and SNPiR for variant discovery from RNA sequencing. We evaluated the performance of each tool and established the best combination of these tools that enables discovery of variants from RNA sequence with high precision and recall. We showed that most of the variants that would be classified as false-positives or false-negatives can be explained by biological characteristics. In addition, we investigated the performance of our workflow on artificially spiked variants in coding regions of mRNA sequencing data, and we compared the performance of VaDiR with RADIA. Finally, we showed the performance of our workflow on a biologically relevant study: the comparison of somatic variants in high-grade serous carcinomas collected from patients with chemotherapy-resistant or -sensitive ovarian cancer.

## Data Description

Twenty-one samples of ovarian serous cystadenocarcinoma from The Cancer Genome Atlas (TCGA) were divided into 2 groups: 11 cases who were sensitive to the cancer treatment and 10 cases who were resistant. Sensitive cases had a progression-free survival of more than 18 months, and resistant cases had progression-free survival of less than 12 months. The clinical data for the patients were retrieved from cBioPortal [18–20], and the Illumina sequence files for tumor RNA and normal blood DNA were retrieved from cghub [21] and gdc [22] (Supplementary Table 1). Whole-exome sequencing and mRNA sequencing datasets were available from each patient.

**Table 1:** Performance characteristics of VaDiR with the combination Tier 1

	DNA positive	DNA negative
RNA positive	452	63
RNA negative	1327	

Additional data used for the artificial spiking of variants (see “Detection of artificial spiked variants”) were provided by Dr. Andrea Mariani and came from 3 different tumor samples from a patient with serous ovarian carcinoma.

## Analysis

### Performance characteristics of each method and different combinations of 2 or more methods

**Figure 1:**
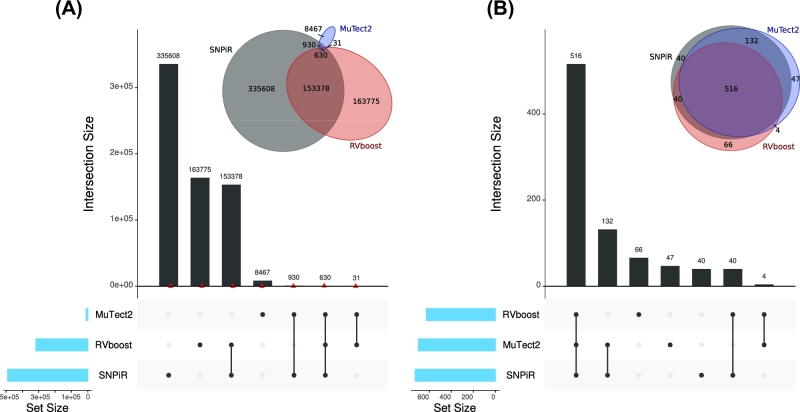
Intersection of the 3 variant calling methods. **(A)** Intersection of the 3 methods with all somatic variants. The red triangles represent the amount of concordant variants. **(B)** Intersection of 3 methods with only concordant somatic variants. All 3 callers (Tier 1) together have the highest number of condordant variants.

To describe the performance characteristics of each method, we use recall and precision metrics instead of sensitivity and specificity because we are interested in variant calls only. Specificity is not a relevant measure because it includes all true-negative calls, which are in the millions. We performed variant calling using RVboost, SNPiR, and MuTect2 separately. Each caller alone calls many variants that are not validated by DNA somatic variants (discordant calls), while SNPiR calls the most variants (Fig. 1A). Mutect2 provides the least amount of variant calls not supported by DNA sequencing compared with the other 2 methods. However, only 10% of variant calls made by Mutect2 were supported by DNA sequencing. These results indicate that any single caller is not adequate in discovering variants with high precision. Therefore, we next tested if any combination of 3 calling methods would provide a higher rate of variant calls supported by DNA sequencing. The combination of all 3 calling methods (hereafter referred to as Tier 1) leads to 81.8% of variants that are validated by DNA somatic variants (concordant calls) with a recall rate of 9% (Fig. 1B, Supplementary Table 2). The combination of Tier 1 with mutations called by Mutect2 and SNPiR (hereafter referred to as Tier 2) leads to a higher recall (11.3%), while the precision is still in a moderate range (41.5%). For the following analysis, we concentrated only on Tier 1.

**Table 2:** Called spiked-in variants

Sample	Tier 1	Tier 2
OV10	68 (54.40%)	78 (62.40%)
OV11	61 (52.59%)	68 (58.62%)
OV12	58 (48.74%)	69 (57.98%)

Percentages represent recall rates in each sample. Tier 1 is the consensus of 3 callers. Tier 2 is the Tier 1 plus consensus of MuTect2 and SNPiR. Total numbers of recoverable spiked-in variants are 125 (OV10), 116 (OV11), and 119 (OV12).

### Effect of weighted features

**Figure 2:**
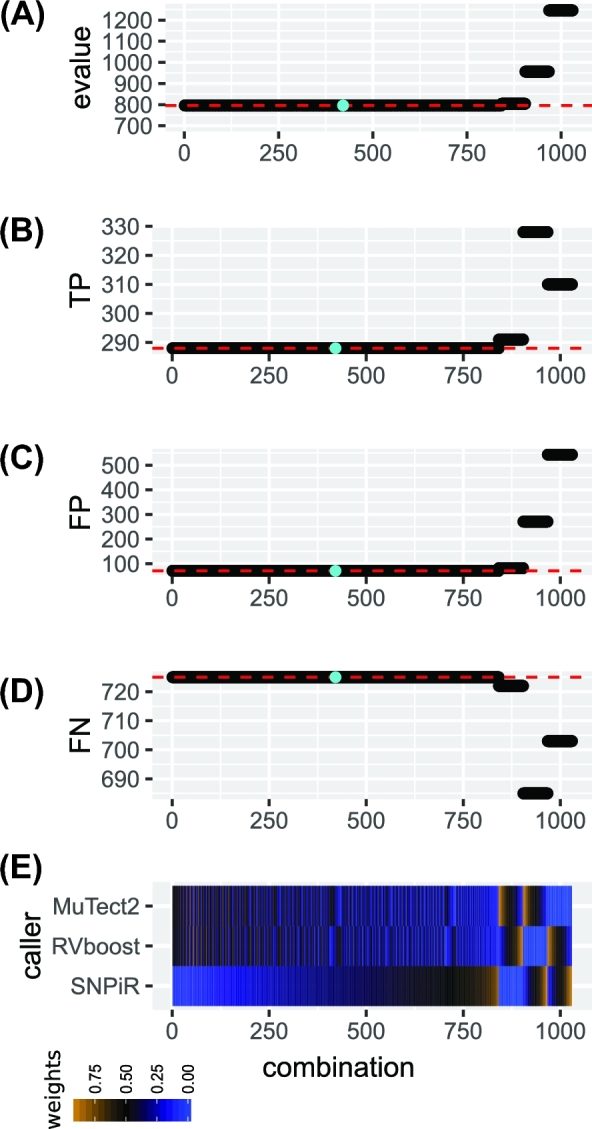
Effect of weighted features on performance. (A–D) The performance of each combination of weights of the 3 callers Haplotypecaller, SNPiR, and RVboost, while evalue means the sum of FP and FN. The blue point marks the equal combination of all 3 callers, namly Tier 1. **(E)** The weights of the callers in each combination.

Additionally, we performed a weighted average of 3 callers with the goal of decreasing the number of false-positive (FP) and false-negative (FN) calls. Specifically, we investigated the effect of different weights on the evalue, which was defined as the sum of FP and FN. The weights on each of the callers were systematically varied from 0 to 1 in increments of 0.1. Evalues were calculated for each weighted combination, and the optimal weights were defined as those that resulted in the smallest evalue. The consensus call of all 3 callers (Tier 1) is denoted in blue (Fig. 2). Our results demonstrate that many different combinations of weights produce similar evalues as compared with the consensus call of all 3 callers (Fig. 2, Supplementary Fig. 1), suggesting that no improvement in performance was gained by weighted average approach. Similarly, no appreciable gain in performance was noted when we considered the variant allele frequency (VAF) in the estimation of the weights (Supplementary Fig. 2). Thus, taken collectively, our results showed little to no benefit in using weighted features.

### Performance of a combined calling method

A total of 634 somatic mutations were called from 21 tumor samples; 516 mutations were concordant and 116 were discordant with mutation calls made from DNA (see Supplementary Table 2). To get a ground truth of variants that could have been called by RNA and were called in tumor DNA, we filtered out all DNA variant calls that had a read depth below 10 in RNA. With this filtering, we found a total of 515 variants that were called at the RNA level, while 452 of them are concordant (true-positive) and 63 discordant (false-positive) (Table 1); 1779 of the 10 361 variants called by DNA callers have read depth greater than 10 at the RNA level, and 1327 of them were missed by RNA calling (74.5% false-negative rate).

#### Variants not found in RNA

**Figure 3:**
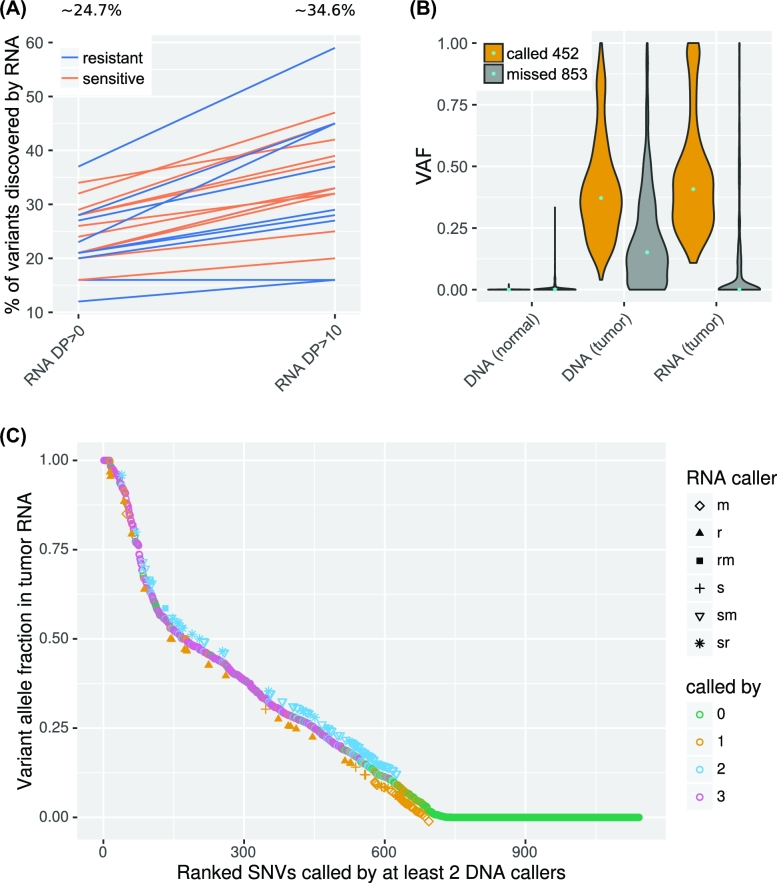
Variants called in tumor DNA. **(A)** Percentage of concordant calls of all somatic variants from expressed genes for each sample from 2 datasets (sensitive and resistant tumor samples). A higher percentage of concordant calls was achieved in transcripts with high expression (DP>10) compared with that of all expressed transcripts (DP>0). **(B)** Violin plot of variant fractions for all somatic variant positions with RNA DP>10. Most of the variant positions missed by VaDiR have a low variant fraction (VAF<0.1) in RNA. **(C)** Ranked SNVs called by TCGA and/or different combinations of RNA-seq calling methods. Only those positions with DP>10 in tumor DNA, RNA, and normal DNA are included in the analysis. The names in the chart are the first letters of the caller SNPiR (s), RVBoost (r), and MuTect (m), or their combinations.

To understand why variant calls from RNA sequencing missed a large majority of variant calls observed by DNA sequencing, we checked the properties of variants missed by RNA callers. From the 10 361 somatic variants called by at least 2 DNA variant callers, 9845 were missed by Tier 1. Out of them 8517 (86.5%) were missed because these variants reside in genes that are not expressed (4628) or expressed less abundantly (3890) (Supplementary Fig. 3). For the mutations in genes with high transcript abundance, 474 (4.8%) were missed because these variants were not in exonic regions. The effect of transcript abundance on variants discovered from RNA-seq could also be observed in the percentage of concordant calls: 516 (24.7%) of the expressed mutations called in DNA in exonic regions were called by Tier 1 (Fig. 3A) but when the expression is higher (DP>10), 34.6% (452 out of 1305 mutations) of the somatic mutations were called. This result confirms that an important factor in RNA-seq variant calling is the expression level.

Among the mutations found by DNA callers but missed by Tier 1 from highly expressed genes (DP>10), 531 (5.4%) of the mutations had a VAF <0.20 in tumor DNA, while 141 of them had a VAF of 0 (Fig. 3B, Supplementary Fig. 4), which can be explained through missed indels and that we accepted only reads with a high quality value in the discovery of the DP of all variants. Additionally, 724 (7.4%) of the missed mutations had a VAF <0.20 in tumor RNA, while 493 of them had a VAF of 0 in tumor RNA. This result confirms that one of the limitations of RNA-based variant calling methods is that they are highly dependent on the VAF. Fig. 3B shows that the VAF of the missed variant is significantly lower than the VAF of called variants both at the DNA and RNA levels (*P* < 0.0001). Moreover, the difference is much greater between the VAF of called variants and missed variants at the RNA level, suggesting that many of the missed variants at the RNA level may be the result of mutations present in a small fraction of tumor cells and the lower expression of mutated transcripts.

**Figure 4:**
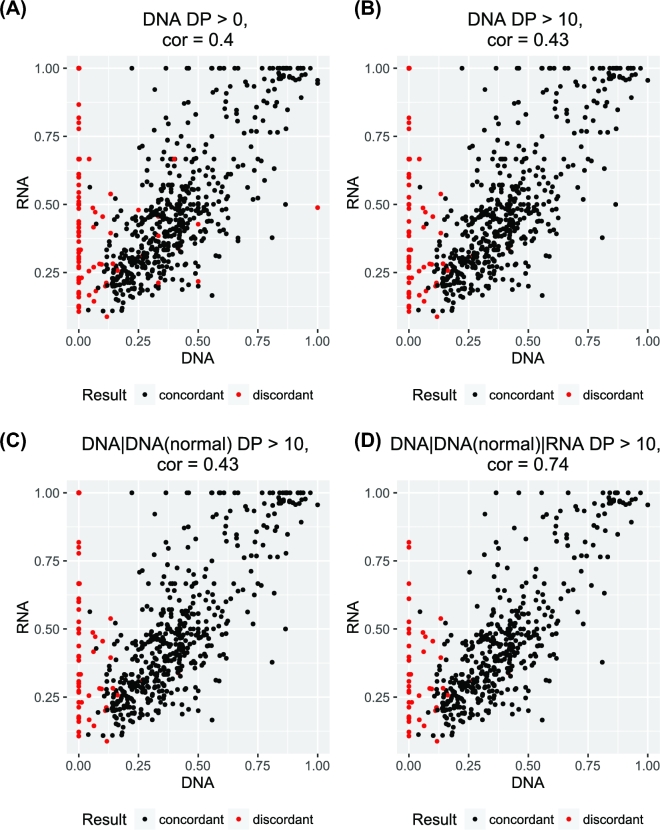
Correlation of variant fractions between RNA and DNA. The 4 charts show the effect of read depth filter on the correlation of variant fractions.

From the variants with high expression and high VAF, 31 mutations were not called by any of the callers. Ninety six mutations were filtered out by at least 1 of the callers because of potential evidence of germline variants or because the realigning step with PBLAT shows that these variants could come from mismapping. Most of the missed variants with low VAF are called by MuTect2 or SNPiR alone or MuTect2 and SNPiR together(Fig. 3C). It is not clear if these missed variants are false-negatives, i.e., true variants missed by VADiR, or if they are false-positives made by DNA callers. Given that many of the missed variant calls (not found by VaDiR) are the result of the PBLAT step in VaDiR to eliminate mis-mapped reads and this step is not used in DNA callers, it is possible that some of the calls missed by VaDiR are true-negatives that are incorrectly called by DNA callers.

#### Variants not found in DNA

The differences in coverage or VAF between DNA and RNA datasets could also contribute to discordant calls. Therefore, we checked those attributes at discordant sites. From all 116 discordant mutations called by Tier 1, 53 (45.7%) had a read depth (DP) of uniquely mapping reads under 10 at the RNA level, and 17 (15.7%) had a read depth under 10 at DNA level (Supplementary Fig. 5). Another 22 (19.0%) mutations had VAF >0 at the DNA level, indicating that these low-level DNA variants were missed by DNA-based callers used by TCGA. Twenty-three variants with a VAF of 0 at the DNA level but high DP in germline DNA, tumor DNA, and tumor RNA were mostly either A>G or C>T (Supplementary Fig. 6). Those variants were found at 12 different positions, of which 1 variant (chr3:58141791 A>G [FLNB:p.M2324V]) is found in 4 different samples and another (chr20:10285837 C>T) in 9 different samples. These likely represent unannotated RNA-editing sites [23–25].

**Figure 5:**
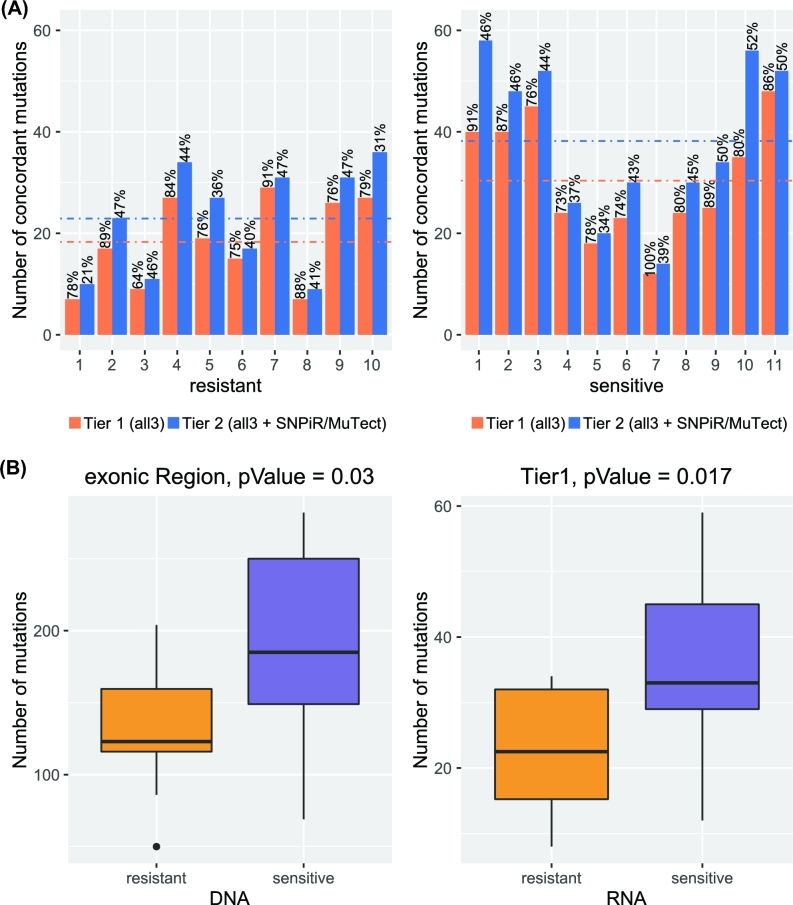
Comparison of sensitive and resistant samples. **(A)** Numbers of concordant calls in Tier 1 and Tier 2 by VaDiR. The precision for each sample for Tier 1 and Tier 2 is shown in percentage above each bar. **(B)** Numbers of mutations found at the DNA and RNA levels in sensitive tumors are significantly higher than in resistant tumor samples.

**Figure 6:**
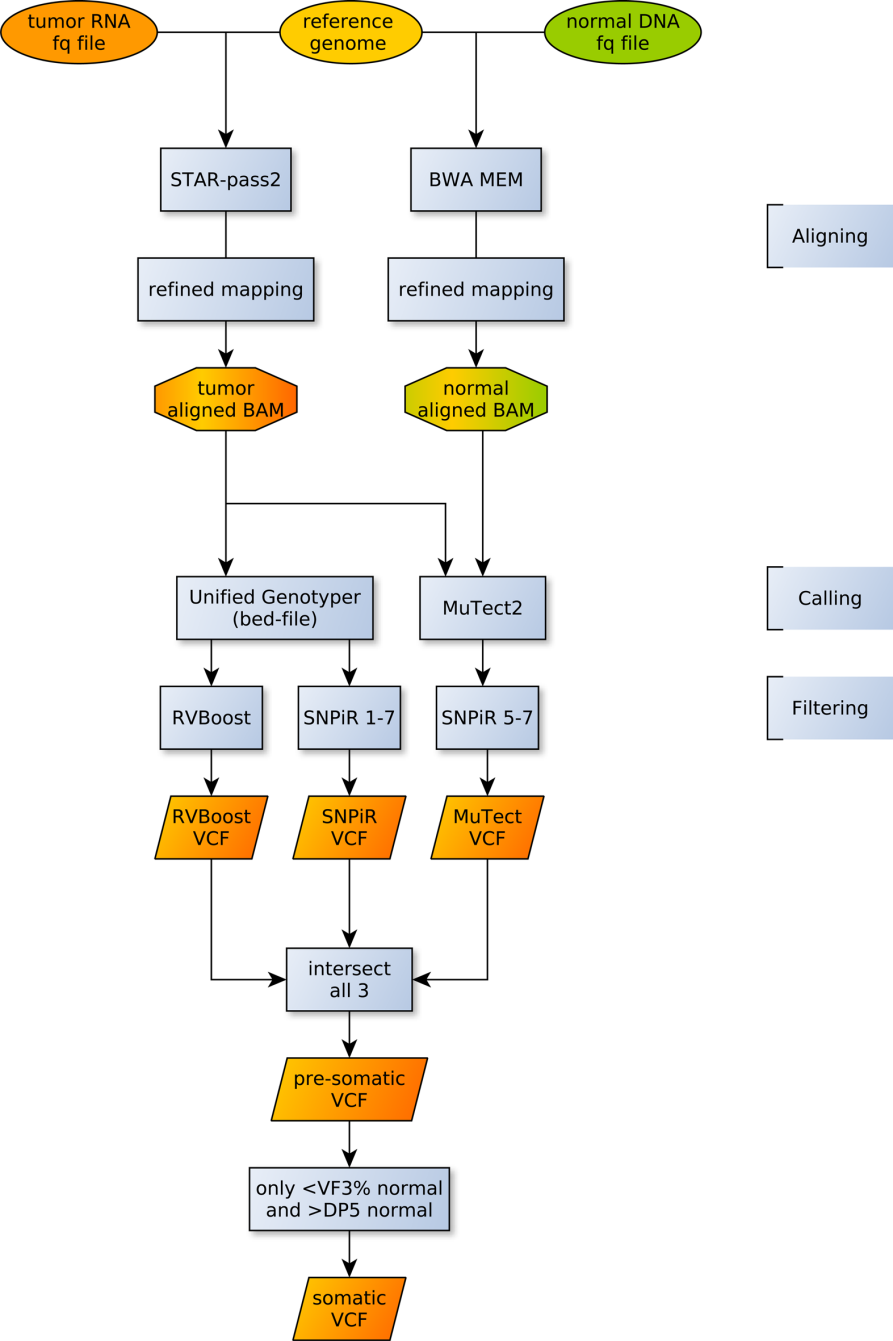
VaDiR workflow for processing somatic variant calls from RNA-seq. Sequence alignment is done by STAR and BWA MEM for RNA and DNA, respectively. The refined mapping follows GATK Best Practices. The variant calling is done by Unified Genotyper (GATK) and MuTect2 (GATK). The following filtering steps are done by RVBoost and SNPiR. Additional filters such as MAQ > 40, germline read depth (DP) > 5, and germline variant fraction (VAF) < 0.03 are applied to remove germline variants.

Because we observed differences in the VAF at the discordant sites, we next expanded the analysis to all sites. Interestingly, we observed a weak correlation of VAF between tumor DNA and tumor RNA at positions with DP>0 for tumor DNA and RNA (Fig. 4A). When we limit the analysis to positions with DP>10 for tumor DNA (Fig. 4B) or tumor and normal DNA (Fig. 4C), we also observed a weak correlation. Finally, when we limit the analysis to positions with DP>10 for tumor DNA and RNA and normal DNA, we observed a strong correlation of 0.74 of variant allele fraction between RNA and DNA (Fig. 4D). Only 4 mutations had VAF around 0.50 at the DNA level but 1.0 at the RNA level, which suggests that these are imprinted genes. These results suggest that VAFs in abundant transcripts are strongly correlated with VAFs at the DNA level. Therefore, a VAF obtained from RNA-sequencing may be used as a substitute for DNA VAF for subclone phylogenetic analysis. As shown by McPherson et al. [26], subclonal phylogenetics can use limited/targeted sequencing to identify subclones.

### Detection of artificial spiked variants

To further assess the performance of RNA-based callers, we used BamSurgeon and spiked-in 200 artificial RNA sequence variants at varying variant fractions in transcriptomes from 3 samples of 2 different tumor sites from one patient. From the 200 simulated variant positions, 120 were actually spiked in because failed positions have too low a read depth even if the positions for spiking were obtained from expressed genes. On average, 71% of all spiked-in variants were found by each caller alone. The combination of all 3 callers leads to a calling of around 50% of all spiked-in mutations (Table 2, Supplementary Fig. 7). By using Tier 2, we were able to call 60% of all spiked-in mutations; 55.6% of the mutations missed by Tier 1 but called by at least 1 caller are not in coding regions (Table 3). From the remaining missed variants, 15.7% have a variant allele fraction of less than 0.2 and 6.1% have high variant allele fraction but have a DP <10 in DNA.

**Table 3:** Characteristics of missed spiked-in variants

Tier 1	OV10	OV11	OV12
All spiked in variants	125	116	119
Missed by VaDiR	57	55	61
Not called by at least 1 caller	20	20	20
Missed in coding region	16	17	18
Missed in coding region	11	9	13
by RNA VAF>20%			
Missed in coding region	8	7	11
by RNA VAF>20%			
and normal DNA DP>10			
Tier 2	OV10	OV11	OV12
All spiked in variants	125	116	119
Missed by VaDiR	47	48	50
Not called by at least 1 caller	20	20	20
Missed in coding region	9	11	12
Missed in coding region	6	5	9
by RNA VAF>20%			
Missed in coding region	4	4	8
by RNA VAF>20%			
and normal DNA DP>10			

### Comparison between RADIA and VaDiR

As RADIA performs a function similar to our workflow VaDiR, we compared the performance differences between RADIA and VaDiR. RADIA uses DNA variant calling as the primary method and RNA variant calling as a supplement. All somatic variants called by RADIA are supported by DNA-level evidence, and RNA-only variants are not called by RADIA. Therefore, we limited our comparison with variants that are found at both RNA and DNA levels by RADIA and VaDiR. A total of 308 mutations were called by either RADIA or VaDiR or both in 6 samples. Of these, 175 mutations were called by both methods, 12 mutations were called by VaDiR only, and 121 mutations were called by RADIA only, while VAFs of variants missed by VaDiR are significantly lower than VAFs of variants missed by RADIA (Supplementary Fig. 8). From these 121 mutations, 40 (33.1%) had a read depth <10 in RNA. Fifty-two (43.0%) mutations, with a read depth over 10, had VAFs below 0.20. This shows again the limitation of the method based only on RNA. Six of the remaining 29 variants were in non-exonic regions and would not be called by our method.

### Ovarian cancer: resistant vs sensitive

As variant calling from RNA-seq provides both mutational status and gene expression, the number of mutations found by RNA-seq may be associated with pathologic or clinical phenotypes. In contrast, the total number of mutations found at the DNA level may not be associated with pathologic or clinical phenotype because it may be confounded by potentially non-relevant mutations in non-coding regions, or in genes that are not expressed. To determine if variant calling from RNA-sequencing may provide novel insights into clinical phenotype, we characterized the number of mutations in expressed genes from RNA-seq obtained from 10 chemotherapy-resistant and 11 chemotherapy-sensitive ovarian carcinomas. We considered concordant mutations only (those found by both RNA- and DNA-based callers) for the analysis. The results indicate that the concordant rate is higher for Tier 1 mutations compared with Tier 2 mutations, although the total number of mutations is higher in Tier 2 (Fig. 5A). We observed a higher amount of mutations in chemotherapy-sensitive ovarian carcinomas compared with their chemotherapy-resistant counterparts (Fig. 5A). This result is consistent with previous studies indicating that sensitive tumor samples have a higher mutation rate in ovarian cancer [27]. In these samples, the number of mutations was significantly higher at either DNA (*P* = 0.017, 2-sample *t* test, *t* = −2.3474, *df* = 19) or RNA (*P* = 0.03, 2-sample *t* test, *t* = −2.605, *df* = 19) levels in sensitive carcinomas compared with resistant carcinoma samples (Fig. 5B).

We next focus our analysis on variants that produce nonsynonymous mutations because they are more likely to contribute to a change in phenotype and the divergent evolution of tumor subclones. If a tumor sample is predominantly represented by a tumor subclone, the VAF of nonsynonymous SNVs in that subclone will provide the largest fraction of mutations, and thus higher fractions of VAF in nonsynonymous SNVs is expected. On the other hand, if the tumor sample is represented by multiple tumor subclones, each containing subclone-specific mutations, nonsynonymous SNVs will be found at low levels in this tumor. Therefore, the VAF of nonsynonymous mutations may represent clonal heterogeneity. Results, shown in Supplementary Fig. 9, indicate that differences in VAF between sensitive and resistant samples are not significant. Interestingly, sensitive samples have significantly lower VAFs in non-COSMIC mutations compared with resistant samples both at the RNA (*P* = 0.034, 2-sample *t* test, *t* = 2.1681, *df* = 62) and DNA levels (*P* = 0.017, 2-sample *t* test, *t* = 2.4543, *df* = 62)(Supplementary Fig. 9B).

## Discussion

In addition to the consensus calling of variants by 3 methods, we tested weighted combinations of the 3 methods with and without considering the VAF [28]. We didn’t see any improvements in the numbers of true-positive variants, false-negative variants, or false-positive variants. Therefore, the approach that uses weighted average features is not implemented in our tool. However, our workflow provides the possibility of combining calls from any or all callers for further refinement or for adapting to the needs of users.

With our approach, we were able to call variants with high precision. Only a small fraction of the variants that are called in RNA but not in DNA are likely false-positives. The remaining discordant variants are either RNA-editing sites or are missed by DNA callers. Most of the variants called in DNA but missed by VaDiR are not in coding regions or are not expressed. We also missed many variants that have low VAF. Those are called by none of the callers, MuTect2 only, or SNPiR only. These mutations are observed at low VAFs in tumor DNA, and therefore, they likely represent mutations from small subsets of tumor subclones. Finally, our approach missed approximately 15% of variants (127/853) with a high DP and a high VAF. Among the 127, 96 mutations were called by at least 1 method, indicating that consensus calling is too stringent or that parameters for 1 of the callers are not optimal. Those data are confirmed by the artificial spiked-in variants in which only variants with high VAF could be called by all 3 callers.

The comparison with RADIA shows that VaDiR misses mainly low-frequency RNA variants while RADIA misses some high-frequency RNA variants. This result confirms the limitation of calling variants only from RNA, but it also shows that VaDiR can be used to call a great number of somatic variants without the need for tumor whole-exome sequencing. It should be noted thats current workflow is not completely independent of DNA sequencing as we use germline DNA sequencing to filter out germline variants. However, if the goal is to discover variants in RNA sequencing, the VaDiR workflow can be modified to use MuTect2 without germline DNA and to leave out the last filtering step for DP and VAF values in germline DNA. VaDiR may be suitable for tiered studies where VaDiR can be used in the initial step to identify common variants from RNA sequencing datasets, and these candidate mutations can be confirmed by targeted DNA sequencing in a larger cohort to uncover biologically relevant somatic mutations for a specific cancer type. By focusing the initial variant discovery to expressed genes in diseased samples, follow-up validation sequencing efforts can be more targeted to limited regions of interest, thereby lowering the total cost of these genomic studies.

We were also able to find new possible RNA-editing sites, which should be investigated in future studies. Therefore, our workflow provides new capabilities that are missing in existing approaches and can be used to gain novel insight into disease phenotype. Our main concern in future studies would be to increase the number of concordant variant calls by adjustment of the filtering steps from SNPiR and RVboost and to investigate the reasons for missed somatic variants with high VAFs. Future work will also include efforts to make this tool available through a web server for the detection of somatic variants in RNAseq.

## Methods

### Software

To process the data, we used STAR, BWA-MEM, Genome Analysis Toolkit (GATK), SNPiR, RVboost, R, Picard, BEDtools, ANNOVAR, SAMtools, and BCFtools, which is a part of the SAMtools package (Supplementary Table 3) [2, 12, 13, 29–36]. To analyze our results, we used BAMSurgeon, R, and RADIA [15, 37]. We used reference files from the Broad Institute’s resource bundle [38], including the UCSC hg19 (GRCh37) reference genome, known indels from the 1000 Genomes Project, and known SNPs from dbSNP.

To validate the results that we obtained from RNA, we used somatic variants from DNA called by any 2 of the variant callers MuSE, MuTect2, SomaticSniper, and VarScan. We retrieved the corresponding VCF files from GDC [22].

We implemented SNPiR with the following modifications: In the file BLAT_candidates.pl at line 94, the developers incorrectly handled the information in the CIGAR-string of hardclipped reads, which resulted in a faulty shift in the base position. We corrected the code to handle CIGAR-strings correctly. This modification was necessary because our workflow differs from the SNPiR workflow in that we use hard-clipped reads. At the same location, we also added an optimization to avoid searching through more base positions than necessary. Further, we changed the filter to use PBLAT instead of BLAT, so we could utilize additional CPU threads to improve execution time. We made similar changes in the file filter_mismatch_first6bp.pl at line 84. In addition, we optimized the search algorithm in filter_intron_near_splicejuncts.pl by skipping the exons and genes that do not contain a given variant position (which also introduced the requirement that SNPiRs gene annotation table be sorted by position) and moderately improved code for readability. Finally, we modified convertVCF.sh to filter out any variant whose read depth (DP) value was 0, in order to prevent division-by-0 errors that occurred with our dataset. Rather than replacing the original SNPiR files in our distribution, we have included both versions and prefixed our file names with “revised_”.

For comparison with our method, we implemented RADIA with the following modification: During BLAT filtering, RADIA also incorrectly handled the hard-clipped reads. We corrected the code for the same reasons as described for the SNPiR implementation.

For creation of the figures, the R package ggplot2 [39] was used.

### Aligning sequences

The procedure for the alignment to the reference genome followed GATK Best Practices (Fig. 6) [40, 41]. For RNA-seq, we used the STAR aligner in 2-pass mode with the parameters implemented by the ENCODE project. The resulting aligned reads were processed to add read groups, sort, mark duplicates, split reads that spanned splice junctions, create an index, realign around known indels, reassign mapping qualities, and recalibrate base quality scores.

For DNA, we used the BWA-MEM aligner with the same reference genome. The resulting aligned reads were processed to add read groups, sort, mark duplicates, create an index, realign around known indels, reassign mapping qualities, and recalibrate base quality scores.

### Calling variants

A refined BAM file for each sample is then used to process the variant calling. Three different methods for calling are used: RVboost, SNPiR, and MuTect2. The first 2 methods are for germline variants in RNA, and the last method is for somatic variants in DNA. None of these methods is for somatic variant calling in RNA. RVboost and SNPiR use the same variant caller, UnifiedGenotyper from GATK, but different filtering procedures. RVboost filters variants using a statistical learning method called boosting, whereas SNPiR uses hard filtering in 7 steps (Supplementary Table 4). To adapt MuTect2’s results for RNA, we implemented 3 SNPiR’s hard-filtering steps. RVboost and SNPiR only need the refined RNA BAM file from the tumor tissue. MuTect2 needs both the refined RNA BAM from the tumor tissue and the refined DNA BAM from normal tissue.

### Filtering somatic variants by caller intersection and additional hard filters

In addition to the filtering procedures of the variant callers themselves, we further filtered our results by taking an intersection of vcf files from the 3 callers. We restricted our final, combined callset to the variants called by all 3 methods (Tier 1) or supplemented by variants called by MuTect2 and SNPiR (Tier2). We also applied our own hard filters, only accepting variants with a read depth (DP) of at least 5 and a VAF of less than 3% in uniquely mapping reads (Mapping quality of at least 40) in the normal DNA at the corresponding position.

### Weighting of Features

For the performance of different weighted combinations of the 3 callers, namely SNPiR (s), Rvboost (r), and MuTect2 (m), we performed 2 experiments using all variants in coding regions that have a read depth DP >10 in RNAseq. The weight *w_i_* of caller *i* was calculated as follows: $w_i=\frac{v_i}{\sum _{j=1}^3 v_j}$, where the values *v_i_* ranged from 0 to 1 in increments of 0.1. To find the best weighted combination, we determined an evalue, which is calculated as sum of all false-negative and all false-positive variants. Next, we calculated the area under the precision-recall curve (AUPRC), sensitivity/recall, specificity, and precision.

Experiment 1 (Supplementary Table 5): For all variants called by at least 1 caller, we calculated the weighted score *s* as follows: $s=\sum _{i=1}^3 w_i\cdot c_i$, where *c_i_* represents call (1) or no call (0) made by caller *i*. We then identified the optimal threshold of *s* that provides the lowest evalue. This was done for each weighted combination of callers.

Experiment 2 (Supplementary Table 6): We calculated the evalue for each weighted combination to determine the optimal threshold for which the variant is called multiplied by the variant allele frequency (*VAF*) and adjusted to the dynamic range of the callers as follows: $s=\sum _{i=1}^3 w_i\cdot \frac{vaf_i - mean_i}{standard deviation_i}$. The threshold is a value of *s* between −3 and 3 at which the lowest evalue is achieved.

### Processing artificial spiked variants

We used BAMSURGEON to spike-in 200 variants in coding regions of 2 ovarian tumor samples, such that each sample had a different random frequency of spiked-in variants. The samples were then processed by VaDiR.

### Processing samples with RADIA

Six samples from TCGA, 3 from resistant patients and 3 from sensitive patients, were processed with RADIA. This analysis required 3 BAM files from each sample: 1 from normal blood DNA, 1 from tumor DNA, and 1 from tumor RNA. We followed the instructions provided by RADIA for filtering. We used all possible filters provided by RADIA.

## Availability and Requirements

- Project name: somatic VaDiR
- Project home page: http://dx.doi.org/10.5524/100360
- Project RRID:SCR_015797
- Operating system(s): Linux/Unix 64-Bit
- Programming language: Perl, R, Java, Shell
- Other requirements: Java 7 and 8, R 3.3 or higher
- License: MIT
- Any restrictions to use by non-academics: no

## Availability of Supporting Data and Materials

The datasets supporting the results of this article are available in the open science framework repository [42] and the GDC repository [22].

Supporting data and an archival copy of the code are also available via the *GigaScience* repository, *Giga*DB [43].

For testing purposes, we utilized data kindly provided by Dr. Andrea Mariani of Mayo Clinic, Rochester, Minnesota. Due to ethical constraints, these data cannot be shared publicly, but if researchers would like to request access to these data, please contact Dr. Andrea Mariani (mariani.andrea@mayo.edu) with a short description of why you require access and how you would use the data.

## Additional Files

Supporting data are included in Supplementary Figs S1–S9 and Supplementary Tables S1–S6.

Supplementary Figure S1. Venn-Diagram of evalues of different combinations of callers. Different weighting in one combination of callers leads to the same evalue.

Supplementary Figure S2. Precision-Recall-Curve of the two Experiments with weighting of the callers, where red is Experiment 1 - consider only if the caller has called the variant or not - and blue is Experiment 2 - consider next to the calling the vaf of the called variant. Here we can see that even the best combination of Experiment 2 has a worse recall than each of the combinations of Experiment 2.

Supplementary Figure S3. Characteristic of all mutations called in tumor DNA by at least two callers, but not by VaDiR. Number of variants are shown in brackets. RNA(t) VAF indicates variant frequency in tumor RNA.

Supplementary Figure S4. Pie chart of number of variants within three specific variant allele frequency (VAF) range produced from SAMtools mpileup with MAQ >= 40. We included all positions called in tumor DNA by at least two callers by TCGA but not by VaDiR with DP>10 in RNA. Those 141 positions with variant frequency = 0 in DNA are the results of filtering out reads with MAQ < 40. These positions may represents false-positive calls by TCGA.

Supplementary Figure S5. Characteristics of all mutations called in RNA by VaDiR but not in tumor DNA by at least two callers. Number of variants are shown in brackets. DP indicates read depth, VAF indicates variant allele frequency, and (t) and (n) indicate tumor and normal respectively.

Supplementary Figure S6. Numbers of specific types of mutations with variant allele frequency = 0 in DNA and DP<10 in tumor DNA, RNA and normal DNA. Note that out of 10 A<G variants, 1 is recurrent in 4 samples. Out of 10 C<T variants, 1 is recurrent in 9 samples. These sites likely represents novel RNA-editing sites.

Supplementary Figure S7. Violin plot of variant allele fraction (VAF) of spiked-in variants. Called and missed variants are shown in different colors. Number of variants are shown in brackets

Supplementary Figure S8. Violin plot of variant allele fraction of calls made by RADIA or VaDiR in 6 TCGA samples. Note that most of the variants missed by VaDiR has low vaf whereas those missed by RADIA has high variant fraction.

Supplementary Figure S9. Variant fraction of nonsynonymous SNVs in ovarian tumor samples.

(A) Variant allele fraction of SNVs in DNA and RNA are not siginificantly different between sensitive and resistant tumor samples.

(B) Most of the variants have similiar variant allele fraction between DNA and RNA.

Supplementary Table S1. Sample Ids for TCGA samples used in the study.

Supplementary Table S2. Performance of the calling tools and specific combinations of the tools.

Supplementary Table S3. Software used in the study.

Supplementary Table S4. Filtering steps.

Supplementary Table S5. Optimal measures of prediction performance based on weighted combinations of variant calling classifiers.

Supplementary Table S6. Optimal measures of prediction performance based on weighted combinations of variant calling classifiers utilizing the variant allele frequency.

## Abbrevations

DP: read depth; RNA-seq: data from sequencing cDNA derived from RNA; Tier 1: variants called by each caller (SNPiR, RVBoost, MuTect2); Tier 2: variants called by Tier 1 and variants called by SNPiR and MuTect2; VAF: variant allele fraction; WES: Whole-exome sequencing; WGS: Whole-genome sequencing.

## Ethics approved and consent to participate

The datasets were obtained from the Cancer Genome Atlas, and the use of data was approved under the Project #4017 at the database of Genotypes and Phenotypes (dbGaP).

## Competing interests

The authors declare that they have no competing interests.

## Funding

The study was funded by the University of Kansas Endowment Association, the University of Kansas Cancer Center Support Grant (P30-CA168524), the Biostatistics and Informatics Shared Resource (BISR), the Cancer Center Cancer Biology program, and the Department of Defense Ovarian Cancer Research Program under award number (W81XWH-10-1-0386). The views and opinions herein and endorsements by the author(s) do not reflect those of the US Army or the Department of Defense.

## Author contributions

- Development of workflow: Jeremy Chien and Lisa Neums
- Conception and design: Jeremy Chien and Lisa Neums
- Acquisition of data: Andrea Mariani
- Analysis and interpretation of data: Lisa Neums, Jeremy Chien, Seiji Suenaga, Devin Koestler, and Sara Anders
- Writing, review, and revision of the manuscript: Jeremy Chien, Lisa Neums, and Seiji Suenaga
- Administration, technical, or material support: Jeremy Chien, Peter Beyerlein and Devin Koestler

## Supplementary Material

GIGA-D-16-00160_Original_Submission.pdf

GIGA-D-16-00160_Revision_1.pdf

GIGA-D-16-00160_Revision_2.pdf

GIGA-D-16-00160_Revision_3.pdf

Response_to_Reviewer_Comments_Original_Submission.pdf

Response_to_Reviewer_Comments_Revision_1.pdf

Response_to_Reviewer_Comments_Revision_2.pdf

Reviewer_1_Report_(Original_Submission) -- Kou-Chen Chou 15 Jan 2017 Reviewed

Reviewer_2_Report_(Original_Submission) -- Jiarui Ding 26 Jan 2017 Reviewed

Reviewer_2_Report_(Revision_1) -- Jiarui Ding 19 Jun 2017 Reviewed

Reviewer_3_Original_Submission_(Attachment).pdf

Reviewer_3_Report_(Original_Submission) -- Terry Speed 03 Mar 2017 Reviewed

Reviewer_3_Report_(Revision_1) -- Terry Speed 06 Jun 2017 Reviewed

Reviewer_3_Report_(Revision_2) -- Terry Speed 11 Sep 2017 Reviewed

Supplemental material
