# Supplementary material for: VaDiR: an integrated approach to Variant Detection in RNA
Source: Gigascience. 2017 Dec 18;7(2):1–13. doi: 10.1093/gigascience/gix122 (PMC5827345; doi:10.1093/gigascience/gix122)
Supplement: GIGA-D-16-00160_Original_Submission.pdf [file gix122_giga-d-16-00160_original_submission.pdf]

GigaScience

VaDiR: an integrated approach to Variant Detection in RNA

--Manuscript Draft--

**Manuscript Number:**	GIGA-D-16-00160
**Full Title:**	VaDiR: an integrated approach to Variant Detection in RNA
**Article Type:**	Technical Note
**Funding Information:**	National Cancer Institute (P30-CA168524) — Not applicable<br>Department of Defense Ovarian Cancer Research Program (W81XWH-10-1-0386) — Dr. Jeremy Chien
**Abstract:**	Background<br>Advances in next-generation DNA sequencing technologies are now enabling detailed characterization of sequence variations in cancer genomes. With whole genome sequencing, variations in coding and non-coding sequences can be discovered. But the cost associated with it is currently limiting its general use in research. Whole exome sequencing is used to characterize sequence variations in coding regions, but the cost associated with capture reagents and biases in capture rate limit its full use in research. Additional limitations include uncertainty in assigning the functional significance of the mutations when these mutations are observed in the non-coding region or in genes that are not expressed in cancer tissue.<br><br>Results<br>We investigated the feasibility of uncovering mutations from expressed genes using RNA sequencing datasets with a method called "VaDiR: Variant Detection in RNA" that integrate three variant callers, namely: SNPiR, RVBoost and MuTect2. The combination of all three methods, which we called Tier1 variants, produced the highest specificity with true positive mutations from RNA-seq that could be validated at the DNA level. We also found that the integration of Tier1 variants with those called by MuTect2 and SNPiR produced the highest sensitivity with acceptable specificity. Finally, we observed higher rate of mutation discovery in genes that are expressed at higher levels.<br><br>Conclusions<br>Our method, VaDiR, provides a possibility of uncovering mutations from RNA sequencing datasets that could be useful in further functional analysis. In addition, our approach allows orthogonal validation of DNA-based mutation discovery by providing complementary sequence variation analysis from paired RNA sequencing data sets.
**Corresponding Author:**	Jeremy Chien, PhD<br>University of Kansas Medical Center<br>Kansas City, KANSAS UNITED STATES
**Corresponding Author Secondary Information:**	
**Corresponding Author's Institution:**	University of Kansas Medical Center
**Corresponding Author's Secondary Institution:**	
**First Author:**	Lisa Neums
**First Author Secondary Information:**	
**Order of Authors:**	Lisa Neums<br>Seiji Suenaga<br>Peter Beyerlein<br>Andrea Mariani<br>Jeremy Chien

Powered by Editorial Manager® and ProduXion Manager® from Aries Systems Corporation

**Order of Authors Secondary Information:**	
**Opposed Reviewers:**	
**Additional Information:**	
**Question**	**Response**
Are you submitting this manuscript to a special series or article collection?	No
**Experimental design and statistics**<br><br>Full details of the experimental design and statistical methods used should be given in the Methods section, as detailed in our Minimum Standards Reporting Checklist. Information essential to interpreting the data presented should be made available in the figure legends.<br><br>Have you included all the information requested in your manuscript?	Yes
**Resources**<br><br>A description of all resources used, including antibodies, cell lines, animals and software tools, with enough information to allow them to be uniquely identified, should be included in the Methods section. Authors are strongly encouraged to cite Research Resource Identifiers (RRIDs) for antibodies, model organisms and tools, where possible.<br><br>Have you included the information requested as detailed in our Minimum Standards Reporting Checklist?	Yes
**Availability of data and materials**<br><br>All datasets and code on which the conclusions of the paper rely must be either included in your submission or deposited in publicly available repositories (where available and ethically appropriate), referencing such data using a unique identifier in the references and in the "Availability of Data and Materials" section of your manuscript.<br><br>Have you have met the above requirement as detailed in our Minimum Standards Reporting Checklist?	Yes

Additional file Click here to download Manuscript Reference PDF.pdf

RESEARCH

VaDiR: an integrated approach to Variant Detection in RNA

Lisa Neums[1,2], Seiji Suenaga[1], Peter Beyerlein[2], Andrea Mariani[3] and Jeremy Chien[1*]

Abstract

Background: Advances in next-generation DNA sequencing technologies are now enabling detailed characterization of sequence variations in cancer genomes. With whole genome sequencing, variations in coding and non-coding sequences can be discovered. But the cost associated with it is currently limiting its general use in research. Whole exome sequencing is used to characterize sequence variations in coding regions, but the cost associated with capture reagents and biases in capture rate limit its full use in research. Additional limitations include uncertainty in assigning the functional significance of the mutations when these mutations are observed in the non-coding region or in genes that are not expressed in cancer tissue.

Results: We investigated the feasibility of uncovering mutations from expressed genes using RNA sequencing datasets with a method called "VaDiR: Variant Detection in RNA" that integrate three variant callers, namely: SNPiR, RVBoost and MuTect2. The combination of all three methods, which we called Tier1 variants, produced the highest specificity with true positive mutations from RNA-seq that could be validated at the DNA level. We also found that the integration of Tier1 variants with those called by MuTect2 and SNPiR produced the highest sensitivity with acceptable specificity. Finally, we observed higher rate of mutation discovery in genes that are expressed at higher levels.

Conclusions: Our method, VaDiR, provides a possibility of uncovering mutations from RNA sequencing datasets that could be useful in further functional analysis. In addition, our approach allows orthogonal validation of DNA-based mutation discovery by providing complementary sequence variation analysis from paired RNA sequencing data sets.

Keywords: RNA-seq; somatic variant calling; Ovarian Cancer; Cancer genomes; Transcriptome

Background

Next-generation sequencing has enabled the discovery of novel variants in genetic sequences. However, even though the cost of sequencing has decreased in recent years, whole genome sequencing (WGS) can still be prohibitively expensive in many cases [1]. Sequencing only exonic regions of the genome helps reduce cost, and multiple tools (such as MuTect2 provided by GATK [2], MuSE [3], SomaticSniper [4] and VarScan2 [5]) have been developed for somatic variant discovery using whole exome sequencing (WES) data. Still, the reagents used to capture exonic regions are costly and produce uneven coverage across the genome due to capture rate biases [6, 7], and few of the genes in an exome are actually expressed in any given cell [8]. For diseases like cancer, mutations in expressed regions are of greater interest than in non-exonic or unexpressed exonic regions because they are more likely to affect cell function directly. The transcriptome is therefore an attractive subject of research in cancer and other human pathologies, and some of the cancer genes, such as FOXL2 in granulosa-cell tumors [9] and ARID1A in clear cell carcinomas of the ovary [10], were initially discovered through transcriptome sequencing.

The calling of variants with sequencing data from transcriptome (RNA-seq) is more challenging because of the splice junctions. Tools like RVBoost [11], SNPiR [12] or GATK Haplotypecaller are created to address this problem. Somatic variant calling from RNA is more difficult because of RNA processing like RNA-editing, allele-specific expression, variable levels of gene expression, and the heterogeneity of tumors which leads to low variant frequencies of some mutations [13]. Tools such as RVBoost, SNPiR, and GATK Haplotypecaller can be used to perform variant calling from RNA, but their performance and limitations for so-

*Correspondence: jchien@kumc.edu
[1]Department of Cancer Biology, University of Kansas Medical Center, 3901 Rainbow Blvd., 66160 Kansas City, KS, USA
Full list of author information is available at the end of the article

matic variant calling have not been studied previously. Nonetheless, these approaches have the potential to provide an orthogonal method to validate DNA sequence variations by complementing the analysis with RNA sequence analysis.

It should be noted that the integrated approach used by RADIA [14], that combines the variant sequence analysis from DNA and RNA sequencing, allows discovery of DNA sequence variations in expressed genes and better characterization of the effect of mutations on gene expression and phenotypic alterations, but its use of WES introduces cost. The limitation of RADIA is that it requires DNA sequencing data, and RNA sequence analysis was used just as a supplement. Moreover, DNA sequence variations are considered as the ground truth, and RNA variants not supported by DNA sequencing were rejected as false-positives. Although variants discovered only by RNA sequencing have the potential of being false-positives, some of these variants may represent missed calls from DNA sequencing or RNA-editing sites that have not been annotated. A detailed comparison of DNA and RNA variants from different tools will provide us with more precise processing and discovery of sequence variations from RNA and DNA sequencing.

In this study, we performed a detailed comparison of DNA and RNA sequence variations from 21 pairs of whole exome and mRNA sequencing from ovarian cancer genomes. We described variants discovered in RNA-seq through three publicly available tools, namely MuTect2, RVboost and SNPiR, and developed the best combination of these tools that enables discovery of variants from RNA sequence with high precision and sensitivity. We also show that most of the variants which would be classified as false-positives or false-negatives can be explained by biological characteristics.

DATA DESCRIPTION

Twenty one samples of ovarian serous cystadenocarcinoma from The Cancer Genome Atlas (TCGA) were divided into two groups: 11 cases that were sensitive to the cancer treatment and 10 cases that were resistant. Sensitive cases had a progression-free survival of more than 18 months, and resistant cases had progression-free survival of less than 12 months. The clinical data for the patients were retrieved from cBioPortal ([15–17]) and the Illumina sequence files for tumor RNA and normal blood DNA were retrieved from cghub [18] and gdc [19] (see Supplementary Table 3). Whole exome sequencing and mRNA sequencing datasets were available from each patient.

Additional data were provided by Dr. Andrea Mariani and came from 2 different tumor samples from a patient with serous ovarian carcinoma.

ANALYSIS

Performance characteristics of each method and different combinations of two or more methods

To describe the performance characteristics of each method, we performed variant calling using RVBoost, SNPiR, and MuTect2 separately. Each caller alone calls many variants which are not validated by DNA somatic variants (discordant calls), while SNPiR calls the most variants (see Figure 2(A)). Mutect2 provides the least amount of variant calls not supported by DNA sequencing compared to the other two methods. However, only 10% of variant calls made by Mutect2 was supported by DNA sequencing. These results indicate that any single caller is not adequate in discovering variants with high specificity. Therefore, we next tested if any combination of three calling methods will provide higher rate of variant calls supported by DNA sequencing. The combination of all three calling methods (Tier1) leads to 81.8% of variants which are validated by DNA somatic variants (concordant calls) (see Figure 2(B)). The combination of Tier1 with mutations called by Mutect2 and SNPiR (Tier2) leads to a higher sensitivity while the precision is still in a moderate range. For the following analysis, we concentrated only on Tier1.

Performance of a combined calling method

A total of 634 somatic mutations were called from 21 tumor samples. 518 mutations of them were concordant and 116 were discordant (see Table 1). On the DNA level, a total of 10099 mutations were called and 9864 of them were not called by our method.

Variants not found in RNA

To understand why variant calls from RNA sequencing missed a large majority of variant calls observed by DNA sequencing, we checked the properties of variants missed by RNA callers. From the 9864 missed somatic variants, 6949 (70.4%) were not in exonic regions (see Supplementary Figure 1). From the mutations in exonic regions, 2046 (20.9%) were missed because these variants are from genes with less abundant transcripts. The effect of transcript abundance on variants discovered from RNA-seq could also be observed in the percentage of concordant calls: 516 (15%) of the expressed mutations were called by Tier1 (see Figure 3 (A)) but when the expression is higher (DP>10) 34.6% of the somatic mutations were called. This confirms that an important factor in RNA-seq variant calling is the expression level.

Among the mutations found by DNA callers but missed by Tier1 from highly expressed genes, 594 (6.0%) of mutations in tumor DNA and 756 (7.7%) of mutations in tumor RNA had a variant fraction < 0.20

(see Figure 3 (B), Supplementary Figure 2). This result shows that one of the limitations of RNA-based variant calling methods is that it is highly dependent on the variant allele frequency. When the mutation has a low variant frequency at DNA level, it is possible to miss these mutations because of the heterogeneity of the tumor sample. From the variants with high expression and high variant allele frequency, thirty one mutations were not called by at least one of the callers. Ninety six mutations were filtered out by at least one of the callers because of potential evidence of germline variants or because the realigning step with PBLAT shows that these variants could come from mis-mapping. Most of the variants which are missed at a low variant frequency are called by MuTect2 or SNPiR alone (see Figure 3 (C)). By studying the filter steps in future work, we may be able to call a higher number of those variants.

Variants not found in DNA

The differences in coverage or allele fraction between DNA and RNA datasets could contribute to discordant calls. Therefore, we checked those attributes at discordant sites. Twenty four (20.7%) of the discordant mutations had a read depth (DP) of uniquely mapping reads under 10 at DNA level (see Sublementary Figure 3) and another 25 (21.6%) mutations had a variant allele frequency (VF) above zero at DNA level, indicating that these low level DNA variants were missed by DNA-based callers by TCGA. Twenty four variants with 0 variant fraction at DNA level but high DP in DNA normal, DNA tumor and RNA tumor were mostly either A>G or C>T (see Sublementary Figure 5). Those variants were found at 15 different positions, of which one variant (chr3:58141791 A>G [FLNB:p.M2324V]) is found in 4 different samples and another (chr20:10285837 C>T) in 9 different samples. These likely represent probably non-annotated RNA-editing sites [20–22].

Because we observed differences in the variant fraction at the discordant sites, we next expanded the analysis to all sites. Interestingly, we observed weak correlation of variant fraction between tumor DNA and tumor RNA at positions with DP>0 for tumor DNA and RNA (see Figure 4 (A)). When we limit the analysis to positions with DP>10 for tumor DNA (see Figure 4 (B)) or tumor and normal DNA (see Figure 4 (C)), we also observed a weak correlation. Finally, when we limit the analysis to positions with DP>10 for tumor DNA and RNA and normal DNA, we observed a strong correlation of variant fraction between RNA and DNA (see Figure 4 (D)). Only four mutations had variant frequencies around 50% at DNA level and 100% at RNA level which suggests that these are imprinted genes. These results suggest that variant fraction in abundant transcripts are strongly correlated with variant fractions at DNA level. Therefore, RNA variant fraction may be used as a substitute for DNA variant fraction for subclone phylogenetic analysis.

Detection of artificial spiked variants

To further assess the performance of RNA-based callers, we used BamSurgeon and spiked-in 200 artificial RNA sequence variants at varying variant fractions in two tumor transcriptomes. From the 200 simulated variant positions, 120 were actually spiked in because failed positions have too low read depth even if the positions for spiking were obtained from expressed genes. On average 71% of all spiked-in variants were found by each caller alone. The combination of all three callers leads to a calling of around 50% of all spiked-in mutations (see Table 2, Supplementary Figure 5). By using Tier2, we were able to call 60% of all spiked-in mutations. 55.6% of the mutations missed by Tier1 are not in coding regions (see Table 3). From the remaining missed variants, 15.7% have a variant allele fraction of less than 0.2 and 6.1% have high variant allele fraction but have a DP<10 in DNA.

Comparison between RADIA and VaDiR

Since RADIA performs function similar to our workflow VaDiR, we compared the performance differences between RADIA and VaDiR. RADIA uses DNA variant calling as the primary method and use RNA variant calling as a supplement. All somatic variants called by RADIA are supported by DNA-level evidence and RNA-only variants are not called by RADIA. Therefore, we limited our comparison to variants that are found at both RNA and DNA levels by RADIA and VaDiR. A total of 308 mutations were called by either RADIA or VaDiR or both in six samples. Of these, 175 mutations were called by both methods, 12 mutations were called by VaDiR only, and 121 mutations were called by RADIA only (see Supplementary Figure 6). From these 121 mutations, 40 (33.1%) had a read depth below 10 in RNA. 52 (43.0%) mutations, with a read depth over 10, had a variant fraction below 0.20. This shows again the limitation of method based only on RNA. Six of the remaining 29 variants were in non-exonic regions and would not be called by our method.

Ovarian cancer: resistant vs. sensitive

Since variant calling from RNA-seq provides both mutational status and gene expression, the number of mutations found by RNA-seq may be associated with pathologic or clinical phenotypes. In contrast, the total number of mutations found at the DNA level may

not be associated with pathologic or clinical phenotype because it may be confounded by potentially non-relevant mutations in non-coding region or in genes that are not expressed. To determine if variant calling from RNA-sequencing may provide novel insights into clinical phenotype, we characterized the number of mutations in expressed genes from RNA-seq obtained from 10 chemotherapy-resistant and 11 chemotherapy-sensitive ovarian carcinomas. We considered concordant mutations only (those found by both RNA- and DNA-based callers) for the analysis. The results indicate that concordant rate is higher for Tier1 mutations compared to Tier2 mutations although total number of mutations are higher in Tier2 (see Figure 5 (A)). We observed higher amount of mutations in chemotherapy-sensitive ovarian carcinomas compared to chemotherapy-resistant counterparts (see Figure 5 (A)). This result is consistent with previous studies indicating that sensitive tumor samples have a higher mutation rate in ovarian cancer [23]. In these samples, number of mutations at either DNA or RnA levels was significantly higher in sensitive carcinomas compared to resistant carcinoma samples (see Figure 5 (B)).

A higher mutation burden in sensitive tumors samples may be the result of high mutagenic processes in the cancer cells or the result of a high degree of intratumor heterogeneity or tumor subclones. If high levels of tumor subclones with clonal driver mutations are contributing to higher mutation burden in sensitive tumor samples, we expect these mutations will exist in lower variant fractions because they represent unique tumor subclones. Therefore, we limit our variant fraction analysis to variants that produce nonsynonymous mutations because they are more likely to contribute to a change in phenotype and the evolution of tumor subclones. Results, shown in supplementary Figure 7, indicate that differences in variant fractions between sensitive and resistant samples are not significant. Interestingly, sensitive samples have significantly lower variant allele fractions in non-COSMIC mutations compared to resistant samples both at the RNA (pValue=0.034) and DNA level (pValue=0.017)(see Supplementary Figure 7 (B)). These results suggest that these mutations are coming from subclonal populations. The higher levels of clonal heterogeneity and mutation burden in sensitive samples may be the result of defects in DNA repair, and this tumor cell characteristics may explain why they are sensitive to platinum-based chemotherapy.

DISCUSSION

With our approach, we were able to call variants with high precision. Only a small fraction of the variants which are called in RNA but not in DNA are likely false positives. The remaining discordant variants are either RNA-editing sites or are missed at the DNA level as well. Most of the variants called in DNA but missed by VaDiR are not in coding regions or are not expressed. We also missed many variants that have low variant frequency. Those are called by none of the callers, MuTect2 only, or SNPiR only. These mutations are observed at low variant frequencies in tumor DNA, and therefore they likely represent mutations from small subsets of tumor subclones. Finally, our approach missed approximately 15% of variants (127/853) with a high DP and a high variant allele frequency. Among those 127, 96 mutations were called by at least one method, indicating that consensus calling is too stringent or that parameters for one of the callers is not optimal. Those data are confirmed by the artificial spiked-in variants where only variants with high variant frequency could be called by all three callers.

The comparison to RADIA shows that we are missing mainly variants in low frequency ranges while RADIA is missing a few variants with high variant frequency in RNA. This confirms the limitation of calling variants only from RNA, but also shows that we are able to call a great number of somatic variants without the need for whole exome sequencing. We were also able to find new possible RNA-editing sites, which should be investigated in future studies. Therefore, our workflow provides new capabilities that are missing in existing approaches and can be used to gain novel insight into disease phenotype.

Our main concern in future studies would be to increase the number of concordant variant calls by adjustment of the filtering steps from SNPiR and RV-boost, and to investigate the reasons for the missed somatic variants with high variant allele frequency.

METHODS

Software

To process the data, we used the software STAR, BWA MEM, Genome Analysis Toolkit (GATK), SNPiR, RV-boost, R, Picard, BEDtools, ANNOVAR, SAMtools, and BCFtools which is a part of the SAMtools package [2, 11, 12, 24–31] (see Supplementary Table 1). To analyze our results, we used the software BAMSurgeon, R, and RADIA [14, 32]. We used reference files from Broad Institute's resource bundle [33], including the UCSC hg19 (GRCh37) reference genome, known indels from the 1000 Genomes Project, and known SNPs from dbSNP.

To validate the results that we obtained from RNA, we used somatic variants from DNA called by any two of the variant callers MuSE, MuTect2, SomaticSniper, and VarScan. We retrieved the corresponding VCF files from GDC [19].

We implemented SNPiR with the following modifications: In the file BLAT_candidates.pl at line 94, the developers incorrectly handled the information in the CIGAR-string of hardclipped reads, that resulted in a faulty shift of the read position. We corrected the code to handle CIGAR-strings correctly. This modification was necessary because our workflow differs from the SNPiR workflow in that we use hard-clipped reads. At the same location, we also added an optimization to avoid searching through more base positions than necessary. Further, we changed the filter to use PBLAT instead of BLAT, so we could utilize additional CPU threads to improve execution time. We made similar changes in the file filter_mismatch_first6bp.pl at line 84. In addition, we optimized the search algorithm in filter_intron_near_splicejuncts.pl by skipping exons and genes that do not contain a given variant position (which also introduced the requirement that SNPiR's gene annotation table be sorted by position) and moderately improve code for readability. Finally, we modified convertVCF.sh to filter out any variant whose read depth (DP) value was zero, in order to prevent division-by-zero errors that occurred with our dataset. Rather than replacing the original SNPiR files in our distribution, we have included both versions and prefixed our file names with "revised_".

For comparison with our method, we implemented RADIA with the following modification: During BLAT filtering, RADIA also incorrectly handled the hard-clipped reads. We corrected the code for the same reasons as described for the SNPiR implementation.

For creation of the figures, the R package ggplot2 [34] was used.

Aligning sequences

The procedure for the alignment to the reference genome followed GATK Best Practices [35, 36]. For RNA-seq, we used the STAR aligner in 2-pass mode with the parameters implemented by ENCODE project. The resulting aligned reads were processed to add read groups, sort, mark duplicates, split reads that spanned splice junctions, create an index, realign around known indels, reassign mapping qualities, and recalibrate base quality scores.

For DNA, we used the BWA MEM aligner with the same reference genome. The resulting aligned reads were processed to add read groups, sort, mark duplicates, create an index, realign around known indels, reassign mapping qualities, and recalibrate base quality scores.

Calling variants

A refined BAM file for each sample is then used to process the variant calling. Three different methods for calling are used: RVboost, SNPiR, and MuTect2. The first two methods are for germline variants in RNA and the last method is for somatic variants in DNA. None of these methods is for somatic variant calling in RNA. RVboost and SNPiR use the same variant caller, UnifiedGenotyper from GATK, but different filtering procedures. RVboost filters variants using a statistical learning method called boosting, whereas SNPiR uses hard filtering in 7 steps (see Supplementary Table 2). To adapt MuTect2's results for RNA, we implemented three of SNPiR's hard-filtering steps. RVboost and SNPiR only need the refined RNA BAM file from the tumor tissue. MuTect2 needs both the refined RNA BAM from the tumor tissue and the refined DNA BAM from normal tissue.

Filtering somatic variants by caller intersection and additional hard filters

In addition to the filtering procedures of the variant callers themselves, we further filtered our results by taking an intersection of vcf files from the three callers. We restricted our final, combined callset to the variants called by all three methods (Tier 1) or supplemented by variants called by MuTect2 and SNPiR (Tier2). We also applied our own hard filters, only accepting variants with a read depth (DP) of at least five and a variant allele frequency (VF) of less than 3% in uniquely mapping reads (Mapping quality of at least 40) in the normal DNA at the corresponding position.

Processing artificial spiked variants

We used BAMSURGEON to spike in 200 variants in coding regions of two ovarian tumor samples, such that each sample had a different random frequency of spiked-in variants. The samples were then processed by VaDiR.

Processing samples with RADIA

Six samples from TCGA, three from resistant patients and three from sensitive patients, were processed with RADIA. This analysis required three BAM files from each sample: one from normal blood DNA, one from tumor DNA, and one from tumor RNA. We followed the instructions provided by RADIA for filtering. We used all possible filters provided by RADIA.

AVAILABILITY AND REQUIREMENTS

- Project name: somatic VaDiR
- Project home page: e.g. http://to.be.added.later
- Operating system(s): Linux/Unix 64-Bit
- Programming language: Perl, R, Java, Shell
- Other requirements: Java 7 and 8, R 3.3 or higher
- License: MIT
- Any restrictions to use by non-academics: no

AVAILABILITY OF SUPPORTING DATA AND MATERIALS

The data sets supporting the results of this article are available in the open science framework repository, [37], and the GDC repository, [19].

List of abbrevations

- WGS: Whole genome sequencing
- WES: Whole exome sequencing
- RNA-seq: Data from sequencing cDNA derived from RNA
- Tier1: Variants called by each caller (SNPiR, RVBoost, MuTect2)
- Tier2: Variants called by Tier1 and variants called by SNPiR and MuTect2.
- VF: Variant fraction
- DP: read depth

Ethics approved and consent to participate

The datasets were obtained from the Cancer Genome Atlas, and the use of data was approved under the Project #4017 at dbGaP.

Consent for publication

Not applicable

Competing interests

The authors declare that they have no competing interests.

Funding

The study is funded by the University of Kansas Endowment Association, the University of Kansas Cancer Center Support Grant (P30-CA168524), the Cancer Center Cancer Biology program, and the Department of Defense Ovarian Cancer Research Program under award number (W81XWH-10-1-0386). Views and opinions of, and endorsements by the author(s) do not reflect those of the US Army or the Department of Defense.

Author's contributions

- Development of workflow: Jeremy Chien and Lisa Neums
- Conception and design: Jeremy Chien and Lisa Neums
- Acquisition of data: Dr. Andrea Mariani
- Analysis and interpretation of data: Lisa Neums, Jeremy Chien and Seiji Suenaga
- Writing, review, and revision of the manuscript: Jeremy Chien, Lisa Neums and Seiji Suenaga
- Administration, technical, or material support: Jeremy Chien and Peter Beyerlein

Acknowledgements

The results published here are in whole or part based upon data generated by the TCGA Research Network: http://cancergenome.nih.gov/. We acknowledge Dr. Devin Koestler for helpful discussions and comments.

Author details

[1]Department of Cancer Biology, University of Kansas Medical Center, 3901 Rainbow Blvd., 66160 Kansas City, KS, USA. [2]Department of Bioinformatics and Biosystems Technology, University of Applied Sciences Wildau, Hochschulring 1, 15745 Wildau, Germany. [3]Obstetrics and Gynecology, Cancer Center, Mayo Clinic, 200 First St. SW, 55905 Rochester, MN, USA.

References

1. The Cost of Sequencing a Human Genome. https://www.genome.gov/27565109/the-cost-of-sequencing-a-human-genome/
2. McKenna, A., Hanna, M., Banks, E., Sivachenko, A., Cibulskis, K., Kernytsky, A., Garimella, K., Altshuler, D., Gabriel, S., Daly, M., DePristo, M.A.: The genome analysis toolkit: a mapreduce framework for analyzing next-generation dna sequencing data. Genome Research **20**, 1297–1303 (2010)
3. Fan, Y., Xi, L., Hughes, D.S., Zhang, J., Zhang, J., Futreal, P.A., Wheeler, D.A., Wang, W.: Muse: accounting for tumor heterogeneity using a sample-specific error model improves sensitivity and specificity in mutation calling from sequencing data. Genome Biol. **17**(1), 178 (2016)
4. Larson, D.E., Harris, C.C., Chen, K., Koboldt, D.C., Abbott, T.E., Dooling, D.J., Ley, T.J., Mardis, E.R., Wilson, R.K., Ding, L.: Somaticsniper: identification of somatic point mutations in whole genome sequencing data. Bioinformatics **28**(3), 311–317 (2012)
5. Koboldt, D.C., Zhang, Q., Larson, D.E., Shen, D., McLellan, M.D., Lin, L., Miller, C.A., Mardis, E.R., Ding, L., Wilson, R.K.: Varscan 2: somatic mutation and copy number alteration discovery in cancer by exome sequencing. Genome Research **22**(3), 568–576 (2012)
6. Guettouche, T., Zuchner, S.: Improved coverage and accuracy with strand-conserving sequence enrichment. Genome Med **5**(5), 46 (2013)
7. Parla, J.S., Iossifov, I., Grabill, I., Spector, M.S., Kramer, M., McCombie, W.R.: A comparative analysis of exome capture. Genome Biol **12**(9), 97 (2011)
8. Garcia-Ortega, L.F., Martinez, O.: How many genes are expressed in a transcriptome? estimation and results for rna-seq. PLoS One **10**(6), 0130262 (2015)
9. Shah, S.P., Kobel, M., Senz, J., Morin, R.D., Clarke, B.A., Wiegand, K.C., Leung, G., Zayed, A., Mehl, E., Kalloger, S.E., Sun, M., Giuliany, R., Yorida, E., Jones, S., Varhol, R., Swenerton, K.D., Miller, D., Clement, P.B., Crane, C., Madore, J., Provencher, D., Leung, P., DeFazio, A., Khattra, J., Turashvili, G., Zhao, Y., Zeng, T., Glover, J.N., Vanderhyden, B., Zhao, C., Parkinson, C.A., Jimenez-Linan, M., Bowtell, D.D., Mes-Masson, A.M., Brenton, J.D., Aparicio, S.A., Boyd, N., Hirst, M., Gilks, C.B., Marra, M., Huntsman, D.G.: Mutation of foxl2 in granulosa-cell tumors of the ovary. N Engl J Med **360**(26), 2719–29 (2009)
10. Wiegand, K.C., Shah, S.P., Al-Agha, O.M., Zhao, Y., Tse, K., Zeng, T., Senz, J., McConechy, M.K., Anglesio, M.S., Kalloger, S.E., Yang, W., Heravi-Moussavi, A., Giuliany, R., Chow, C., Fee, J., Zayed, A., Prentice, L., Melnyk, N., Turashvili, G., Delaney, A.D., Madore, J., Yip, S., McPherson, A.W., Ha, G., Bell, L., Fereday, S., Tam, A., Galletta, L., Tonin, P.N., Provencher, D., Miller, D., Jones, S.J., Moore, R.A., Morin, G.B., Oloumi, A., Boyd, N., Aparicio, S.A., Shih Ie, M., Mes-Masson, A.M., Bowtell, D.D., Hirst, M., Gilks, B., Marra, M.A., Huntsman, D.G.: Arid1a mutations in endometriosis-associated ovarian carcinomas. N Engl J Med **363**(16), 1532–43 (2010)
11. Wang, C., Davila, J.I., Baheti, S., Bhagwate, A.V., Wang, X., Kocher, J.P., Slager, S.L., Feldman, A.L., Novak, A.J., Cerhan, J.R., Thompson, E.A., Asmann, Y.W.: Rvboost: Rna-seq variants prioritization using a boosting method. Bioinformatics **30**(23), 3414–3416 (2014)
12. Piskol, R., Ramaswami, G., Li, J.B.: Reliable identification of genomic variants from rna-seq data. Am J Hum Genet **93**(4), 641–651 (2013)
13. Spence, J.M., Spence, J.P., Abumoussa, A., Burack, W.R.: Ultradeep analysis of tumor heterogeneity in regions of somatic hypermutation. Genome Med **7**(1), 24 (2015)
14. Radenbaugh, A.J., Ma, S., Ewing, A., Stuart, J.M., Collisson, E.A., Zhu, J., Haussler, D.: Radia: Rna and dna integrated analysis for somatic mutation detection. PLoS One **9**(11) (2014)
15. cBioPortal for Cancer Genomics. http://www.cbioportal.org/
16. Gao, J., Aksoy, B.A., Dogrusoz, U., Dresdner, G., Gross, B., Sumer, S.O., Sun, Y., Jacobsen, A., Sinha, R., Larsson, E., Cerami, E., Sander, C., Schultz, N.: Integrative analysis of complex cancer genomics and clinical profiles using the cbioportal. Sci Signal **6**(269) (2013). pl1
17. Cerami, E., Gao, J., Dogrusoz, U., Gross, B.E., O, S.S., A, A.B., Jacobsen, A., Byrne, C.J., Heuer, M.L., Larsson, E., Antipin, Y., B, R., Goldberg, A.P., Sander, C., Schultz, N.: The cbio cancer genomics portal: An open platform for exploring multidimensional cancer genomics data. Cancer Discovery **2**(5), 401–404 (2012)
18. Cancer Genomics Hub. https://cghub.ucsc.edu/
19. GDC Data Portal - National Institutes of Health. https://gdc-portal.nci.nih.gov/
20. Wang, I.X., So, E., Devlin, J.L., Zhao, Y., Wu, M., Cheung, V.G.: Adar regulates rna editing, transcript stability, and gene expression. Cell Rep. **5**(3), 849–860 (2013)
21. Blanc, V., Davidson, N.O.: Apobec-1 mediated rna editing. Wiley Interdiscip Rev Syst Biol Med. **2**(5), 594–602 (2011)
22. Blanc, V., Park, E., Schaefer, S., Miller, M., Lin, Y., Kennedy, S., Billing, A.M., Ben Hamidane, H., Graumann, J., Mortazavi, A., Nadeau, J.H., Davidson, N.O.: Genome-wide identification and

functional analysis of apobec-1-mediated c-to-u rna editing in mouse small intestine and liver. Genome Biol **15**(6), 79 (2014)
23. Birkbak, N.J., Kochupurakkal, B., Izarzugaza, J.M.G., Eklund, A.C., Y, L., Liu, J., Szallasi, Z., Matulonis, U.A., Richardson, A.L., Iglehart, J.D., Wang, Z.C.: Tumor mutation burden forecasts outcome in ovarian cancer with brca1 or brca2 mutations. PLoS ONE (2013)
24. Wang, K., Li, M., Hakonarson, H.: Annovar: functional annotation of genetic variants from high-throughput sequencing data. Nucleic Acids Res **38**(16), 164 (2010)
25. Dobin, A., Davis, C.A., Schlesinger, F., Drenkow, J., Zaleski, C., Jha, S., Batut, P., Chaisson, M., Gingeras, T.R.: Star: ultrafast universal rna-seq aligner. Bioinformatics **29**(1), 15–21 (2013)
26. Li, H., Durbin, R.: Fast and accurate long-read alignment with burrows-wheeler transform. Bioinformatics **26**(5), 589–95 (2010)
27. Picard. http://broadinstitute.github.io/picard
28. Li, H.: A statistical framework for snp calling, mutation discovery, association mapping and population genetical parameter estimation from sequencing data. Bioinformatics **27**(21), 2987–93 (2011)
29. Li, H., Handsaker, B., Wysoker, A., Fennell, T., Ruan, J., Homer, N., Marth, G., Abecasis, G., Durbin, R., Genome Project Data Processing, S.: The sequence alignment/map format and samtools. Bioinformatics **25**(16), 2078–9 (2009)
30. Team, R.D.C.: R: A Language and Environment for Statistical Computing. R Foundation for Statistical Computing, Vienna, Austria (2008). R Foundation for Statistical Computing. http://www.R-project.org
31. Quinlan, A.R., Hall, I.M.: Bedtools: a flexible suite of utilities for comparing genomic features. Bioinformatics **26**(6), 841–2 (2010)
32. Ewing, A.D., Houlahan, K.E., Hu, Y., Ellrott, K., Caloian, C., Yamaguchi, T.N., Bare, J.C., P'ng, C., Waggott, D., Sabelnykova, V.Y., participants, I.-T.D.S.M.C.C., Kellen, M.R., Norman, T.C., Haussler, D., Friend, S.H., Stolovitzky, G., Margolin, A.A., Stuart, J.M., Boutros, P.C.: Combining tumor genome simulation with crowdsourcing to benchmark somatic single-nucleotide-variant detection. Nat Methods **12**(7), 623–30 (2015)
33. Broad Institute's Resource Bundle. ftp://ftp.broadinstitute.org/bundle/2.8/hg19/
34. Wickham, H.: Ggplot2: Elegant Graphics for Data Analysis. Springer, ??? (2009). http://ggplot2.org
35. DePristo, M., Banks, E., Poplin, R., Garimella, K., Maguire, J., Hartl, C., Philippakis, A., del Angel, G., Rivas, M.A., Hanna, M., McKenna, A., Fennell, T., Kernytsky, A., Sivachenko, A., Cibulskis, K., Gabriel, S., Altshuler, D., Daly, M.: A framework for variation discovery and genotyping using next-generation dna sequencing data. NATURE GENETICS **43**, 491–498 (2011)
36. Van der Auwera, G.A., Carneiro, M., Hartl, C., Poplin, R., del Angel, G., Levy-Moonshine, A., Jordan, T., Shakir, K., Roazen, D., Thibault, J., Banks, E., Garimella, K., Altshuler, D., Gabriel, S., DePristo, M.: From fastq data to high-confidence variant calls: The genome analysis toolkit best practices pipeline. CURRENT PROTOCOLS IN BIOINFORMATICS **43**, 11101–111033 (2013)
37. Open Science Framework Repository for VaDiR Data. http://www.osf.io/ap5b7

Illustrations and figures

Figure 1 Workflow of processing the variant call for somatic variants with RNA-seq. Aligning is done by STAR and BWA MEM and the refined mapping follows GATK Best Practices. The variant calling is done by Unified Genotyper (GATK) and MuTect2 (GATK). The following filtering steps are done by RVBoost and SNPiR.

Figure 2 Intersection of the three variant calling methods. (A) Intersection of the three methods with all somatic calls. The red arrows symbolize the amount of concordant calls. **(B)** Intersection of three methods with only concordant calls.

Figure 3 Variants called in tumor DNA. (A) Percentage of concordant calls of all somatic variants from expressed genes for each sample. With a high expression a higher percentage of concordant calls can be achieved. **(B)** Variant frequency in tumor DNA and RNA and normal DNA of all somatic variant called in DNA with a high expression (DP>10). **(C)** Comparison of somatic variants called in DNA which have high coverage in tumor DNA and RNA and normal DNA with variant calls from the three RNA callers. The names in the chart are the first letters of the caller SNPiR (s), RVBoost (r) and MuTect (m) or their combinations.

Figure 4 Correlation between the variant frequency of RNA and DNA. The four charts show different filtering steps of the read depth of tumor DNA and RNA and normal DNA.

Figure 5 Comparison of sensitive and resistant samples. (A) Test for significance of the difference in the variant allele frequency of nonsynonymous variants with DP>10 in DNA normal, DNA tumor and RNA tumor at tumor DNA and RNA level. **(B)** Comparison of variant frequency in DNA and RNA for each somatic variant.

Tables and captions

Table 1 Performance characteristics of VaDiR with the combination Tier1.

	DNA positive	DNA negative
RNA positive	518	116
RNA negative	9864	1595677

Table 2 Called spiked-in variants.

Sample	Tier1	Tier2
OV10	68 (52.71%)	78 (62.40%)
OV11	61 (52.59%)	68 (58.62%)
OV12	48 (48.74%)	69 (57.98%)

Table 3 Characteristics of missed spiked-in variants.

Tier1	OV10	OV11	OV12
all	105	96	99
missed	37	36	42
missed in coding region	16	17	18
missed in coding region by RNA VF>20%	11	9	13
missed in coding region by RNA VF>20% and normal DNA DP>10	8	7	11
**Tier2**	**OV10**	**OV11**	**OV12**
all	105	96	99
missed	27	29	31
missed in coding region	9	11	12
missed in coding region by RNA VF>20%	6	5	9
missed in coding region by RNA VF>20% and normal DNA DP>10	4	4	8

Manuscript Click here to download Manuscript VaDiR_bmc_rev.tex

RESEARCH

VaDiR: an integrated approach to Variant Detection in RNA

Lisa Neums[1,2], Seiji Suenaga[1], Peter Beyerlein[2], Andrea Mariani[3] and Jeremy Chien[1*]

Abstract

Background: Advances in next-generation DNA sequencing technologies are now enabling detailed characterization of sequence variations in cancer genomes. With whole genome sequencing, variations in coding and non-coding sequences can be discovered. But the cost associated with it is currently limiting its general use in research. Whole exome sequencing is used to characterize sequence variations in coding regions, but the cost associated with capture reagents and biases in capture rate limit its full use in research. Additional limitations include uncertainty in assigning the functional significance of the mutations when these mutations are observed in the non-coding region or in genes that are not expressed in cancer tissue.

Results: We investigated the feasibility of uncovering mutations from expressed genes using RNA sequencing datasets with a method called "VaDiR: Variant Detection in RNA" that integrate three variant callers, namely: SNPiR, RVBoost and MuTect2. The combination of all three methods, which we called Tier1 variants, produced the highest specificity with true positive mutations from RNA-seq that could be validated at the DNA level. We also found that the integration of Tier1 variants with those called by MuTect2 and SNPiR produced the highest sensitivity with acceptable specificity. Finally, we observed higher rate of mutation discovery in genes that are expressed at higher levels.

Conclusions: Our method, VaDiR, provides a possibility of uncovering mutations from RNA sequencing datasets that could be useful in further functional analysis. In addition, our approach allows orthogonal validation of DNA-based mutation discovery by providing complementary sequence variation analysis from paired RNA sequencing data sets.

Keywords: RNA-seq; somatic variant calling; Ovarian Cancer; Cancer genomes; Transcriptome

Background

Next-generation sequencing has enabled the discovery of novel variants in genetic sequences. However, even though the cost of sequencing has decreased in recent years, whole genome sequencing (WGS) can still be prohibitively expensive in many cases [1]. Sequencing only exonic regions of the genome helps reduce cost, and multiple tools (such as MuTect2 provided by GATK [2], MuSE [3], SomaticSniper [4] and VarScan2 [5]) have been developed for somatic variant discovery using whole exome sequencing (WES) data. Still, the reagents used to capture exonic regions are costly and produce uneven coverage across the genome due to capture rate biases [6, 7], and few of the genes in an exome are actually expressed in any given cell [8]. For diseases like cancer, mutations in expressed regions are of greater interest than in non-exonic or unexpressed exonic regions because they are more likely to affect cell function directly. The transcriptome is therefore an attractive subject of research in cancer and other human pathologies, and some of the cancer genes, such as FOXL2 in granulosa-cell tumors [9] and ARID1A in clear cell carcinomas of the ovary [10], were initially discovered through transcriptome sequencing.

The calling of variants with sequencing data from transcriptome (RNA-seq) is more challenging because of the splice junctions. Tools like RVBoost [11], SNPiR [12] or GATK Haplotypecaller are created to address this problem. Somatic variant calling from RNA is more difficult because of RNA processing like RNA-editing, allele-specific expression, variable levels of gene expression, and the heterogeneity of tumors which leads to low variant frequencies of some mutations [13]. Tools such as RVBoost, SNPiR, and GATK Haplotypecaller can be used to perform variant calling from RNA, but their performance and limitations for so-

*Correspondence: jchien@kumc.edu
[1]Department of Cancer Biology, University of Kansas Medical Center, 3901 Rainbow Blvd., 66160 Kansas City, KS, USA
Full list of author information is available at the end of the article

matic variant calling have not been studied previously. Nonetheless, these approaches have the potential to provide an orthogonal method to validate DNA sequence variations by complementing the analysis with RNA sequence analysis.

It should be noted that the integrated approach used by RADIA [14], that combines the variant sequence analysis from DNA and RNA sequencing, allows discovery of DNA sequence variations in expressed genes and better characterization of the effect of mutations on gene expression and phenotypic alterations, but its use of WES introduces cost. The limitation of RADIA is that it requires DNA sequencing data, and RNA sequence analysis was used just as a supplement. Moreover, DNA sequence variations are considered as the ground truth, and RNA variants not supported by DNA sequencing were rejected as false-positives. Although variants discovered only by RNA sequencing have the potential of being false-positives, some of these variants may represent missed calls from DNA sequencing or RNA-editing sites that have not been annotated. A detailed comparison of DNA and RNA variants from different tools will provide us with more precise processing and discovery of sequence variations from RNA and DNA sequencing.

In this study, we performed a detailed comparison of DNA and RNA sequence variations from 21 pairs of whole exome and mRNA sequencing from ovarian cancer genomes. We described variants discovered in RNA-seq through three publicly available tools, namely MuTect2, RVboost and SNPiR, and developed the best combination of these tools that enables discovery of variants from RNA sequence with high precision and sensitivity. We also show that most of the variants which would be classified as false-positives or false-negatives can be explained by biological characteristics.

DATA DESCRIPTION

Twenty one samples of ovarian serous cystadenocarcinoma from The Cancer Genome Atlas (TCGA) were divided into two groups: 11 cases that were sensitive to the cancer treatment and 10 cases that were resistant. Sensitive cases had a progression-free survival of more than 18 months, and resistant cases had progression-free survival of less than 12 months. The clinical data for the patients were retrieved from cBioPortal ([15–17]) and the Illumina sequence files for tumor RNA and normal blood DNA were retrieved from cghub [18] and gdc [19] (see Supplementary Table 3). Whole exome sequencing and mRNA sequencing datasets were available from each patient.

Additional data were provided by Dr. Andrea Mariani and came from 2 different tumor samples from a patient with serous ovarian carcinoma.

ANALYSIS

Performance characteristics of each method and different combinations of two or more methods

To describe the performance characteristics of each method, we performed variant calling using RVBoost, SNPiR, and MuTect2 separately. Each caller alone calls many variants which are not validated by DNA somatic variants (discordant calls), while SNPiR calls the most variants (see Figure 2(A)). Mutect2 provides the least amount of variant calls not supported by DNA sequencing compared to the other two methods. However, only 10% of variant calls made by Mutect2 was supported by DNA sequencing. These results indicate that any single caller is not adequate in discovering variants with high specificity. Therefore, we next tested if any combination of three calling methods will provide higher rate of variant calls supported by DNA sequencing. The combination of all three calling methods (Tier1) leads to 81.8% of variants which are validated by DNA somatic variants (concordant calls) (see Figure 2(B)). The combination of Tier1 with mutations called by Mutect2 and SNPiR (Tier2) leads to a higher sensitivity while the precision is still in a moderate range. For the following analysis, we concentrated only on Tier1.

Performance of a combined calling method

A total of 634 somatic mutations were called from 21 tumor samples. 518 mutations of them were concordant and 116 were discordant (see Table 1). On the DNA level, a total of 10099 mutations were called and 9864 of them were not called by our method.

Variants not found in RNA

To understand why variant calls from RNA sequencing missed a large majority of variant calls observed by DNA sequencing, we checked the properties of variants missed by RNA callers. From the 9864 missed somatic variants, 6949 (70.4%) were not in exonic regions (see Supplementary Figure 1). From the mutations in exonic regions, 2046 (20.9%) were missed because these variants are from genes with less abundant transcripts. The effect of transcript abundance on variants discovered from RNA-seq could also be observed in the percentage of concordant calls: 516 (15%) of the expressed mutations were called by Tier1 (see Figure 3 (A)) but when the expression is higher (DP>10) 34.6% of the somatic mutations were called. This confirms that an important factor in RNA-seq variant calling is the expression level.

Among the mutations found by DNA callers but missed by Tier1 from highly expressed genes, 594 (6.0%) of mutations in tumor DNA and 756 (7.7%) of mutations in tumor RNA had a variant fraction < 0.20

(see Figure 3 (B), Supplementary Figure 2). This result shows that one of the limitations of RNA-based variant calling methods is that it is highly dependent on the variant allele frequency. When the mutation has a low variant frequency at DNA level, it is possible to miss these mutations because of the heterogeneity of the tumor sample. From the variants with high expression and high variant allele frequency, thirty one mutations were not called by at least one of the callers. Ninety six mutations were filtered out by at least one of the callers because of potential evidence of germline variants or because the realigning step with PBLAT shows that these variants could come from mis-mapping. Most of the variants which are missed at a low variant frequency are called by MuTect2 or SNPiR alone (see Figure 3 (C)). By studying the filter steps in future work, we may be able to call a higher number of those variants.

Variants not found in DNA

The differences in coverage or allele fraction between DNA and RNA datasets could contribute to discordant calls. Therefore, we checked those attributes at discordant sites. Twenty four (20.7%) of the discordant mutations had a read depth (DP) of uniquely mapping reads under 10 at DNA level (see Sublementary Figure 3) and another 25 (21.6%) mutations had a variant allele frequency (VF) above zero at DNA level, indicating that these low level DNA variants were missed by DNA-based callers by TCGA. Twenty four variants with 0 variant fraction at DNA level but high DP in DNA normal, DNA tumor and RNA tumor were mostly either A>G or C>T (see Sublementary Figure 5). Those variants were found at 15 different positions, of which one variant (chr3:58141791 A>G [FLNB:p.M2324V]) is found in 4 different samples and another (chr20:10285837 C>T) in 9 different samples. These likely represent probably non-annotated RNA-editing sites [20–22].

Because we observed differences in the variant fraction at the discordant sites, we next expanded the analysis to all sites. Interestingly, we observed weak correlation of variant fraction between tumor DNA and tumor RNA at positions with DP>0 for tumor DNA and RNA (see Figure 4 (A)). When we limit the analysis to positions with DP>10 for tumor DNA (see Figure 4 (B)) or tumor and normal DNA (see Figure 4 (C)), we also observed a weak correlation. Finally, when we limit the analysis to positions with DP>10 for tumor DNA and RNA and normal DNA, we observed a strong correlation of variant fraction between RNA and DNA (see Figure 4 (D)). Only four mutations had variant frequencies around 50% at DNA level and 100% at RNA level which suggests that these are imprinted genes. These results suggest that variant fraction in abundant transcripts are strongly correlated with variant fractions at DNA level. Therefore, RNA variant fraction may be used as a substitute for DNA variant fraction for subclone phylogenetic analysis.

Detection of artificial spiked variants

To further assess the performance of RNA-based callers, we used BamSurgeon and spiked-in 200 artificial RNA sequence variants at varying variant fractions in two tumor transcriptomes. From the 200 simulated variant positions, 120 were actually spiked in because failed positions have too low read depth even if the positions for spiking were obtained from expressed genes. On average 71% of all spiked-in variants were found by each caller alone. The combination of all three callers leads to a calling of around 50% of all spiked-in mutations (see Table 2, Supplementary Figure 5). By using Tier2, we were able to call 60% of all spiked-in mutations. 55.6% of the mutations missed by Tier1 are not in coding regions (see Table 3). From the remaining missed variants, 15.7% have a variant allele fraction of less than 0.2 and 6.1% have high variant allele fraction but have a DP<10 in DNA.

Comparison between RADIA and VaDiR

Since RADIA performs function similar to our workflow VaDiR, we compared the performance differences between RADIA and VaDiR. RADIA uses DNA variant calling as the primary method and use RNA variant calling as a supplement. All somatic variants called by RADIA are supported by DNA-level evidence and RNA-only variants are not called by RADIA. Therefore, we limited our comparison to variants that are found at both RNA and DNA levels by RADIA and VaDiR. A total of 308 mutations were called by either RADIA or VaDiR or both in six samples. Of these, 175 mutations were called by both methods, 12 mutations were called by VaDiR only, and 121 mutations were called by RADIA only (see Supplementary Figure 6). From these 121 mutations, 40 (33.1%) had a read depth below 10 in RNA. 52 (43.0%) mutations, with a read depth over 10, had a variant fraction below 0.20. This shows again the limitation of method based only on RNA. Six of the remaining 29 variants were in non-exonic regions and would not be called by our method.

Ovarian cancer: resistant vs. sensitive

Since variant calling from RNA-seq provides both mutational status and gene expression, the number of mutations found by RNA-seq may be associated with pathologic or clinical phenotypes. In contrast, the total number of mutations found at the DNA level may

not be associated with pathologic or clinical phenotype because it may be confounded by potentially non-relevant mutations in non-coding region or in genes that are not expressed. To determine if variant calling from RNA-sequencing may provide novel insights into clinical phenotype, we characterized the number of mutations in expressed genes from RNA-seq obtained from 10 chemotherapy-resistant and 11 chemotherapy-sensitive ovarian carcinomas. We considered concordant mutations only (those found by both RNA- and DNA-based callers) for the analysis. The results indicate that concordant rate is higher for Tier1 mutations compared to Tier2 mutations although total number of mutations are higher in Tier2 (see Figure 5 (A)). We observed higher amount of mutations in chemotherapy-sensitive ovarian carcinomas compared to chemotherapy-resistant counterparts (see Figure 5 (A)). This result is consistent with previous studies indicating that sensitive tumor samples have a higher mutation rate in ovarian cancer [23]. In these samples, number of mutations at either DNA or RnA levels was significantly higher in sensitive carcinomas compared to resistant carcinoma samples (see Figure 5 (B)).

A higher mutation burden in sensitive tumors samples may be the result of high mutagenic processes in the cancer cells or the result of a high degree of intratumor heterogeneity or tumor subclones. If high levels of tumor subclones with clonal driver mutations are contributing to higher mutation burden in sensitive tumor samples, we expect these mutations will exist in lower variant fractions because they represent unique tumor subclones. Therefore, we limit our variant fraction analysis to variants that produce nonsynonymous mutations because they are more likely to contribute to a change in phenotype and the evolution of tumor subclones. Results, shown in supplementary Figure 7, indicate that differences in variant fractions between sensitive and resistant samples are not significant. Interestingly, sensitive samples have significantly lower variant allele fractions in non-COSMIC mutations compared to resistant samples both at the RNA (pValue=0.034) and DNA level (pValue=0.017)(see Supplementary Figure 7 (B)). These results suggest that these mutations are coming from subclonal populations. The higher levels of clonal heterogeneity and mutation burden in sensitive samples may be the result of defects in DNA repair, and this tumor cell characteristics may explain why they are sensitive to platinum-based chemotherapy.

DISCUSSION

With our approach, we were able to call variants with high precision. Only a small fraction of the variants which are called in RNA but not in DNA are likely false positives. The remaining discordant variants are either RNA-editing sites or are missed at the DNA level as well. Most of the variants called in DNA but missed by VaDiR are not in coding regions or are not expressed. We also missed many variants that have low variant frequency. Those are called by none of the callers, MuTect2 only, or SNPiR only. These mutations are observed at low variant frequencies in tumor DNA, and therefore they likely represent mutations from small subsets of tumor subclones. Finally, our approach missed approximately 15% of variants (127/853) with a high DP and a high variant allele frequency. Among those 127, 96 mutations were called by at least one method, indicating that consensus calling is too stringent or that parameters for one of the callers is not optimal. Those data are confirmed by the artificial spiked-in variants where only variants with high variant frequency could be called by all three callers.

The comparison to RADIA shows that we are missing mainly variants in low frequency ranges while RADIA is missing a few variants with high variant frequency in RNA. This confirms the limitation of calling variants only from RNA, but also shows that we are able to call a great number of somatic variants without the need for whole exome sequencing. We were also able to find new possible RNA-editing sites, which should be investigated in future studies. Therefore, our workflow provides new capabilities that are missing in existing approaches and can be used to gain novel insight into disease phenotype.

Our main concern in future studies would be to increase the number of concordant variant calls by adjustment of the filtering steps from SNPiR and RV-boost, and to investigate the reasons for the missed somatic variants with high variant allele frequency.

METHODS

Software

To process the data, we used the software STAR, BWA MEM, Genome Analysis Toolkit (GATK), SNPiR, RV-boost, R, Picard, BEDtools, ANNOVAR, SAMtools, and BCFtools which is a part of the SAMtools package [2, 11, 12, 24–31] (see Supplementary Table 1). To analyze our results, we used the software BAMSurgeon, R, and RADIA [14, 32]. We used reference files from Broad Institute's resource bundle [33], including the UCSC hg19 (GRCh37) reference genome, known indels from the 1000 Genomes Project, and known SNPs from dbSNP.

To validate the results that we obtained from RNA, we used somatic variants from DNA called by any two of the variant callers MuSE, MuTect2, SomaticSniper, and VarScan. We retrieved the corresponding VCF files from GDC [19].

We implemented SNPiR with the following modifications: In the file BLAT_candidates.pl at line 94, the developers incorrectly handled the information in the CIGAR-string of hardclipped reads, that resulted in a faulty shift of the read position. We corrected the code to handle CIGAR-strings correctly. This modification was necessary because our workflow differs from the SNPiR workflow in that we use hard-clipped reads. At the same location, we also added an optimization to avoid searching through more base positions than necessary. Further, we changed the filter to use PBLAT instead of BLAT, so we could utilize additional CPU threads to improve execution time. We made similar changes in the file filter_mismatch_first6bp.pl at line 84. In addition, we optimized the search algorithm in filter_intron_near_splicejuncts.pl by skipping exons and genes that do not contain a given variant position (which also introduced the requirement that SNPiR's gene annotation table be sorted by position) and moderately improve code for readability. Finally, we modified convertVCF.sh to filter out any variant whose read depth (DP) value was zero, in order to prevent division-by-zero errors that occurred with our dataset. Rather than replacing the original SNPiR files in our distribution, we have included both versions and prefixed our file names with "revised_".

For comparison with our method, we implemented RADIA with the following modification: During BLAT filtering, RADIA also incorrectly handled the hard-clipped reads. We corrected the code for the same reasons as described for the SNPiR implementation.

For creation of the figures, the R package ggplot2 [34] was used.

Aligning sequences

The procedure for the alignment to the reference genome followed GATK Best Practices [35, 36]. For RNA-seq, we used the STAR aligner in 2-pass mode with the parameters implemented by ENCODE project. The resulting aligned reads were processed to add read groups, sort, mark duplicates, split reads that spanned splice junctions, create an index, realign around known indels, reassign mapping qualities, and recalibrate base quality scores.

For DNA, we used the BWA MEM aligner with the same reference genome. The resulting aligned reads were processed to add read groups, sort, mark duplicates, create an index, realign around known indels, reassign mapping qualities, and recalibrate base quality scores.

Calling variants

A refined BAM file for each sample is then used to process the variant calling. Three different methods for calling are used: RVboost, SNPiR, and MuTect2. The first two methods are for germline variants in RNA and the last method is for somatic variants in DNA. None of these methods is for somatic variant calling in RNA. RVboost and SNPiR use the same variant caller, UnifiedGenotyper from GATK, but different filtering procedures. RVboost filters variants using a statistical learning method called boosting, whereas SNPiR uses hard filtering in 7 steps (see Supplementary Table 2). To adapt MuTect2's results for RNA, we implemented three of SNPiR's hard-filtering steps. RVboost and SNPiR only need the refined RNA BAM file from the tumor tissue. MuTect2 needs both the refined RNA BAM from the tumor tissue and the refined DNA BAM from normal tissue.

Filtering somatic variants by caller intersection and additional hard filters

In addition to the filtering procedures of the variant callers themselves, we further filtered our results by taking an intersection of vcf files from the three callers. We restricted our final, combined callset to the variants called by all three methods (Tier 1) or supplemented by variants called by MuTect2 and SNPiR (Tier2). We also applied our own hard filters, only accepting variants with a read depth (DP) of at least five and a variant allele frequency (VF) of less than 3% in uniquely mapping reads (Mapping quality of at least 40) in the normal DNA at the corresponding position.

Processing artificial spiked variants

We used BAMSURGEON to spike in 200 variants in coding regions of two ovarian tumor samples, such that each sample had a different random frequency of spiked-in variants. The samples were then processed by VaDiR.

Processing samples with RADIA

Six samples from TCGA, three from resistant patients and three from sensitive patients, were processed with RADIA. This analysis required three BAM files from each sample: one from normal blood DNA, one from tumor DNA, and one from tumor RNA. We followed the instructions provided by RADIA for filtering. We used all possible filters provided by RADIA.

AVAILABILITY AND REQUIREMENTS

- Project name: somatic VaDiR
- Project home page: e.g. http://to.be.added.later
- Operating system(s): Linux/Unix 64-Bit
- Programming language: Perl, R, Java, Shell
- Other requirements: Java 7 and 8, R 3.3 or higher
- License: MIT
- Any restrictions to use by non-academics: no

AVAILABILITY OF SUPPORTING DATA AND MATERIALS

The data sets supporting the results of this article are available in the open science framework repository, [37], and the GDC repository, [19].

List of abbrevations

- WGS: Whole genome sequencing
- WES: Whole exome sequencing
- RNA-seq: Data from sequencing cDNA derived from RNA
- Tier1: Variants called by each caller (SNPiR, RVBoost, MuTect2)
- Tier2: Variants called by Tier1 and variants called by SNPiR and MuTect2.
- VF: Variant fraction
- DP: read depth

Ethics approved and consent to participate

The datasets were obtained from the Cancer Genome Atlas, and the use of data was approved under the Project #4017 at dbGaP.

Consent for publication

Not applicable

Competing interests

The authors declare that they have no competing interests.

Funding

The study is funded by the University of Kansas Endowment Association, the University of Kansas Cancer Center Support Grant (P30-CA168524), the Cancer Center Cancer Biology program, and the Department of Defense Ovarian Cancer Research Program under award number (W81XWH-10-1-0386). Views and opinions of, and endorsements by the author(s) do not reflect those of the US Army or the Department of Defense.

Author's contributions

- Development of workflow: Jeremy Chien and Lisa Neums
- Conception and design: Jeremy Chien and Lisa Neums
- Acquisition of data: Dr. Andrea Mariani
- Analysis and interpretation of data: Lisa Neums, Jeremy Chien and Seiji Suenaga
- Writing, review, and revision of the manuscript: Jeremy Chien, Lisa Neums and Seiji Suenaga
- Administration, technical, or material support: Jeremy Chien and Peter Beyerlein

Acknowledgements

The results published here are in whole or part based upon data generated by the TCGA Research Network: http://cancergenome.nih.gov/. We acknowledge Dr. Devin Koestler for helpful discussions and comments.

Author details

[1]Department of Cancer Biology, University of Kansas Medical Center, 3901 Rainbow Blvd., 66160 Kansas City, KS, USA. [2]Department of Bioinformatics and Biosystems Technology, University of Applied Sciences Wildau, Hochschulring 1, 15745 Wildau, Germany. [3]Obstetrics and Gynecology, Cancer Center, Mayo Clinic, 200 First St. SW, 55905 Rochester, MN, USA.

References

1. The Cost of Sequencing a Human Genome. https://www.genome.gov/27565109/the-cost-of-sequencing-a-human-genome/
2. McKenna, A., Hanna, M., Banks, E., Sivachenko, A., Cibulskis, K., Kernytsky, A., Garimella, K., Altshuler, D., Gabriel, S., Daly, M., DePristo, M.A.: The genome analysis toolkit: a mapreduce framework for analyzing next-generation dna sequencing data. Genome Research **20**, 1297–1303 (2010)
3. Fan, Y., Xi, L., Hughes, D.S., Zhang, J., Zhang, J., Futreal, P.A., Wheeler, D.A., Wang, W.: Muse: accounting for tumor heterogeneity using a sample-specific error model improves sensitivity and specificity in mutation calling from sequencing data. Genome Biol. **17**(1), 178 (2016)
4. Larson, D.E., Harris, C.C., Chen, K., Koboldt, D.C., Abbott, T.E., Dooling, D.J., Ley, T.J., Mardis, E.R., Wilson, R.K., Ding, L.: Somaticsniper: identification of somatic point mutations in whole genome sequencing data. Bioinformatics **28**(3), 311–317 (2012)
5. Koboldt, D.C., Zhang, Q., Larson, D.E., Shen, D., McLellan, M.D., Lin, L., Miller, C.A., Mardis, E.R., Ding, L., Wilson, R.K.: Varscan 2: somatic mutation and copy number alteration discovery in cancer by exome sequencing. Genome Research **22**(3), 568–576 (2012)
6. Guettouche, T., Zuchner, S.: Improved coverage and accuracy with strand-conserving sequence enrichment. Genome Med **5**(5), 46 (2013)
7. Parla, J.S., Iossifov, I., Grabill, I., Spector, M.S., Kramer, M., McCombie, W.R.: A comparative analysis of exome capture. Genome Biol **12**(9), 97 (2011)
8. Garcia-Ortega, L.F., Martinez, O.: How many genes are expressed in a transcriptome? estimation and results for rna-seq. PLoS One **10**(6), 0130262 (2015)
9. Shah, S.P., Kobel, M., Senz, J., Morin, R.D., Clarke, B.A., Wiegand, K.C., Leung, G., Zayed, A., Mehl, E., Kalloger, S.E., Sun, M., Giuliany, R., Yorida, E., Jones, S., Varhol, R., Swenerton, K.D., Miller, D., Clement, P.B., Crane, C., Madore, J., Provencher, D., Leung, P., DeFazio, A., Khattra, J., Turashvili, G., Zhao, Y., Zeng, T., Glover, J.N., Vanderhyden, B., Zhao, C., Parkinson, C.A., Jimenez-Linan, M., Bowtell, D.D., Mes-Masson, A.M., Brenton, J.D., Aparicio, S.A., Boyd, N., Hirst, M., Gilks, C.B., Marra, M., Huntsman, D.G.: Mutation of foxl2 in granulosa-cell tumors of the ovary. N Engl J Med **360**(26), 2719–29 (2009)
10. Wiegand, K.C., Shah, S.P., Al-Agha, O.M., Zhao, Y., Tse, K., Zeng, T., Senz, J., McConechy, M.K., Anglesio, M.S., Kalloger, S.E., Yang, W., Heravi-Moussavi, A., Giuliany, R., Chow, C., Fee, J., Zayed, A., Prentice, L., Melnyk, N., Turashvili, G., Delaney, A.D., Madore, J., Yip, S., McPherson, A.W., Ha, G., Bell, L., Fereday, S., Tam, A., Galletta, L., Tonin, P.N., Provencher, D., Miller, D., Jones, S.J., Moore, R.A., Morin, G.B., Oloumi, A., Boyd, N., Aparicio, S.A., Shih Ie, M., Mes-Masson, A.M., Bowtell, D.D., Hirst, M., Gilks, B., Marra, M.A., Huntsman, D.G.: Arid1a mutations in endometriosis-associated ovarian carcinomas. N Engl J Med **363**(16), 1532–43 (2010)
11. Wang, C., Davila, J.I., Baheti, S., Bhagwate, A.V., Wang, X., Kocher, J.P., Slager, S.L., Feldman, A.L., Novak, A.J., Cerhan, J.R., Thompson, E.A., Asmann, Y.W.: Rvboost: Rna-seq variants prioritization using a boosting method. Bioinformatics **30**(23), 3414–3416 (2014)
12. Piskol, R., Ramaswami, G., Li, J.B.: Reliable identification of genomic variants from rna-seq data. Am J Hum Genet **93**(4), 641–651 (2013)
13. Spence, J.M., Spence, J.P., Abumoussa, A., Burack, W.R.: Ultradeep analysis of tumor heterogeneity in regions of somatic hypermutation. Genome Med **7**(1), 24 (2015)
14. Radenbaugh, A.J., Ma, S., Ewing, A., Stuart, J.M., Collisson, E.A., Zhu, J., Haussler, D.: Radia: Rna and dna integrated analysis for somatic mutation detection. PLoS One **9**(11) (2014)
15. cBioPortal for Cancer Genomics. http://www.cbioportal.org/
16. Gao, J., Aksoy, B.A., Dogrusoz, U., Dresdner, G., Gross, B., Sumer, S.O., Sun, Y., Jacobsen, A., Sinha, R., Larsson, E., Cerami, E., Sander, C., Schultz, N.: Integrative analysis of complex cancer genomics and clinical profiles using the cbioportal. Sci Signal **6**(269) (2013). pl1
17. Cerami, E., Gao, J., Dogrusoz, U., Gross, B.E., O, S.S., A, A.B., Jacobsen, A., Byrne, C.J., Heuer, M.L., Larsson, E., Antipin, Y., B, R., Goldberg, A.P., Sander, C., Schultz, N.: The cbio cancer genomics portal: An open platform for exploring multidimensional cancer genomics data. Cancer Discovery **2**(5), 401–404 (2012)
18. Cancer Genomics Hub. https://cghub.ucsc.edu/
19. GDC Data Portal - National Institutes of Health. https://gdc-portal.nci.nih.gov/
20. Wang, I.X., So, E., Devlin, J.L., Zhao, Y., Wu, M., Cheung, V.G.: Adar regulates rna editing, transcript stability, and gene expression. Cell Rep. **5**(3), 849–860 (2013)
21. Blanc, V., Davidson, N.O.: Apobec-1 mediated rna editing. Wiley Interdiscip Rev Syst Biol Med. **2**(5), 594–602 (2011)
22. Blanc, V., Park, E., Schaefer, S., Miller, M., Lin, Y., Kennedy, S., Billing, A.M., Ben Hamidane, H., Graumann, J., Mortazavi, A., Nadeau, J.H., Davidson, N.O.: Genome-wide identification and

functional analysis of apobec-1-mediated c-to-u rna editing in mouse small intestine and liver. Genome Biol **15**(6), 79 (2014)
23. Birkbak, N.J., Kochupurakkal, B., Izarzugaza, J.M.G., Eklund, A.C., Y, L., Liu, J., Szallasi, Z., Matulonis, U.A., Richardson, A.L., Iglehart, J.D., Wang, Z.C.: Tumor mutation burden forecasts outcome in ovarian cancer with brca1 or brca2 mutations. PLoS ONE (2013)
24. Wang, K., Li, M., Hakonarson, H.: Annovar: functional annotation of genetic variants from high-throughput sequencing data. Nucleic Acids Res **38**(16), 164 (2010)
25. Dobin, A., Davis, C.A., Schlesinger, F., Drenkow, J., Zaleski, C., Jha, S., Batut, P., Chaisson, M., Gingeras, T.R.: Star: ultrafast universal rna-seq aligner. Bioinformatics **29**(1), 15–21 (2013)
26. Li, H., Durbin, R.: Fast and accurate long-read alignment with burrows-wheeler transform. Bioinformatics **26**(5), 589–95 (2010)
27. Picard. http://broadinstitute.github.io/picard
28. Li, H.: A statistical framework for snp calling, mutation discovery, association mapping and population genetical parameter estimation from sequencing data. Bioinformatics **27**(21), 2987–93 (2011)
29. Li, H., Handsaker, B., Wysoker, A., Fennell, T., Ruan, J., Homer, N., Marth, G., Abecasis, G., Durbin, R., Genome Project Data Processing, S.: The sequence alignment/map format and samtools. Bioinformatics **25**(16), 2078–9 (2009)
30. Team, R.D.C.: R: A Language and Environment for Statistical Computing. R Foundation for Statistical Computing, Vienna, Austria (2008). R Foundation for Statistical Computing. http://www.R-project.org
31. Quinlan, A.R., Hall, I.M.: Bedtools: a flexible suite of utilities for comparing genomic features. Bioinformatics **26**(6), 841–2 (2010)
32. Ewing, A.D., Houlahan, K.E., Hu, Y., Ellrott, K., Caloian, C., Yamaguchi, T.N., Bare, J.C., P'ng, C., Waggott, D., Sabelnykova, V.Y., participants, I.-T.D.S.M.C.C., Kellen, M.R., Norman, T.C., Haussler, D., Friend, S.H., Stolovitzky, G., Margolin, A.A., Stuart, J.M., Boutros, P.C.: Combining tumor genome simulation with crowdsourcing to benchmark somatic single-nucleotide-variant detection. Nat Methods **12**(7), 623–30 (2015)
33. Broad Institute's Resource Bundle. ftp://ftp.broadinstitute.org/bundle/2.8/hg19/
34. Wickham, H.: Ggplot2: Elegant Graphics for Data Analysis. Springer, ??? (2009). http://ggplot2.org
35. DePristo, M., Banks, E., Poplin, R., Garimella, K., Maguire, J., Hartl, C., Philippakis, A., del Angel, G., Rivas, M.A., Hanna, M., McKenna, A., Fennell, T., Kernytsky, A., Sivachenko, A., Cibulskis, K., Gabriel, S., Altshuler, D., Daly, M.: A framework for variation discovery and genotyping using next-generation dna sequencing data. NATURE GENETICS **43**, 491–498 (2011)
36. Van der Auwera, G.A., Carneiro, M., Hartl, C., Poplin, R., del Angel, G., Levy-Moonshine, A., Jordan, T., Shakir, K., Roazen, D., Thibault, J., Banks, E., Garimella, K., Altshuler, D., Gabriel, S., DePristo, M.: From fastq data to high-confidence variant calls: The genome analysis toolkit best practices pipeline. CURRENT PROTOCOLS IN BIOINFORMATICS **43**, 11101–111033 (2013)
37. Open Science Framework Repository for VaDiR Data. http://www.osf.io/ap5b7

Illustrations and figures

Figure 1 Workflow of processing the variant call for somatic variants with RNA-seq. Aligning is done by STAR and BWA MEM and the refined mapping follows GATK Best Practices. The variant calling is done by Unified Genotyper (GATK) and MuTect2 (GATK). The following filtering steps are done by RVBoost and SNPiR.

Figure 2 Intersection of the three variant calling methods. (A) Intersection of the three methods with all somatic calls. The red arrows symbolize the amount of concordant calls. **(B)** Intersection of three methods with only concordant calls.

Figure 3 Variants called in tumor DNA. (A) Percentage of concordant calls of all somatic variants from expressed genes for each sample. With a high expression a higher percentage of concordant calls can be achieved. **(B)** Variant frequency in tumor DNA and RNA and normal DNA of all somatic variant called in DNA with a high expression (DP>10). **(C)** Comparison of somatic variants called in DNA which have high coverage in tumor DNA and RNA and normal DNA with variant calls from the three RNA callers. The names in the chart are the first letters of the caller SNPiR (s), RVBoost (r) and MuTect (m) or their combinations.

Figure 4 Correlation between the variant frequency of RNA and DNA. The four charts show different filtering steps of the read depth of tumor DNA and RNA and normal DNA.

Figure 5 Comparison of sensitive and resistant samples. (A) Test for significance of the difference in the variant allele frequency of nonsynonymous variants with DP>10 in DNA normal, DNA tumor and RNA tumor at tumor DNA and RNA level. **(B)** Comparison of variant frequency in DNA and RNA for each somatic variant.

Tables and captions

Table 1 Performance characteristics of VaDiR with the combination Tier1.

	DNA positive	DNA negative
RNA positive	518	116
RNA negative	9864	1595677

Table 2 Called spiked-in variants.

Sample	Tier1	Tier2
OV10	68 (52.71%)	78 (62.40%)
OV11	61 (52.59%)	68 (58.62%)
OV12	48 (48.74%)	69 (57.98%)

Table 3 Characteristics of missed spiked-in variants.

Tier1	OV10	OV11	OV12
all	105	96	99
missed	37	36	42
missed in coding region	16	17	18
missed in coding region by RNA VF>20%	11	9	13
missed in coding region by RNA VF>20% and normal DNA DP>10	8	7	11
**Tier2**	**OV10**	**OV11**	**OV12**
all	105	96	99
missed	27	29	31
missed in coding region	9	11	12
missed in coding region by RNA VF>20%	6	5	9
missed in coding region by RNA VF>20% and normal DNA DP>10	4	4	8

Figure

Click here to download Figure Figure1_Calling_methology_VaDiR.pdf

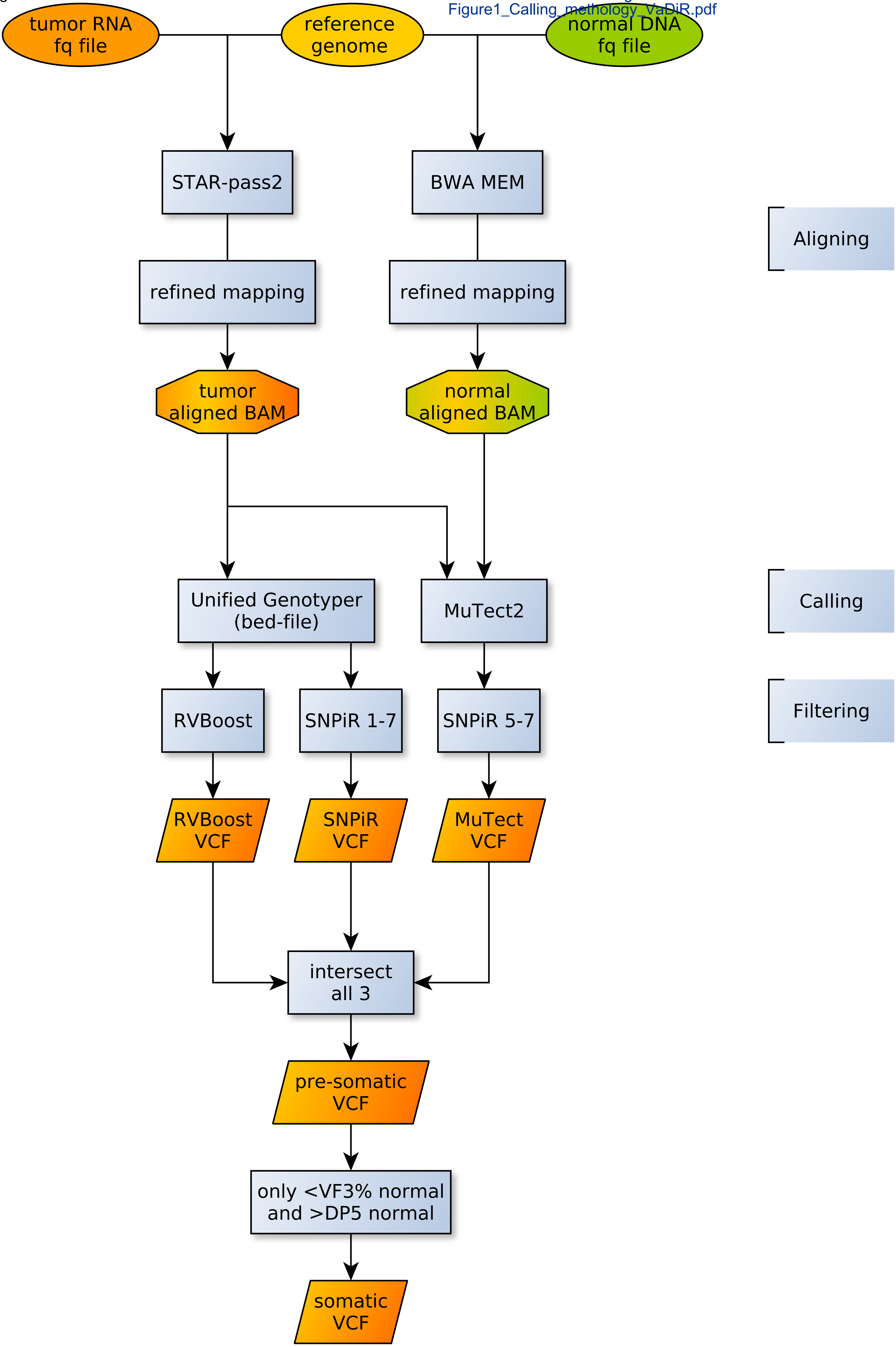

Figure

Click here to download Figure Figure2_all_srm_somatic.pdf

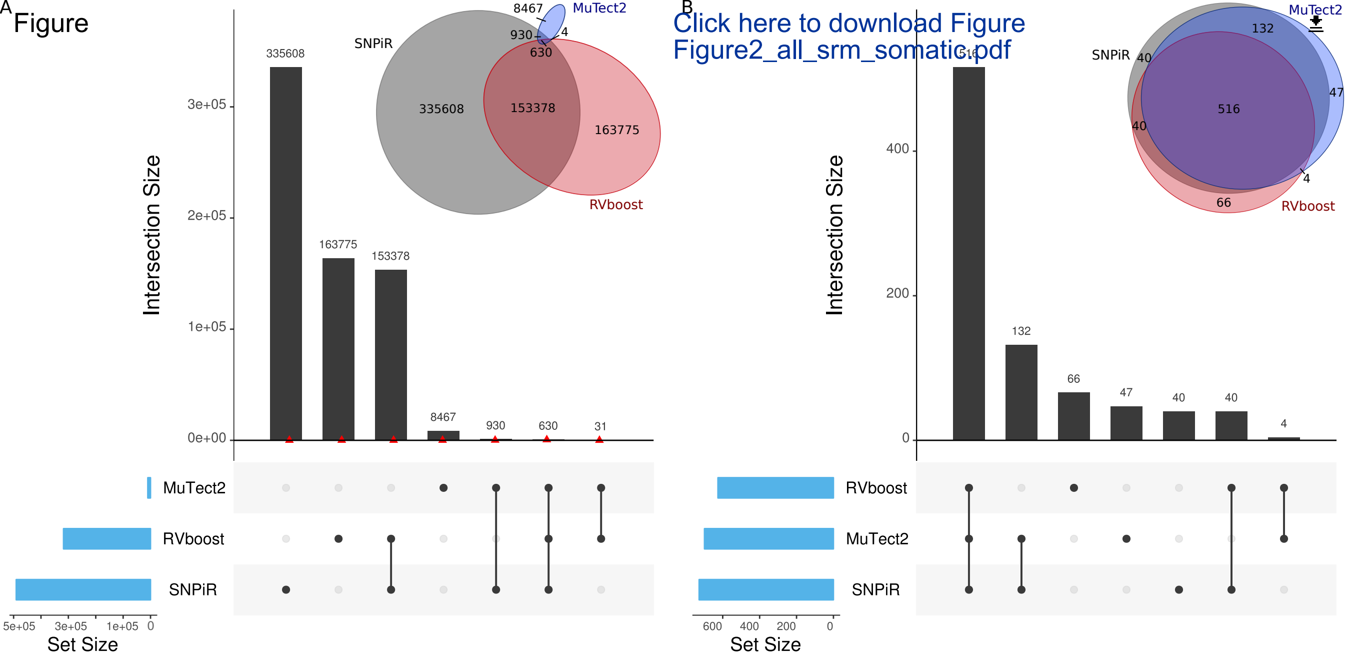

Figure

Click here to download Figure Figure3_false_negative_dp5af3c50_Pvalidation.pdf

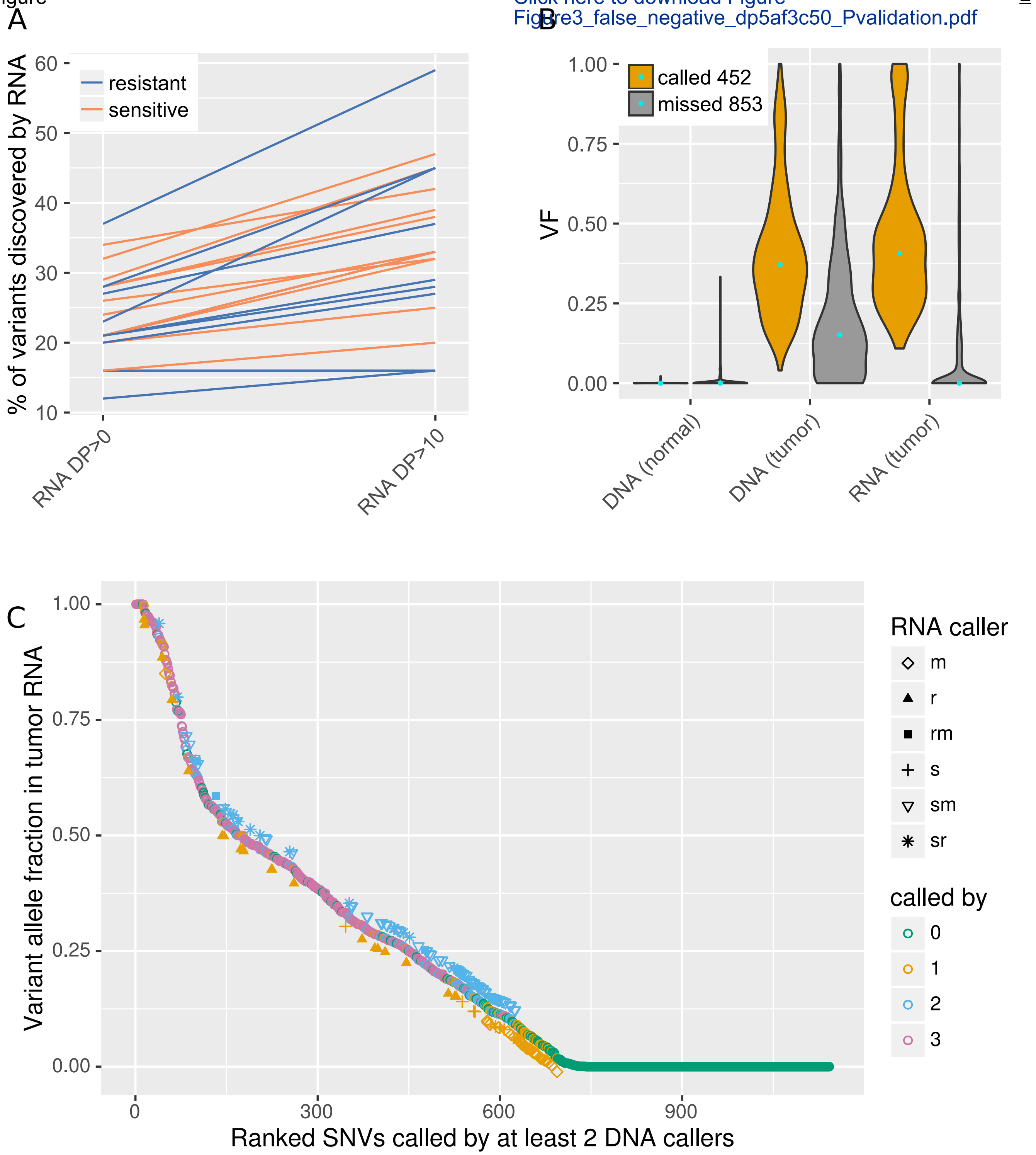

Figure

Click here to download Figure Figure4_all_VF_correlation_allDP_c50.pdf

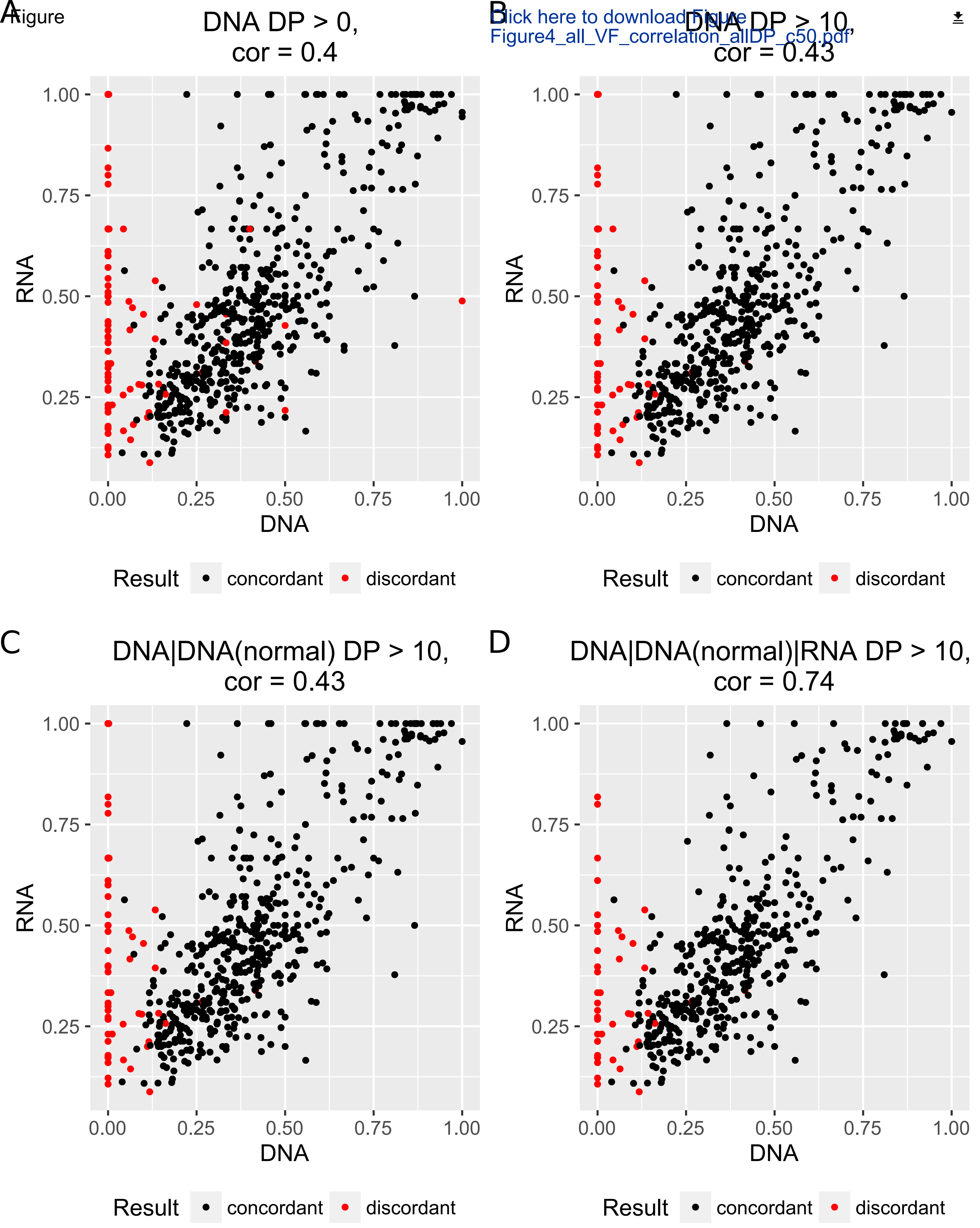

Figure

Click here to download Figure Figure5_numbers-resistantVSsensitive_c50.pdf

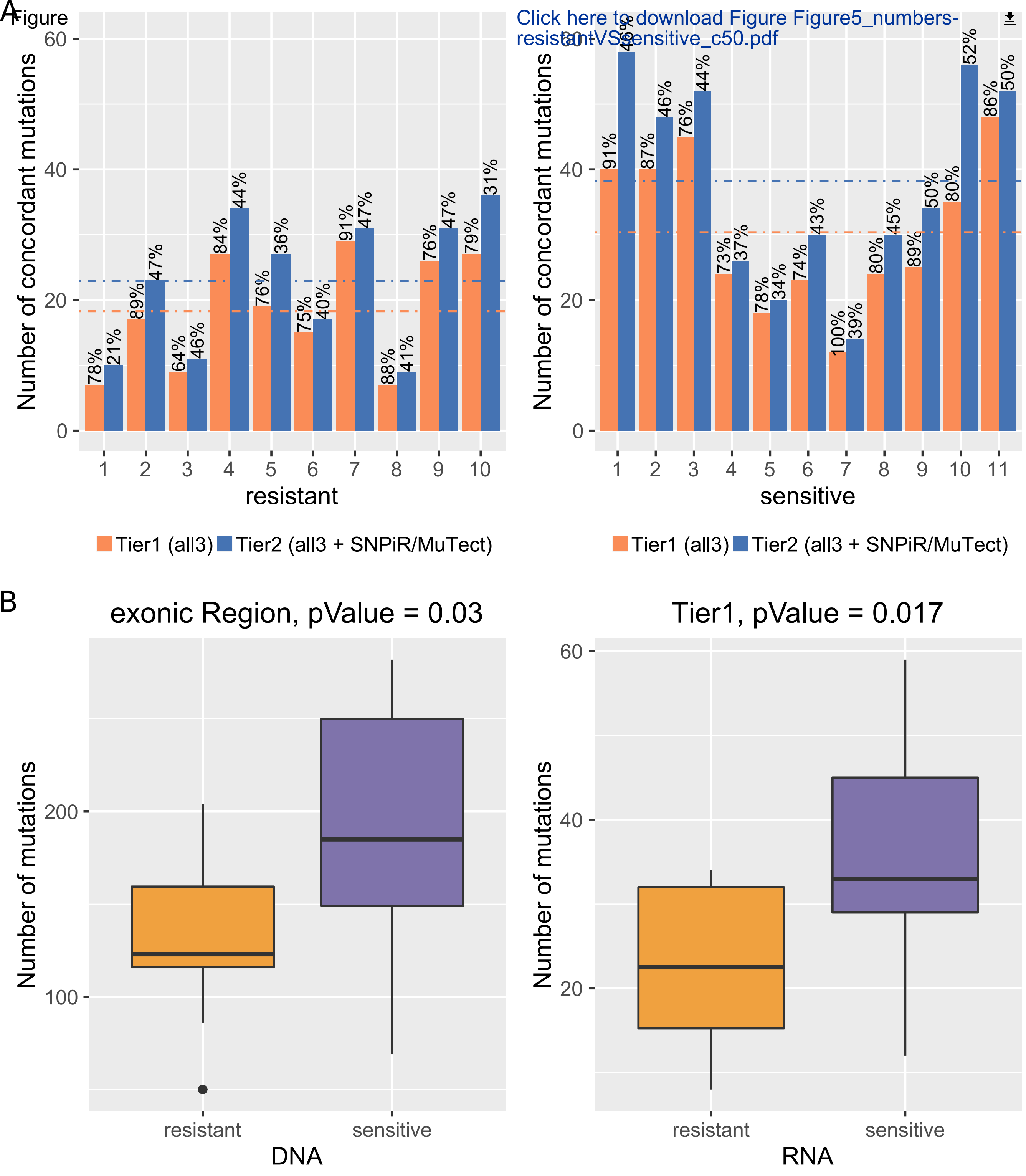

Supplementary Figure

Click here to access/download
Supplementary Material
SupplementaryFigure1_missed_timeline.pdf

Supplementary Figure

Click here to access/download

Supplementary Material

SupplementaryFigure2_false_negative_dp5af3c50_missed_pie.pdf

Supplementary Figure

Click here to access/download
Supplementary Material
SupplementaryFigure3_discordant_timeline.pdf

Supplementary Figure

Click here to access/download
Supplementary Material
SupplementaryFigure4_kindMutation_bargraph.pdf

Supplementary Figure

Click here to access/download
Supplementary Material
SupplementaryFigure5_spiked_violin.pdf

Supplementary Figure

Click here to access/download
Supplementary Material
SupplementaryFigure6_radia-violin.pdf

Supplementary Figure

Click here to access/download
Supplementary Material
SupplementaryFigure7_AF-resistantVSsensitive.pdf

Supplementary Table

Click here to access/download
Supplementary Material
SupplementaryTable1_used_Software.pdf

Supplementary Table

Click here to access/download
Supplementary Material
SupplementaryTable2_filtering_of_caller.pdf

Click here to access/download
Supplementary Material
SupplementaryTable3_Sample_ID_List.xls

THE UNIVERSITY OF KANSAS
Medical Center

December 6, 2016

Laurie Goodman, PhD
Editor in Chief
GigaScience

Dear Dr. Goodman,

I would like to submit the manuscript entitled, "*VADiR* – an integrated approach to Variant Detection in RNA" for consideration as a **Research** article in the journal *GigaScience*.

Although most of the available tools (such as SNPiR, RVboost) were developed for variant calling from RNA sequencing datasets, the performance of these methods for somatic variant calling from RNA sequencing was not previously evaluated.

In this study, we developed an approach that integrate two available RNA variant callers (RVboost and SNPiR) and also adapted MuTect2 to perform the analysis of variants in RNA datasets. We analyzed performance of each caller against the ground-truth DNA variants called by TCGA. These analyses showed the limitation of each caller, and therefore we developed an approach that uses the consensus calls from all three callers. This consensus calling approach produced variant calls with high precision. Finally, we packaged these tools into one integrated tool set to perform variant calling from RNA sequencing.

The main points in this study are:

- We implemented a software pipeline to process RNA-seq for a 2-pass alignment with STAR and GATK Best Practice. BWA-MEM and GATK Best Practice was used for the DNA-seq.
- We implemented a software pipeline that integrate RVBoost, SNPiR, and Mutect2 to perform consensus variant calling from RNA-seq.
- The software utilizes several established filters to remove known RNA sequence artifacts and improved specific steps in SNPiR for more efficient detection of variants.
- The performance of the proposed tool was evaluated by using two sets of data: (1) TCGA ovarian cancer data sets that contains validated DNA sequence variants; and (2) Three RNA-sequencing datasets with artificial variants spiked-in by BAMSurgeon.
- Application of our tool resulted in the identification of RNA-editing sites that are previously undocumented in the literature.
- The developed tool also provide evidence that mutation burden established from RNA-sequencing datasets is associated with clinical behavior.

Since our tool is a complete, standalone workflow, it can be easily integrated into established workflows or custom pipelines. We are confident that our tool will be of value to researchers interested in discovering somatic mutations from RNA-seq or those interested in using RNA-seq as an orthogonal validation platform for confirmation of DNA sequence variations.

Applied Genomics and Cancer Therapeutics (AGCT) Program, Department of Cancer Biology
Mail-Stop 1027 | 3901 Rainbow Blvd. | Kansas City, KS 66160 | (913) 945-8082 | Fax (913) 945-6650 | www.kumc.edu

Best regards,

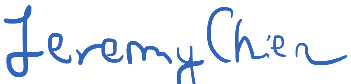

Jeremy Chien, PhD
Assistant Professor
University of Kansas Medical Center
Kansas City, KS 66160

This manuscript has been seen and approved by all listed authors.

Referees
Jean-Pierre Kocher - Kocher.JeanPierre@mayo.edu; Yan Asmann - asmann.yan@mayo.edu; Jian Ma, - jianma@cs.cmu.edu; Jin Billy Li - jin.billy.li@stanford.edu

Data access
http://www.osf.io/ap5b7
https://gdc-portal.nci.nih.gov

Tool: ***VADiR***
It will be available through GigaScience.

Applied Genomics and Cancer Therapeutics (AGCT) Program, Department of Cancer Biology
Mail-Stop 1027 | 3901 Rainbow Blvd. | Kansas City, KS 66160 | (913) 945-8082 | Fax (913) 945-6650 | www.kumc.edu
