# Supplementary material for: VaDiR: an integrated approach to Variant Detection in RNA
Source: Gigascience. 2017 Dec 18;7(2):1–13. doi: 10.1093/gigascience/gix122 (PMC5827345; doi:10.1093/gigascience/gix122)
Supplement: GIGA-D-16-00160_Revision_3.pdf [file gix122_giga-d-16-00160_revision_3.pdf]

GigaScience

VaDiR: an integrated approach to Variant Detection in RNA

--Manuscript Draft--

**Manuscript Number:**	GIGA-D-16-00160R3
**Full Title:**	VaDiR: an integrated approach to Variant Detection in RNA
**Article Type:**	Technical Note
**Funding Information:**	National Cancer Institute (P30-CA168524) — Not applicable<br>Department of Defense Ovarian Cancer Research Program (W81XWH-10-1-0386) — Dr. Jeremy Chien
**Abstract:**	Background<br>Advances in next-generation DNA sequencing technologies are now enabling detailed characterization of sequence variations in cancer genomes. With whole genome sequencing, variations in coding and non-coding sequences can be discovered. But the cost associated with it is currently limiting its general use in research. Whole exome sequencing is used to characterize sequence variations in coding regions, but the cost associated with capture reagents and biases in capture rate limit its full use in research. Additional limitations include uncertainty in assigning the functional significance of the mutations when these mutations are observed in the non-coding region or in genes that are not expressed in cancer tissue.<br><br>Results<br>We investigated the feasibility of uncovering mutations from expressed genes using RNA sequencing datasets with a method called "VaDiR: Variant Detection in RNA" that integrate three variant callers, namely: SNPiR, RVBoost and MuTect2. The combination of all three methods, which we called Tier1 variants, produced the highest precision with true positive mutations from RNA-seq that could be validated at the DNA level. We also found that the integration of Tier1 variants with those called by MuTect2 and SNPiR produced the highest recall with acceptable precision. Finally, we observed higher rate of mutation discovery in genes that are expressed at higher levels.<br><br>Conclusions<br>Our method, VaDiR, provides a possibility of uncovering mutations from RNA sequencing datasets that could be useful in further functional analysis. In addition, our approach allows orthogonal validation of DNA-based mutation discovery by providing complementary sequence variation analysis from paired RNA/DNA sequencing data sets.
**Corresponding Author:**	Jeremy Chien, PhD<br>University of New Mexico Health Sciences Center<br>Albuquerque, NM UNITED STATES
**Corresponding Author Secondary Information:**	
**Corresponding Author's Institution:**	University of New Mexico Health Sciences Center
**Corresponding Author's Secondary Institution:**	
**First Author:**	Lisa Neums
**First Author Secondary Information:**	
**Order of Authors:**	Lisa Neums<br>Seiji Suenaga<br>Peter Beyerlein<br>Devin Koestler, PhD<br>Sara Anders, B.S.

Powered by Editorial Manager® and ProduXion Manager® from Aries Systems Corporation

	Andrea Mariani
	Jeremy Chien
**Order of Authors Secondary Information:**	
**Response to Reviewers:**	Dear Dr. Zauner,<br><br>Thank you for your suggestions on the revision of our manuscript, titled “VaDiR: an integrated approach to Variant Detection in RNA”<br><br>Per your suggestion, we revised the manuscript to include the following:<br><br>A reference to the GigaDB entry with DOI.<br>[43] Neums L, Suenaga S, Beyerlein P, Koestler D, Anders S, Mariani A, Chien J. Supporting software for "VaDiR: an integrated approach to Variant Detection in RNA". 2017. http://dx.doi.org/10.5524/100360<br><br>We included the following statement in the “Availability of Supporting Data”:<br>"Supporting data and an archival copy of the code are also available via the GigaScience repository GigaDB [43].<br>For testing purposes we utilised data kindly provided by Dr. Andrea Mariani of Mayo Clinic, Rochester, Minnesota. Due to ethical constraints these data cannot be shared publicly, but if researchers would like to request access to these data please contact Dr. Andrea Mariani (mariani.andrea@mayo.edu) with a short description of why you require access and how you would use the data."<br><br>We updated the manuscript to include the project URL and RRID.<br><br>We also included information about third party code in supplemental table 3 (including citation and licence info).<br><br><br>Best regards,<br>Jeremy Chien
**Additional Information:**	
**Question**	**Response**
Are you submitting this manuscript to a special series or article collection?	No
**Experimental design and statistics**<br><br><br>Full details of the experimental design and statistical methods used should be given in the Methods section, as detailed in our Minimum Standards Reporting Checklist. Information essential to interpreting the data presented should be made available in the figure legends.<br><br><br>Have you included all the information requested in your manuscript?	Yes
**Resources**<br><br><br>A description of all resources used, including antibodies, cell lines, animals and software tools, with enough information to allow them to be uniquely	Yes

identified, should be included in the Methods section. Authors are strongly encouraged to cite Research Resource Identifiers (RRIDs) for antibodies, model organisms and tools, where possible.<br><br>Have you included the information requested as detailed in our Minimum Standards Reporting Checklist?	
**Availability of data and materials**<br><br>All datasets and code on which the conclusions of the paper rely must be either included in your submission or deposited in publicly available repositories (where available and ethically appropriate), referencing such data using a unique identifier in the references and in the "Availability of Data and Materials" section of your manuscript.<br><br>Have you have met the above requirement as detailed in our Minimum Standards Reporting Checklist?	Yes

Manuscript in pdf
Click here to download Manuscript VaDiR_bmc_new_revised_jc.pdf

RESEARCH

VaDiR: an integrated approach to Variant Detection in RNA

Lisa Neums[1,2], Seiji Suenaga[1], Peter Beyerlein[2], Sara Anders[2], Devin Koestler[4], Andrea Mariani[3] and Jeremy Chien[1*]

Abstract

Background: Advances in next-generation DNA sequencing technologies are now enabling detailed characterization of sequence variations in cancer genomes. With whole genome sequencing, variations in coding and non-coding sequences can be discovered. But the cost associated with it is currently limiting its general use in research. Whole exome sequencing is used to characterize sequence variations in coding regions, but the cost associated with capture reagents and biases in capture rate limit its full use in research. Additional limitations include uncertainty in assigning the functional significance of the mutations when these mutations are observed in the non-coding region or in genes that are not expressed in cancer tissue.

Results: We investigated the feasibility of uncovering mutations from expressed genes using RNA sequencing datasets with a method called "VaDiR: Variant Detection in RNA" that integrate three variant callers, namely: SNPiR, RVBoost and MuTect2. The combination of all three methods, which we called Tier1 variants, produced the highest precision with true positive mutations from RNA-seq that could be validated at the DNA level. We also found that the integration of Tier1 variants with those called by MuTect2 and SNPiR produced the highest recall with acceptable precision. Finally, we observed higher rate of mutation discovery in genes that are expressed at higher levels.

Conclusions: Our method, VaDiR, provides a possibility of uncovering mutations from RNA sequencing datasets that could be useful in further functional analysis. In addition, our approach allows orthogonal validation of DNA-based mutation discovery by providing complementary sequence variation analysis from paired RNA/DNA sequencing data sets.

Keywords: RNA-seq; somatic variant calling; Ovarian Cancer; Cancer genomes; Transcriptome

Background

Next-generation sequencing has enabled the discovery of novel variants in genetic sequences. However, even though the cost of sequencing has decreased in recent years, whole genome sequencing (WGS) can still be prohibitively expensive in many cases [1]. Sequencing only exonic regions of the genome helps reduce cost, and multiple tools (such as MuTect2 provided by GATK [2], MuSE [3], SomaticSniper [4] and VarScan2 [5]) have been developed for somatic variant discovery using whole exome sequencing (WES) data, and the performance of these tools was recently evaluated [6]. Still, the reagents used to capture exonic regions are costly and produce uneven coverage across the genome due to capture rate biases [7, 8], and only a fraction of the genes in an exome are actually expressed in any given cell [9]. For diseases like cancer, mutations in expressed regions are of greater interest than in non-exonic or unexpressed exonic regions because they are more likely to affect cellular function directly. The transcriptome is therefore an attractive subject of research in cancer and other human pathologies, and some of the cancer genes, such as FOXL2 in granulosa-cell tumors [10] and ARID1A in clear cell carcinomas of the ovary [11], were initially discovered through transcriptome sequencing.

The calling of variants with sequencing data from transcriptome (RNA-seq) is more challenging because of the splice junctions. Tools like RVBoost [12], SNPiR [13] or GATK Haplotypecaller are created to address this problem. Somatic variant calling from RNA is more difficult because of RNA processing like RNA-editing, allele-specific expression, variable levels of gene expression, and the heterogeneity of tumors which

*Correspondence: Chien.jeremy@gmail.com
[1]Department of Cancer Biology, University of Kansas Medical Center, 3901 Rainbow Blvd., 66160 Kansas City, KS, USA
Full list of author information is available at the end of the article

leads to low variant frequencies of some mutations [14]. Tools such as RVBoost, SNPiR, and GATK Haplotypecaller can be used to perform germline variant calling from RNA, but their performance and limitations for somatic variant calling have not been studied previously. Nonetheless, these approaches have the potential to provide an orthogonal method to validate DNA sequence variations by complementing the analysis with RNA sequence analysis.

Additional challenges include the determination of detected mutations either as germline or somatic. In tumor tissues, somatic mutations differ from the germline variations of a patient that are different from the reference genome. To detect somatic sequence variations, it is necessary to compare DNA sequences from normal tissue, such as blood, to DNA or RNA sequences from tumor tissue. If germline sequence variations are not filtered out, it would be difficult to assign detected variations as either somatic or germline. Additionally, it would be improper to assign a variant discovered in the tumor tissue as a somatic mutation when this particular position has no sufficient coverage in germline sequencing.

It should be noted that the integrated approach used by RADIA [15], that combines the somatic variant sequence analysis from tumor DNA and RNA sequencing, allows the discovery of DNA sequence variations in expressed genes and a better characterization of the effect of mutations on gene expression and phenotypic alterations. However, its use of WES of tumor tissue introduces additional cost. RADIA uses the tumor DNA and normal DNA sequencing data sets in the main analysis, and RNA sequence analysis is used as an orthogonal supplement. DNA sequence variations are considered as the ground truth, and RNA variants not supported by DNA sequencing were rejected as false-positives. Although somatic variants discovered only by RNA sequencing have the potential of being false-positives, some of these variants may represent missed calls from tumor DNA sequencing or RNA-editing sites that have not been annotated. A detailed comparison of somatic DNA and RNA variants from different tools will provide us with more precise processing and discovery of sequence variations from RNA and DNA sequencing.

In this study, following the recommendation and practices that are widely adopted in the field of bioinformatics [16, 17], we chose a validated dataset to perform a detailed comparison of somatic DNA and somatic RNA sequence variations from 21 pairs of whole exome and mRNA sequencing from ovarian cancer genomes. We formulated an approach to utilize three publicly available tools, namely MuTect2, RVboost and SNPiR for variant discovery from RNA sequencing. We evaluated the performance of each tool and established the best combination of these tools that enables discovery of variants from RNA sequence with high precision and recall. We showed that most of the variants which would be classified as false-positives or false-negatives can be explained by biological characteristics. In addition, we investigated the performance of our workflow on artificially spiked variants in coding regions of mRNA sequencing data and we compared the performance of VaDiR to RADIA. Finally, we showed the performance of our workflow on a biologically relevant study: the comparison of somatic variants in high-grade serous carcinomas collected from patients with chemotherapy-resistant or -sensitive ovarian cancer.

DATA DESCRIPTION

Twenty one samples of ovarian serous cystadenocarcinoma from The Cancer Genome Atlas (TCGA) were divided into two groups: 11 cases that were sensitive to the cancer treatment and 10 cases that were resistant. Sensitive cases had a progression-free survival of more than 18 months, and resistant cases had progression-free survival of less than 12 months. The clinical data for the patients were retrieved from cBioPortal ([18–20]), and the Illumina sequence files for tumor RNA and normal blood DNA were retrieved from cghub [21] and gdc [22] (Supplementary Table 1). Whole exome sequencing and mRNA sequencing datasets were available from each patient.

Additional data used for the artificial spiking of variants (see section "Detection of artificial spiked variants") were provided by Dr. Andrea Mariani and came from three different tumor samples from a patient with serous ovarian carcinoma.

ANALYSIS

Performance characteristics of each method and different combinations of two or more methods

To describe the performance characteristics of each method, we use recall and precision metrics instead of sensitivity and specificity because we are interested in variant calls only. Specificity is not a relevant measure because it includes all true negative calls which are in millions. We performed variant calling using RVboost, SNPiR, and MuTect2 separately. Each caller alone calls many variants which are not validated by DNA somatic variants (discordant calls), while SNPiR calls the most variants (Figure 1(A)). Mutect2 provides the least amount of variant calls not supported by DNA sequencing compared to the other two methods. However, only 10% of variant calls made by Mutect2 was supported by DNA sequencing. These results indicate that any single caller is not adequate in discovering variants with high precision. Therefore, we

next tested if any combination of three calling methods would provide a higher rate of variant calls supported by DNA sequencing. The combination of all three calling methods (hereafter referred to as Tier1) leads to 81.8% of variants which are validated by DNA somatic variants (concordant calls) with a recall rate of 9% (Figure 1(B), Supplementary Table 2). The combination of Tier1 with mutations called by Mutect2 and SNPiR (hereafter referred to as Tier2) leads to a higher recall (11.3%) while the precision is still in a moderate range (41.5%). For the following analysis, we concentrated only on Tier1.

Effect of weighted features

Additionally, we performed a weighted average of three callers with the goal of decreasing the number of false positive (FP) and false negative (FN) calls. Specifically, we investigated the effect of different weights on the evalue, which was defined as the sum of FP and FN. The weights on each of the callers were systematically varied from 0 to 1 in increments of 0.1. Evalues were calculated for each weighted combination and the optimal weights were defined as those that resulted in the smallest evalue. The consensus call of all three callers (Tier1) is denoted in blue (Figure 2). Our results demonstrate that many different combinations of weights produce similar evalues as compared to the consensus call of all three callers (Figure 2, Supplementary Figure 1), suggesting that no improvement in performance was gained by weighted average approach. Similarly, no appreciable gain in performance was noted when we considered the variant allele frequency (vaf) in the estimation of the weights (Supplementary Figure 2). Thus, taken collectively, our results showed little to no benefit in using weighted features.

Performance of a combined calling method

A total of 634 somatic mutations were called from 21 tumor samples. 516 mutations were concordant and 116 were discordant with mutation calls made from DNA (see Supplementary Table 2). To get a ground truth of variants which could have been called by RNA and were called in tumor DNA, we filtered out all DNA variant calls which have a read depth below 10 in RNA. With this filtering, we found a total of 515 variants which were called at the RNA level, while 452 of them are concordant (true-positive) and 63 discordant (false-positive) (Table 1). 1,779 of the 10,361 variants called by DNA callers have read depth greater than ten at the RNA level, and 1,327 of them were missed by RNA calling (74.5% false-negative rate).

Variants not found in RNA

To understand why variant calls from RNA sequencing missed a large majority of variant calls observed by DNA sequencing, we checked the properties of variants missed by RNA callers. From the 10,361 somatic variants called by at least two DNA variant callers, 9,845 were missed by Tier1. Out of them 8,517 (86.5%) were missed because these variants reside in genes that are not expressed (4,628) or expressed less abundantly (3,890) (Supplementary Figure 3). For the mutations in genes with high transcript abundance, 474 (4.8%) were missed because these variants were not in exonic regions. The effect of transcript abundance on variants discovered from RNA-seq could also be observed in the percentage of concordant calls: 516 (24.7%) of the expressed mutations called in DNA in exonic regions were called by Tier1 (Figure 3 (A)) but when the expression is higher (DP>10) 34.6% (452 out of 1,305 mutations) of the somatic mutations were called. This result confirms that an important factor in RNA-seq variant calling is the expression level.

Among the mutations found by DNA callers but missed by Tier1 from highly expressed genes (DP>10), 531 (5.4%) of the mutations had variant allele fraction (VAF) < 0.20 in tumor DNA, while 141 of them had a VAF $= 0$ (Figure 3 (B), Supplementary Figure 4), which can be explained through missed indels and that we accepted only reads with a high quality value in the discovery of the DP of all variants. Additionally, 724 (7.4%) of the missed mutations had VAF < 0.20 in tumor RNA, while 493 of them had a VAF $= 0$ in tumor RNA. This result confirms that one of the limitations of RNA-based variant calling methods is that they are highly dependent on the VAF. Figure 3 (B) shows that VAF of missed variant is significantly lower than VAF of called variants both at the DNA and RNA levels (p-value < 0.0001). Moreover, the difference is much greater between VAF of called variants and missed variants at the RNA levels, suggesting that many of the missed variants at the RNA level may be the result of mutations present in small fraction of tumor cells and the lower expression of mutated transcripts.

From the variants with high expression and high VAF, thirty one mutations were not called by any of the callers. Ninety six mutations were filtered out by at least one of the callers because of potential evidence of germline variants or because the realigning step with PBLAT shows that these variants could come from mismapping. Most of the missed variants with low VAF are called by MuTect2 or SNPiR alone or MuTect2 and SNPiR together(Figure 3 (C)). It is not clear if these missed variants are false-negatives, i.e, true variants missed by VADiR, or if they are false-positives made by DNA callers. Given that many of the missed variant calls (not found by VaDiR) are the result of PBLAT step in VaDiR to eliminate mis-mapped

reads and this step is not used in DNA callers, it is possible that some of the calls missed by VaDiR are true negatives that are incorrectly called by DNA callers.

Variants not found in DNA

The differences in coverage or VAF between DNA and RNA datasets could also contribute to discordant calls. Therefore, we checked those attributes at discordant sites. From all 116 discordant mutations called by Tier1, 53 (45.7%) had a read depth (DP) of uniquely mapping reads under 10 at RNA level and seventeen (15.7%) had a read depth under 10 at DNA level (Supplementary Figure 5). Another 22 (19.0%) mutations had VAF > zero at DNA level, indicating that these low-level DNA variants were missed by DNA-based callers used by TCGA. Twenty three variants with VAF=0 at DNA level but high DP in germline DNA, tumor DNA and tumor RNA were mostly either A>G or C>T (Supplementary Figure 6). Those variants were found at 12 different positions, of which one variant (chr3:58141791 A>G [FLNB:p.M2324V]) is found in 4 different samples and another (chr20:10285837 C>T) in 9 different samples. These likely represent unannotated RNA-editing sites [23–25].

Because we observed differences in the VAF at the discordant sites, we next expanded the analysis to all sites. Interestingly, we observed a weak correlation of VAF between tumor DNA and tumor RNA at positions with DP>0 for tumor DNA and RNA (Figure 4 (A)). When we limit the analysis to positions with DP>10 for tumor DNA (Figure 4 (B)) or tumor and normal DNA (Figure 4 (C)), we also observed a weak correlation. Finally, when we limit the analysis to positions with DP>10 for tumor DNA and RNA and normal DNA, we observed a strong correlation of 0.74 of variant allele fraction between RNA and DNA (Figure 4 (D)). Only four mutations had VAF around 0.50 at DNA level but 1.0 at RNA level which suggests that these are imprinted genes. These results suggest that VAF in abundant transcripts are strongly correlated with VAF at DNA level. Therefore, VAF obtained from RNA-sequencing may be used as a substitute for DNA VAF for subclone phylogenetic analysis. As shown by McPherson et al. [26] subclonal phylogenetics can use limited/targeted sequencing to identify subclones.

Detection of artificial spiked variants

To further assess the performance of RNA-based callers, we used BamSurgeon and spiked-in 200 artificial RNA sequence variants at varying variant fractions in transcriptomes from three samples of two different tumor sites from one patient. From the 200 simulated variant positions, 120 were actually spiked in because failed positions have too low read depth even if the positions for spiking were obtained from expressed genes. On average 71% of all spiked-in variants were found by each caller alone. The combination of all three callers leads to a calling of around 50% of all spiked-in mutations (Table 2, Supplementary Figure 7). By using Tier2, we were able to call 60% of all spiked-in mutations. 55.6% of the mutations missed by Tier1 but called by at least one caller are not in coding regions (Table 3). From the remaining missed variants, 15.7% have a variant allele fraction of less than 0.2 and 6.1% have high variant allele fraction but have a DP<10 in DNA.

Comparison between RADIA and VaDiR

Since RADIA performs function similar to our workflow VaDiR, we compared the performance differences between RADIA and VaDiR. RADIA uses DNA variant calling as the primary method and use RNA variant calling as a supplement. All somatic variants called by RADIA are supported by DNA-level evidence and RNA-only variants are not called by RADIA. Therefore, we limited our comparison to variants that are found at both RNA and DNA levels by RADIA and VaDiR. A total of 308 mutations were called by either RADIA or VaDiR or both in six samples. Of these, 175 mutations were called by both methods, 12 mutations were called by VaDiR only, and 121 mutations were called by RADIA only, while VAF of variants missed by VaDiR are significantly lower than VAF of variants missed by RADIA (Supplementary Figure 8). From these 121 mutations, 40 (33.1%) had a read depth below 10 in RNA. 52 (43.0%) mutations, with a read depth over 10, had VAF below 0.20. This shows again the limitation of method based only on RNA. Six of the remaining 29 variants were in non-exonic regions and would not be called by our method.

Ovarian cancer: resistant vs. sensitive

Since variant calling from RNA-seq provides both mutational status and gene expression, the number of mutations found by RNA-seq may be associated with pathologic or clinical phenotypes. In contrast, the total number of mutations found at the DNA level may not be associated with pathologic or clinical phenotype because it may be confounded by potentially non-relevant mutations in non-coding region or in genes that are not expressed. To determine if variant calling from RNA-sequencing may provide novel insights into clinical phenotype, we characterized the number of mutations in expressed genes from RNA-seq obtained from 10 chemotherapy-resistant and 11 chemotherapy-sensitive ovarian carcinomas. We considered concordant mutations only (those found by both RNA- and DNA-based callers) for the analysis. The results indicate that concordant rate is higher for Tier1 mu-

tations compared to Tier2 mutations although total number of mutations are higher in Tier2 (Figure 5 (A)). We observed higher amount of mutations in chemotherapy-sensitive ovarian carcinomas compared to chemotherapy-resistant counterparts (Figure 5 (A)). This result is consistent with previous studies indicating that sensitive tumor samples have a higher mutation rate in ovarian cancer [27]. In these samples, number of mutations was significantly higher at either DNA ($pValue = 0.017$ [Two Sample t-test, $t = -2.3474$, $df = 19$]) or RNA ($pValue = 0.03$ [Two Sample t-test, $t = -2.605$, $df = 19$]) levels in sensitive carcinomas compared to resistant carcinoma samples (Figure 5 (B)).

We next focus our analysis to variants that produce nonsynonymous mutations because they are more likely to contribute to a change in phenotype and the divergent evolution of tumor subclones. If a tumor sample is predominantly represented by a tumor subclone, VAF of nonsynonymous SNVs in that subclone will provide the largest fraction of mutations, and thus higher fractions of VAF in nonsynonymous SNVs is expected. On the other hand, if the tumor sample is represented by multiple tumor subclones, each containing subclone-specific mutations, nonsynonymous SNVs will be found at low levels in this tumor. Therefore, VAF of nonsynonymous mutations may represent clonal heterogeneity. Results, shown in Supplementary Figure 9, indicate that differences in VAF between sensitive and resistant samples are not significant. Interestingly, sensitive samples have significantly lower VAFs in non-COSMIC mutations compared to resistant samples both at the RNA ($pValue = 0.034$ [Two Sample t-test, $t = 2.1681$, $df = 62$]) and DNA level ($pValue = 0.017$ [Two Sample t-test, $t = 2.4543$, $df = 62$])(Supplementary Figure 9 (B)).

DISCUSSION

In addition to the consensus calling of variants by three methods, we tested weighted combinations of the three methods with and without considering the vaf [28]. We didn't see any improvements in the numbers of true-positive variants, false-negative variants and false-positive variants . Therefore, the approach that uses weighted average features is not implemented in our tool. However, our workflow provides the possibility of combining calls from any or all callers for further refinement or for adapting to the need of users.

With our approach, we were able to call variants with high precision. Only a small fraction of the variants which are called in RNA but not in DNA are likely false positives. The remaining discordant variants are either RNA-editing sites or are missed by DNA callers. Most of the variants called in DNA but missed by VaDiR are not in coding regions or are not expressed. We also missed many variants that have low VAF. Those are called by none of the callers, MuTect2 only, or SNPiR only. These mutations are observed at low VAFs in tumor DNA, and therefore they likely represent mutations from small subsets of tumor subclones. Finally, our approach missed approximately 15% of variants (127/853) with a high DP and a high VAF. Among the 127, 96 mutations were called by at least one method, indicating that consensus calling is too stringent or that parameters for one of the callers is not optimal. Those data are confirmed by the artificial spiked-in variants where only variants with high VAF could be called by all three callers.

The comparison to RADIA shows that VaDiR misses mainly low-frequency RNA variants while RADIA misses some high-frequency RNA variants. This result confirms the limitation of calling variants only from RNA, but it also shows that VaDiR can be used to call a great number of somatic variants without the need for tumor whole exome sequencing. It should be noted that current workflow is not completely independent of DNA sequencing since we use germline DNA sequencing to filter out germline variants. However, if the goal is to discover variants in RNA sequencing, VaDiR workflow can be modified to use MuTect2 without germline DNA and to leave out the last filtering step for DP and VAF values in germline DNA. VaDiR may be suitable for tiered studies where VaDiR can be used in the initial step to identify common variants from RNA sequencing datasets, and these candidate mutations can be confirmed by targeted DNA sequencing in a larger cohort to uncover biologically relevant somatic mutations for a specific cancer type. By focusing the initial variant discovery to expressed genes in diseased samples, follow-up validation sequencing efforts can be more targeted to limited regions of interest, thereby lowering the total cost of these genomic studies.

We were also able to find new possible RNA-editing sites, which should be investigated in future studies. Therefore, our workflow provides new capabilities that are missing in existing approaches and can be used to gain novel insight into disease phenotype. Our main concern in future studies would be to increase the number of concordant variant calls by adjustment of the filtering steps from SNPiR and RVboost and to investigate the reasons for missed somatic variants with high VAFs. Future work will also include efforts to make this tool available through a web-server for the detection of somatic variants in RNAseq.

METHODS

Software

To process the data, we used STAR, BWA-MEM, Genome Analysis Toolkit (GATK), SNPiR, RVboost, R, Picard, BEDtools, ANNOVAR, SAMtools, and BCFtools which is a part of the SAMtools package [2, 12, 13, 29–36] (Supplementary Table 3). To analyze our results, we used BAMSurgeon, R, and RADIA [15, 37]. We used reference files from Broad Institute's resource bundle [38], including the UCSC hg19 (GRCh37) reference genome, known indels from the 1000 Genomes Project, and known SNPs from dbSNP.

To validate the results that we obtained from RNA, we used somatic variants from DNA called by any two of the variant callers MuSE, MuTect2, SomaticSniper, and VarScan. We retrieved the corresponding VCF files from GDC [22].

We implemented SNPiR with the following modifications: In the file BLAT_candidates.pl at line 94, the developers incorrectly handled the information in the CIGAR-string of hardclipped reads, that resulted in a faulty shift in the base position. We corrected the code to handle CIGAR-strings correctly. This modification was necessary because our workflow differs from the SNPiR workflow in that we use hard-clipped reads. At the same location, we also added an optimization to avoid searching through more base positions than necessary. Further, we changed the filter to use PBLAT instead of BLAT, so we could utilize additional CPU threads to improve execution time. We made similar changes in the file filter_mismatch_first6bp.pl at line 84. In addition, we optimized the search algorithm in filter_intron_near_splicejuncts.pl by skipping exons and genes that do not contain a given variant position (which also introduced the requirement that SNPiR's gene annotation table be sorted by position) and moderately improve code for readability. Finally, we modified convertVCF.sh to filter out any variant whose read depth (DP) value was zero, in order to prevent division-by-zero errors that occurred with our dataset. Rather than replacing the original SNPiR files in our distribution, we have included both versions and prefixed our file names with "revised_".

For comparison with our method, we implemented RADIA with the following modification: During BLAT filtering, RADIA also incorrectly handled the hard-clipped reads. We corrected the code for the same reasons as described for the SNPiR implementation.

For creation of the figures, the R package ggplot2 [39] was used.

Aligning sequences

The procedure for the alignment to the reference genome followed GATK Best Practices [40, 41] (Figure 6). For RNA-seq, we used the STAR aligner in 2-pass mode with the parameters implemented by ENCODE project. The resulting aligned reads were processed to add read groups, sort, mark duplicates, split reads that spanned splice junctions, create an index, realign around known indels, reassign mapping qualities, and recalibrate base quality scores.

For DNA, we used the BWA-MEM aligner with the same reference genome. The resulting aligned reads were processed to add read groups, sort, mark duplicates, create an index, realign around known indels, reassign mapping qualities, and recalibrate base quality scores.

Calling variants

A refined BAM file for each sample is then used to process the variant calling. Three different methods for calling are used: RVboost, SNPiR, and MuTect2. The first two methods are for germline variants in RNA and the last method is for somatic variants in DNA. None of these methods is for somatic variant calling in RNA. RVboost and SNPiR use the same variant caller, UnifiedGenotyper from GATK, but different filtering procedures. RVboost filters variants using a statistical learning method called boosting, whereas SNPiR uses hard filtering in 7 steps (Supplementary Table 4). To adapt MuTect2's results for RNA, we implemented three of SNPiR's hard-filtering steps. RVboost and SNPiR only need the refined RNA BAM file from the tumor tissue. MuTect2 needs both the refined RNA BAM from the tumor tissue and the refined DNA BAM from normal tissue.

Filtering somatic variants by caller intersection and additional hard filters

In addition to the filtering procedures of the variant callers themselves, we further filtered our results by taking an intersection of vcf files from the three callers. We restricted our final, combined callset to the variants called by all three methods (Tier 1) or supplemented by variants called by MuTect2 and SNPiR (Tier2). We also applied our own hard filters, only accepting variants with a read depth (DP) of at least five and a VAF of less than 3% in uniquely mapping reads (Mapping quality of at least 40) in the normal DNA at the corresponding position.

Weighting of Features

For the performance of different weighted combinations of the three callers, namely SNPiR (s), Rvboost (r) and MuTect2 (m), we performed two experiments using all variants in coding regions that have a read depth DP > 10 in RNAseq. The weight w_i of caller i was calculated as follow: $w_i = \frac{v_i}{\sum_{j=1}^{3} v_j}$ where the

values v_i ranged from 0 to 1 in increments of 0.1. To find the best weighted combination, we determined an evalue, which is calculated as sum of all false-negative and all false-positive variants. Next, we calculated the area under the precision-recall curve (AUPRC), sensitivity/recall, specificity and precision.

Experiment 1 (Supplementary Table 5): For all variants called by at least one caller, we calculated the weighted score s as follows: $s = \sum_{i=1}^{3} w_i \cdot c_i$, where c_i represents call (1) or no call (0) made by caller i. We then identified the optimal threshold of s that provides the lowest evalue. This was done for each weighted combination of callers.

Experiment 2 (Supplementary Table 6): We calculated the evalue for each weighted combination to determine the optimal threshold for which the variant is called multiplied by the variant allele frequency (vaf) and adjusted to the dynamic range of the callers as follow: $s = \sum_{i=1}^{3} w_i \cdot \frac{vaf_i - mean_i}{standarddeviation_i}$. The threshold is a value of s between -3 and 3 at which the lowest evalue is achieved.

Processing artificial spiked variants

We used BAMSURGEON to spike in 200 variants in coding regions of two ovarian tumor samples, such that each sample had a different random frequency of spiked-in variants. The samples were then processed by VaDiR.

Processing samples with RADIA

Six samples from TCGA, three from resistant patients and three from sensitive patients, were processed with RADIA. This analysis required three BAM files from each sample: one from normal blood DNA, one from tumor DNA, and one from tumor RNA. We followed the instructions provided by RADIA for filtering. We used all possible filters provided by RADIA.

AVAILABILITY AND REQUIREMENTS

- Project name: somatic VaDiR
- Project home page: http://dx.doi.org/10.5524/100360
- Project RRID:SCR_015797
- Operating system(s): Linux/Unix 64-Bit
- Programming language: Perl, R, Java, Shell
- Other requirements: Java 7 and 8, R 3.3 or higher
- License: MIT
- Any restrictions to use by non-academics: no

AVAILABILITY OF SUPPORTING DATA AND MATERIALS

The data sets supporting the results of this article are available in the open science framework repository, [42], and the GDC repository, [22].

Supporting data and an archival copy of the code are also available via the GigaScience repository GigaDB [43].

For testing purposes we utilised data kindly provided by Dr. Andrea Mariani of Mayo Clinic, Rochester, Minnesota. Due to ethical constraints these data cannot be shared publicly, but if researchers would like to request access to these data please contact Dr. Andrea Mariani (mariani.andrea@mayo.edu) with a short description of why you require access and how you would use the data.

List of abbrevations

- WGS: Whole genome sequencing
- WES: Whole exome sequencing
- RNA-seq: Data from sequencing cDNA derived from RNA
- Tier1: Variants called by each caller (SNPiR, RVBoost, MuTect2)
- Tier2: Variants called by Tier1 and variants called by SNPiR and MuTect2.
- VAF: Variant allele fraction
- DP: read depth

Ethics approved and consent to participate

The datasets were obtained from the Cancer Genome Atlas, and the use of data was approved under the Project #4017 at dbGaP.

Consent for publication

Not applicable

Competing interests

The authors declare that they have no competing interests.

Funding

The study is funded by the University of Kansas Endowment Association, the University of Kansas Cancer Center Support Grant (P30-CA168524) and used the Biostatistics and Informatics Shared Resource (BISR), the Cancer Center Cancer Biology program, and the Department of Defense Ovarian Cancer Research Program under award number (W81XWH-10-1-0386). Views and opinions of, and endorsements by the author(s) do not reflect those of the US Army or the Department of Defense.

Author's contributions

- Development of workflow: Jeremy Chien and Lisa Neums
- Conception and design: Jeremy Chien and Lisa Neums
- Acquisition of data: Dr. Andrea Mariani
- Analysis and interpretation of data: Lisa Neums, Jeremy Chien, Seiji Suenaga, Devin Koestler and Sara Anders
- Writing, review, and revision of the manuscript: Jeremy Chien, Lisa Neums and Seiji Suenaga
- Administration, technical, or material support: Jeremy Chien, Peter Beyerlein Devin Koestler

Acknowledgements

The results published here are in whole or part based upon data generated by the TCGA Research Network: http://cancergenome.nih.gov/. We acknowledge Dr. Devin Koestler for helpful discussions and comments.

Author details

[1]Department of Cancer Biology, University of Kansas Medical Center, 3901 Rainbow Blvd., 66160 Kansas City, KS, USA. [2]Department of Bioinformatics and Biosystems Technology, University of Applied Sciences Wildau, Hochschulring 1, 15745 Wildau, Germany. [3]Obstetrics and Gynecology, Cancer Center, Mayo Clinic, 200 First St. SW, 55905 Rochester, MN, USA. [4]Department of Biostatistics, University of Kansas Medical Center, 3901 Rainbow Blvd., 66160 Kansas City, KS, USA.

References

1. The Cost of Sequencing a Human Genome. https://www.genome.gov/27565109/the-cost-of-sequencing-a-human-genome/
2. McKenna, A., Hanna, M., Banks, E., Sivachenko, A., Cibulskis, K., Kernytsky, A., Garimella, K., Altshuler, D., Gabriel, S., Daly, M., DePristo, M.A.: The genome analysis toolkit: a mapreduce framework for analyzing next-generation dna sequencing data. Genome Research **20**, 1297–1303 (2010)
3. Fan, Y., Xi, L., Hughes, D.S., Zhang, J., Zhang, J., Futreal, P.A., Wheeler, D.A., Wang, W.: Muse: accounting for tumor heterogeneity using a sample-specific error model improves sensitivity and specificity in mutation calling from sequencing data. Genome Biol. **17**(1), 178 (2016)
4. Larson, D.E., Harris, C.C., Chen, K., Koboldt, D.C., Abbott, T.E., Dooling, D.J., Ley, T.J., Mardis, E.R., Wilson, R.K., Ding, L.: Somaticsniper: identification of somatic point mutations in whole genome sequencing data. Bioinformatics **28**(3), 311–317 (2012)
5. Koboldt, D.C., Zhang, Q., Larson, D.E., Shen, D., McLellan, M.D., Lin, L., Miller, C.A., Mardis, E.R., Ding, L., Wilson, R.K.: Varscan 2: somatic mutation and copy number alteration discovery in cancer by exome sequencing. Genome Research **22**(3), 568–576 (2012)
6. Cai, L., Yuan, W., Zhang, Z.: In-depth comparison of somatic point mutation callers based on different tumor next-generation sequencing depth data. Scientific Reports **36540**(6) (2016)
7. Guettouche, T., Zuchner, S.: Improved coverage and accuracy with strand-conserving sequence enrichment. Genome Med **5**(5), 46 (2013)
8. Parla, J.S., Iossifov, I., Grabill, I., Spector, M.S., Kramer, M., McCombie, W.R.: A comparative analysis of exome capture. Genome Biol **12**(9), 97 (2011)
9. Garcia-Ortega, L.F., Martinez, O.: How many genes are expressed in a transcriptome? estimation and results for rna-seq. PLoS One **10**(6), 0130262 (2015)
10. Shah, S.P., Kobel, M., Senz, J., Morin, R.D., Clarke, B.A., Wiegand, K.C., Leung, G., Zayed, A., Mehl, E., Kalloger, S.E., Sun, M., Giuliany, R., Yorida, E., Jones, S., Varhol, R., Swenerton, K.D., Miller, D., Clement, P.B., Crane, C., Madore, J., Provencher, D., Leung, P., DeFazio, A., Khattra, J., Turashvili, G., Zhao, Y., Zeng, T., Glover, J.N., Vanderhyden, B., Zhao, C., Parkinson, C.A., Jimenez-Linan, M., Bowtell, D.D., Mes-Masson, A.M., Brenton, J.D., Aparicio, S.A., Boyd, N., Hirst, M., Gilks, C.B., Marra, M., Huntsman, D.G.: Mutation of foxl2 in granulosa-cell tumors of the ovary. N Engl J Med **360**(26), 2719–29 (2009)
11. Wiegand, K.C., Shah, S.P., Al-Agha, O.M., Zhao, Y., Tse, K., Zeng, T., Senz, J., McConechy, M.K., Anglesio, M.S., Kalloger, S.E., Yang, W., Heravi-Moussavi, A., Giuliany, R., Chow, C., Fee, J., Zayed, A., Prentice, L., Melnyk, N., Turashvili, G., Delaney, A.D., Madore, J., Yip, S., McPherson, A.W., Ha, G., Bell, L., Fereday, S., Tam, A., Galletta, L., Tonin, P.N., Provencher, D., Miller, D., Jones, S.J., Moore, R.A., Morin, G.B., Oloumi, A., Boyd, N., Aparicio, S.A., Shih Ie, M., Mes-Masson, A.M., Bowtell, D.D., Hirst, M., Gilks, B., Marra, M.A., Huntsman, D.G.: Arid1a mutations in endometriosis-associated ovarian carcinomas. N Engl J Med **363**(16), 1532–43 (2010)
12. Wang, C., Davila, J.I., Baheti, S., Bhagwate, A.V., Wang, X., Kocher, J.P., Slager, S.L., Feldman, A.L., Novak, A.J., Cerhan, J.R., Thompson, E.A., Asmann, Y.W.: Rvboost: Rna-seq variants prioritization using a boosting method. Bioinformatics **30**(23), 3414–3416 (2014)
13. Piskol, R., Ramaswami, G., Li, J.B.: Reliable identification of genomic variants from rna-seq data. Am J Hum Genet **93**(4), 641–651 (2013)
14. Spence, J.M., Spence, J.P., Abumoussa, A., Burack, W.R.: Ultradeep analysis of tumor heterogeneity in regions of somatic hypermutation. Genome Med **7**(1), 24 (2015)
15. Radenbaugh, A.J., Ma, S., Ewing, A., Stuart, J.M., Collisson, E.A., Zhu, J., Haussler, D.: Radia: Rna and dna integrated analysis for somatic mutation detection. PLoS One **9**(11) (2014)
16. Chou, K.C.: Some remarks on protein attribute prediction and pseudo amino acid composition. Journal of theoretical biology **1**(273), 236–47 (2011). doi:10.1016/j.jtbi.2010.12.024
17. Xu, Y., Ding, Y.-X., Ding, J., Lei, Y.-H., Wu, L.-Y., Deng, N.-Y.: isuc-pseaac: predicting lysine succinylation in proteins by incorporating peptide position-specific propensity. Scientific Reports **10184**(5) (2015). doi:10.1038/srep10184
18. cBioPortal for Cancer Genomics. http://www.cbioportal.org/
19. Gao, J., Aksoy, B.A., Dogrusoz, U., Dresdner, G., Gross, B., Sumer, S.O., Sun, Y., Jacobsen, A., Sinha, R., Larsson, E., Cerami, E., Sander, C., Schultz, N.: Integrative analysis of complex cancer genomics and clinical profiles using the cbioportal. Sci Signal **6**(269) (2013). pl1
20. Cerami, E., Gao, J., Dogrusoz, U., Gross, B.E., O, S.S., A, A.B., Jacobsen, A., Byrne, C.J., Heuer, M.L., Larsson, E., Antipin, Y., B, R., Goldberg, A.P., Sander, C., Schultz, N.: The cbio cancer genomics portal: An open platform for exploring multidimensional cancer genomics data. Cancer Discovery **2**(5), 401–404 (2012)
21. Cancer Genomics Hub. https://cghub.ucsc.edu/
22. GDC Data Portal - National Institutes of Health. https://gdc-portal.nci.nih.gov/
23. Wang, I.X., So, E., Devlin, J.L., Zhao, Y., Wu, M., Cheung, V.G.: Adar regulates rna editing, transcript stability, and gene expression. Cell Rep. **5**(3), 849–860 (2013)
24. Blanc, V., Davidson, N.O.: Apobec-1 mediated rna editing. Wiley Interdiscip Rev Syst Biol Med. **2**(5), 594–602 (2011)
25. Blanc, V., Park, E., Schaefer, S., Miller, M., Lin, Y., Kennedy, S., Billing, A.M., Ben Hamidane, H., Graumann, J., Mortazavi, A., Nadeau, J.H., Davidson, N.O.: Genome-wide identification and functional analysis of apobec-1-mediated c-to-u rna editing in mouse small intestine and liver. Genome Biol **15**(6), 79 (2014)
26. McPherson, A., Roth, A., Laks, E., Masud, T., Bashashati, A., Zhang, A.W., Ha, G., Biele, J., Yap, D., Wan, A., Prentice, L.M., Khattra, J., Smith, M.A., Nielsen, C.B., Mullaly, S.C., Kalloger, S., Karnezis, A., Shumansky, K., Siu, C., Rosner, J., Chan, H.L., Ho, J., Melnyk, N., Senz, J., Yang, W., Moore, R., Mungall, A.J., Marra, M.A., Bouchard-Côté, A., Gilks, C.B., Huntsman, D.G., McAlpine, J.N., Aparicio, S., Shah, S.P.: Divergent modes of clonal spread and intraperitoneal mixing in high-grade serous ovarian cancer. Nat Genet. **48**(7), 758–57 (2016). doi:10.1038/ng.3573
27. Birkbak, N.J., Kochupurakkal, B., Izarzugaza, J.M.G., Eklund, A.C., Y, L., Liu, J., Szallasi, Z., Matulonis, U.A., Richardson, A.L., Iglehart, J.D., Wang, Z.C.: Tumor mutation burden forecasts outcome in ovarian cancer with brca1 or brca2 mutations. PLoS ONE **8**(11), 80023 (2013)
28. Tulyakov, S., Jaeger, S., Govindaraju, V., Doermann, D.: Review of Classifier Combination Methods. In: F., S.M.H. (ed.) Studies in Computational Intelligence: Machine Learning in Document Analysis and RecognitionStudies in Computational Intelligence: Machine Learning in Document Analysis and Recognition, pp. 361–386. Springer, New York (2008)
29. Wang, K., Li, M., Hakonarson, H.: Annovar: functional annotation of genetic variants from high-throughput sequencing data. Nucleic Acids Res **38**(16), 164 (2010)
30. Dobin, A., Davis, C.A., Schlesinger, F., Drenkow, J., Zaleski, C., Jha, S., Batut, P., Chaisson, M., Gingeras, T.R.: Star: ultrafast universal rna-seq aligner. Bioinformatics **29**(1), 15–21 (2013)
31. Li, H., Durbin, R.: Fast and accurate long-read alignment with burrows-wheeler transform. Bioinformatics **26**(5), 589–95 (2010)
32. Picard. http://broadinstitute.github.io/picard
33. Li, H.: A statistical framework for snp calling, mutation discovery, association mapping and population genetical parameter estimation from sequencing data. Bioinformatics **27**(21), 2987–93 (2011)
34. Li, H., Handsaker, B., Wysoker, A., Fennell, T., Ruan, J., Homer, N., Marth, G., Abecasis, G., Durbin, R., Genome Project Data Processing, S.: The sequence alignment/map format and samtools. Bioinformatics **25**(16), 2078–9 (2009)
35. Team, R.D.C.: R: A Language and Environment for Statistical Computing. R Foundation for Statistical Computing, Vienna, Austria (2008). R Foundation for Statistical Computing. http://www.R-project.org
36. Quinlan, A.R., Hall, I.M.: Bedtools: a flexible suite of utilities for comparing genomic features. Bioinformatics **26**(6), 841–2 (2010)
37. Ewing, A.D., Houlahan, K.E., Hu, Y., Ellrott, K., Caloian, C., Yamaguchi, T.N., Bare, J.C., P'ng, C., Waggott, D., Sabelnykova, V.Y., participants, I.-T.D.S.M.C.C., Kellen, M.R., Norman, T.C., Haussler, D., Friend, S.H., Stolovitzky, G., Margolin, A.A., Stuart,

J.M., Boutros, P.C.: Combining tumor genome simulation with crowdsourcing to benchmark somatic single-nucleotide-variant detection. Nat Methods **12**(7), 623–30 (2015)
38. Broad Institute's Resource Bundle. ftp://ftp.broadinstitute.org/bundle/2.8/hg19/
39. Wickham, H.: Ggplot2: Elegant Graphics for Data Analysis. Springer, ??? (2009). http://ggplot2.org
40. DePristo, M., Banks, E., Poplin, R., Garimella, K., Maguire, J., Hartl, C., Philippakis, A., del Angel, G., Rivas, M.A., Hanna, M., McKenna, A., Fennell, T., Kernytsky, A., Sivachenko, A., Cibulskis, K., Gabriel, S., Altshuler, D., Daly, M.: A framework for variation discovery and genotyping using next-generation dna sequencing data. NATURE GENETICS **43**, 491–498 (2011)
41. Van der Auwera, G.A., Carneiro, M., Hartl, C., Poplin, R., del Angel, G., Levy-Moonshine, A., Jordan, T., Shakir, K., Roazen, D., Thibault, J., Banks, E., Garimella, K., Altshuler, D., Gabriel, S., DePristo, M.: From fastq data to high-confidence variant calls: The genome analysis toolkit best practices pipeline. CURRENT PROTOCOLS IN BIOINFORMATICS **43**, 11101–111033 (2013)
42. Open Science Framework Repository for VaDiR Data. http://www.osf.io/ap5b7
43. Anders, S., Beyerlein, P., Chien, J., Koestler, D., Mariani, A., Neums, L., Suenaga, S.: Supporting software for "VaDiR: an integrated approach to Variant Detection in RNA" GigaScience Database. http://dx.doi.org/10.5524/100360. VaDiR, RRID:SCR_015797 (2017)

Illustrations and figures

Figure 1 Intersection of the three variant calling methods. (A) Intersection of the three methods with all somatic variants. The red triangles represent the amount of concordant variants. **(B)** Intersection of three methods with only concordant somatic variants. All three callers (Tier1) together has the highest number of condordant variants.

Figure 2 Effect of weighted features on the performance Shown is in panel **A** to **D** the performance of each combinationo of weights of the three callers Haplotypecaller, SNPiR and RVboost while evalue means the sum of FP and FN. The blue point marks the equal combination of all three callers, namly Tier1. In Panel **E** is shown the weights of the callers in each combination.

Figure 3 Variants called in tumor DNA. (A) Percentage of concordant calls of all somatic variants from expressed genes for each sample from two datasets (sensitive and resistant tumor samples). A higher percentage of concordant calls was achieved in transcripts with high expression (DP>10) compared to that of all expressed transcripts (DP>0). **(B)** Violin plot of variant fraction for all somatic variant positions with RNA DP>10. Most of the variant positions missed by VaDiR have a low variant fraction (VAF<0.1) in RNA. **(C)** Ranked SNVs called by TCGA and/or different combinations of RNA-seq calling methods. Only those positions with DP>10 in tumor DNA, RNA and normal DNA are included in the analysis. The names in the chart are the first letters of the caller SNPiR (s), RVBoost (r) and MuTect (m) or their combinations.

Tables and captions

Figure 4 Correlation of variant fractions between RNA and DNA. The four charts show the effect of read depth filter on the correlation of variant fractions.

Figure 5 Comparison of sensitive and resistant samples. (A) Numbers of concordant calls in Tier1 and Tier2 by VaDiR. The precision for each sample for Tier1 and Tier2 is shown in percentage above each bar. **(B)** Number of mutations found at the DNA and RNA level in sensitive tumors are significantly higher than in resistant tumor Samples.

Figure 6 VaDiR workflow for processing somatic variant calls from RNA-seq. Sequence alignment is done by STAR and BWA MEM for RNA and DNA respectively. The refined mapping follows GATK Best Practices. The variant calling is done by Unified Genotyper (GATK) and MuTect2 (GATK). The following filtering steps are done by RVBoost and SNPiR. Additional filters such as MAQ > 40, germline read depth (DP) > 5 and germline variant fraction (VAF) < 0.03 are applied to remove germline variants.

Table 1 Performance characteristics of VaDiR with the combination Tier1.

	DNA positive	DNA negative
RNA positive	452	63
RNA negative	1327	

Table 2 Called spiked-in variants.

Sample	Tier1	Tier2
OV10	68 (54.40%)	78 (62.40%)
OV11	61 (52.59%)	68 (58.62%)
OV12	58 (48.74%)	69 (57.98%)

Percentages represent recall rates in each sample. Tier 1 is the consensus of three callers. Tier 2 is the Tier 1 plus consensus of MuTect2 and SNPiR. Total number of recoverable spiked-in variants is 125 (OV10), 116 (OV11), and 119 (OV12).

Table 3 Characteristics of missed spiked-in variants.

Tier1	OV10	OV11	OV12
all spiked in variants	125	116	119
missed by VaDiR	57	55	61
not called by at least one caller	20	20	20
missed in coding region	16	17	18
missed in coding region by RNA VAF>20%	11	9	13
missed in coding region by RNA VAF>20% and normal DNA DP>10	8	7	11
**Tier2**	**OV10**	**OV11**	**OV12**
all spiked in variants	125	116	119
missed by VaDiR	47	48	50
not called by at least one caller	20	20	20
missed in coding region	9	11	12
missed in coding region by RNA VAF>20%	6	5	9
missed in coding region by RNA VAF>20% and normal DNA DP>10	4	4	8

.tex file
Click here to download Manuscript VaDiR_bmc_new_revised_jc.tex

RESEARCH

VaDiR: an integrated approach to Variant Detection in RNA

Lisa Neums[1,2], Seiji Suenaga[1], Peter Beyerlein[2], Sara Anders[2], Devin Koestler[4], Andrea Mariani[3] and Jeremy Chien[1*]

Abstract

Background: Advances in next-generation DNA sequencing technologies are now enabling detailed characterization of sequence variations in cancer genomes. With whole genome sequencing, variations in coding and non-coding sequences can be discovered. But the cost associated with it is currently limiting its general use in research. Whole exome sequencing is used to characterize sequence variations in coding regions, but the cost associated with capture reagents and biases in capture rate limit its full use in research. Additional limitations include uncertainty in assigning the functional significance of the mutations when these mutations are observed in the non-coding region or in genes that are not expressed in cancer tissue.

Results: We investigated the feasibility of uncovering mutations from expressed genes using RNA sequencing datasets with a method called "VaDiR: Variant Detection in RNA" that integrate three variant callers, namely: SNPiR, RVBoost and MuTect2. The combination of all three methods, which we called Tier1 variants, produced the highest precision with true positive mutations from RNA-seq that could be validated at the DNA level. We also found that the integration of Tier1 variants with those called by MuTect2 and SNPiR produced the highest recall with acceptable precision. Finally, we observed higher rate of mutation discovery in genes that are expressed at higher levels.

Conclusions: Our method, VaDiR, provides a possibility of uncovering mutations from RNA sequencing datasets that could be useful in further functional analysis. In addition, our approach allows orthogonal validation of DNA-based mutation discovery by providing complementary sequence variation analysis from paired RNA/DNA sequencing data sets.

Keywords: RNA-seq; somatic variant calling; Ovarian Cancer; Cancer genomes; Transcriptome

Background

Next-generation sequencing has enabled the discovery of novel variants in genetic sequences. However, even though the cost of sequencing has decreased in recent years, whole genome sequencing (WGS) can still be prohibitively expensive in many cases [1]. Sequencing only exonic regions of the genome helps reduce cost, and multiple tools (such as MuTect2 provided by GATK [2], MuSE [3], SomaticSniper [4] and VarScan2 [5]) have been developed for somatic variant discovery using whole exome sequencing (WES) data, and the performance of these tools was recently evaluated [6]. Still, the reagents used to capture exonic regions are costly and produce uneven coverage across the genome due to capture rate biases [7, 8], and only a fraction of the genes in an exome are actually expressed in any given cell [9]. For diseases like cancer, mutations in expressed regions are of greater interest than in non-exonic or unexpressed exonic regions because they are more likely to affect cellular function directly. The transcriptome is therefore an attractive subject of research in cancer and other human pathologies, and some of the cancer genes, such as FOXL2 in granulosa-cell tumors [10] and ARID1A in clear cell carcinomas of the ovary [11], were initially discovered through transcriptome sequencing.

The calling of variants with sequencing data from transcriptome (RNA-seq) is more challenging because of the splice junctions. Tools like RVBoost [12], SNPiR [13] or GATK Haplotypecaller are created to address this problem. Somatic variant calling from RNA is more difficult because of RNA processing like RNA-editing, allele-specific expression, variable levels of gene expression, and the heterogeneity of tumors which

*Correspondence: Chien.jeremy@gmail.com
[1]Department of Cancer Biology, University of Kansas Medical Center, 3901 Rainbow Blvd., 66160 Kansas City, KS, USA
Full list of author information is available at the end of the article

leads to low variant frequencies of some mutations [14]. Tools such as RVBoost, SNPiR, and GATK Haplotypecaller can be used to perform germline variant calling from RNA, but their performance and limitations for somatic variant calling have not been studied previously. Nonetheless, these approaches have the potential to provide an orthogonal method to validate DNA sequence variations by complementing the analysis with RNA sequence analysis.

Additional challenges include the determination of detected mutations either as germline or somatic. In tumor tissues, somatic mutations differ from the germline variations of a patient that are different from the reference genome. To detect somatic sequence variations, it is necessary to compare DNA sequences from normal tissue, such as blood, to DNA or RNA sequences from tumor tissue. If germline sequence variations are not filtered out, it would be difficult to assign detected variations as either somatic or germline. Additionally, it would be improper to assign a variant discovered in the tumor tissue as a somatic mutation when this particular position has no sufficient coverage in germline sequencing.

It should be noted that the integrated approach used by RADIA [15], that combines the somatic variant sequence analysis from tumor DNA and RNA sequencing, allows the discovery of DNA sequence variations in expressed genes and a better characterization of the effect of mutations on gene expression and phenotypic alterations. However, its use of WES of tumor tissue introduces additional cost. RADIA uses the tumor DNA and normal DNA sequencing data sets in the main analysis, and RNA sequence analysis is used as an orthogonal supplement. DNA sequence variations are considered as the ground truth, and RNA variants not supported by DNA sequencing were rejected as false-positives. Although somatic variants discovered only by RNA sequencing have the potential of being false-positives, some of these variants may represent missed calls from tumor DNA sequencing or RNA-editing sites that have not been annotated. A detailed comparison of somatic DNA and RNA variants from different tools will provide us with more precise processing and discovery of sequence variations from RNA and DNA sequencing.

In this study, following the recommendation and practices that are widely adopted in the field of bioinformatics [16, 17], we chose a validated dataset to perform a detailed comparison of somatic DNA and somatic RNA sequence variations from 21 pairs of whole exome and mRNA sequencing from ovarian cancer genomes. We formulated an approach to utilize three publicly available tools, namely MuTect2, RVboost and SNPiR for variant discovery from RNA sequencing. We evaluated the performance of each tool and established the best combination of these tools that enables discovery of variants from RNA sequence with high precision and recall. We showed that most of the variants which would be classified as false-positives or false-negatives can be explained by biological characteristics. In addition, we investigated the performance of our workflow on artificially spiked variants in coding regions of mRNA sequencing data and we compared the performance of VaDiR to RADIA. Finally, we showed the performance of our workflow on a biologically relevant study: the comparison of somatic variants in high-grade serous carcinomas collected from patients with chemotherapy-resistant or -sensitive ovarian cancer.

DATA DESCRIPTION

Twenty one samples of ovarian serous cystadenocarcinoma from The Cancer Genome Atlas (TCGA) were divided into two groups: 11 cases that were sensitive to the cancer treatment and 10 cases that were resistant. Sensitive cases had a progression-free survival of more than 18 months, and resistant cases had progression-free survival of less than 12 months. The clinical data for the patients were retrieved from cBioPortal ([18–20]), and the Illumina sequence files for tumor RNA and normal blood DNA were retrieved from cghub [21] and gdc [22] (Supplementary Table 1). Whole exome sequencing and mRNA sequencing datasets were available from each patient.

Additional data used for the artificial spiking of variants (see section "Detection of artificial spiked variants") were provided by Dr. Andrea Mariani and came from three different tumor samples from a patient with serous ovarian carcinoma.

ANALYSIS

Performance characteristics of each method and different combinations of two or more methods

To describe the performance characteristics of each method, we use recall and precision metrics instead of sensitivity and specificity because we are interested in variant calls only. Specificity is not a relevant measure because it includes all true negative calls which are in millions. We performed variant calling using RV-boost, SNPiR, and MuTect2 separately. Each caller alone calls many variants which are not validated by DNA somatic variants (discordant calls), while SNPiR calls the most variants (Figure 1(A)). Mutect2 provides the least amount of variant calls not supported by DNA sequencing compared to the other two methods. However, only 10% of variant calls made by Mutect2 was supported by DNA sequencing. These results indicate that any single caller is not adequate in discovering variants with high precision. Therefore, we

next tested if any combination of three calling methods would provide a higher rate of variant calls supported by DNA sequencing. The combination of all three calling methods (hereafter referred to as Tier1) leads to 81.8% of variants which are validated by DNA somatic variants (concordant calls) with a recall rate of 9% (Figure 1(B), Supplementary Table 2). The combination of Tier1 with mutations called by Mutect2 and SNPiR (hereafter referred to as Tier2) leads to a higher recall (11.3%) while the precision is still in a moderate range (41.5%). For the following analysis, we concentrated only on Tier1.

Effect of weighted features

Additionally, we performed a weighted average of three callers with the goal of decreasing the number of false positive (FP) and false negative (FN) calls. Specifically, we investigated the effect of different weights on the evalue, which was defined as the sum of FP and FN. The weights on each of the callers were systematically varied from 0 to 1 in increments of 0.1. Evalues were calculated for each weighted combination and the optimal weights were defined as those that resulted in the smallest evalue. The consensus call of all three callers (Tier1) is denoted in blue (Figure 2). Our results demonstrate that many different combinations of weights produce similar evalues as compared to the consensus call of all three callers (Figure 2, Supplementary Figure 1), suggesting that no improvement in performance was gained by weighted average approach. Similarly, no appreciable gain in performance was noted when we considered the variant allele frequency (vaf) in the estimation of the weights (Supplementary Figure 2). Thus, taken collectively, our results showed little to no benefit in using weighted features.

Performance of a combined calling method

A total of 634 somatic mutations were called from 21 tumor samples. 516 mutations were concordant and 116 were discordant with mutation calls made from DNA (see Supplementary Table 2). To get a ground truth of variants which could have been called by RNA and were called in tumor DNA, we filtered out all DNA variant calls which have a read depth below 10 in RNA. With this filtering, we found a total of 515 variants which were called at the RNA level, while 452 of them are concordant (true-positive) and 63 discordant (false-positive) (Table 1). 1,779 of the 10,361 variants called by DNA callers have read depth greater than ten at the RNA level, and 1,327 of them were missed by RNA calling (74.5% false-negative rate).

Variants not found in RNA

To understand why variant calls from RNA sequencing missed a large majority of variant calls observed by DNA sequencing, we checked the properties of variants missed by RNA callers. From the 10,361 somatic variants called by at least two DNA variant callers, 9,845 were missed by Tier1. Out of them 8,517 (86.5%) were missed because these variants reside in genes that are not expressed (4,628) or expressed less abundantly (3,890) (Supplementary Figure 3). For the mutations in genes with high transcript abundance, 474 (4.8%) were missed because these variants were not in exonic regions. The effect of transcript abundance on variants discovered from RNA-seq could also be observed in the percentage of concordant calls: 516 (24.7%) of the expressed mutations called in DNA in exonic regions were called by Tier1 (Figure 3 (A)) but when the expression is higher (DP>10) 34.6% (452 out of 1,305 mutations) of the somatic mutations were called. This result confirms that an important factor in RNA-seq variant calling is the expression level.

Among the mutations found by DNA callers but missed by Tier1 from highly expressed genes (DP>10), 531 (5.4%) of the mutations had variant allele fraction (VAF) < 0.20 in tumor DNA, while 141 of them had a VAF $= 0$ (Figure 3 (B), Supplementary Figure 4), which can be explained through missed indels and that we accepted only reads with a high quality value in the discovery of the DP of all variants. Additionally, 724 (7.4%) of the missed mutations had VAF < 0.20 in tumor RNA, while 493 of them had a VAF $= 0$ in tumor RNA. This result confirms that one of the limitations of RNA-based variant calling methods is that they are highly dependent on the VAF. Figure 3 (B) shows that VAF of missed variant is significantly lower than VAF of called variants both at the DNA and RNA levels (p-value < 0.0001). Moreover, the difference is much greater between VAF of called variants and missed variants at the RNA levels, suggesting that many of the missed variants at the RNA level may be the result of mutations present in small fraction of tumor cells and the lower expression of mutated transcripts.

From the variants with high expression and high VAF, thirty one mutations were not called by any of the callers. Ninety six mutations were filtered out by at least one of the callers because of potential evidence of germline variants or because the realigning step with PBLAT shows that these variants could come from mismapping. Most of the missed variants with low VAF are called by MuTect2 or SNPiR alone or MuTect2 and SNPiR together(Figure 3 (C)). It is not clear if these missed variants are false-negatives, i.e, true variants missed by VADiR, or if they are false-positives made by DNA callers. Given that many of the missed variant calls (not found by VaDiR) are the result of PBLAT step in VaDiR to eliminate mis-mapped

reads and this step is not used in DNA callers, it is possible that some of the calls missed by VaDiR are true negatives that are incorrectly called by DNA callers.

Variants not found in DNA
The differences in coverage or VAF between DNA and RNA datasets could also contribute to discordant calls. Therefore, we checked those attributes at discordant sites. From all 116 discordant mutations called by Tier1, 53 (45.7%) had a read depth (DP) of uniquely mapping reads under 10 at RNA level and seventeen (15.7%) had a read depth under 10 at DNA level (Supplementary Figure 5). Another 22 (19.0%) mutations had VAF > zero at DNA level, indicating that these low-level DNA variants were missed by DNA-based callers used by TCGA. Twenty three variants with VAF=0 at DNA level but high DP in germline DNA, tumor DNA and tumor RNA were mostly either A>G or C>T (Supplementary Figure 6). Those variants were found at 12 different positions, of which one variant (chr3:58141791 A>G [FLNB:p.M2324V]) is found in 4 different samples and another (chr20:10285837 C>T) in 9 different samples. These likely represent unannotated RNA-editing sites [23–25].

Because we observed differences in the VAF at the discordant sites, we next expanded the analysis to all sites. Interestingly, we observed a weak correlation of VAF between tumor DNA and tumor RNA at positions with DP>0 for tumor DNA and RNA (Figure 4 (A)). When we limit the analysis to positions with DP>10 for tumor DNA (Figure 4 (B)) or tumor and normal DNA (Figure 4 (C)), we also observed a weak correlation. Finally, when we limit the analysis to positions with DP>10 for tumor DNA and RNA and normal DNA, we observed a strong correlation of 0.74 of variant allele fraction between RNA and DNA (Figure 4 (D)). Only four mutations had VAF around 0.50 at DNA level but 1.0 at RNA level which suggests that these are imprinted genes. These results suggest that VAF in abundant transcripts are strongly correlated with VAF at DNA level. Therefore, VAF obtained from RNA-sequencing may be used as a substitute for DNA VAF for subclone phylogenetic analysis. As shown by McPherson et al. [26] subclonal phylogenetics can use limited/targeted sequencing to identify subclones.

Detection of artificial spiked variants
To further assess the performance of RNA-based callers, we used BamSurgeon and spiked-in 200 artificial RNA sequence variants at varying variant fractions in transcriptomes from three samples of two different tumor sites from one patient. From the 200 simulated variant positions, 120 were actually spiked in because failed positions have too low read depth even if the positions for spiking were obtained from expressed genes. On average 71% of all spiked-in variants were found by each caller alone. The combination of all three callers leads to a calling of around 50% of all spiked-in mutations (Table 2, Supplementary Figure 7). By using Tier2, we were able to call 60% of all spiked-in mutations. 55.6% of the mutations missed by Tier1 but called by at least one caller are not in coding regions (Table 3). From the remaining missed variants, 15.7% have a variant allele fraction of less than 0.2 and 6.1% have high variant allele fraction but have a DP<10 in DNA.

Comparison between RADIA and VaDiR
Since RADIA performs function similar to our workflow VaDiR, we compared the performance differences between RADIA and VaDiR. RADIA uses DNA variant calling as the primary method and use RNA variant calling as a supplement. All somatic variants called by RADIA are supported by DNA-level evidence and RNA-only variants are not called by RADIA. Therefore, we limited our comparison to variants that are found at both RNA and DNA levels by RADIA and VaDiR. A total of 308 mutations were called by either RADIA or VaDiR or both in six samples. Of these, 175 mutations were called by both methods, 12 mutations were called by VaDiR only, and 121 mutations were called by RADIA only, while VAF of variants missed by VaDiR are significantly lower than VAF of variants missed by RADIA (Supplementary Figure 8). From these 121 mutations, 40 (33.1%) had a read depth below 10 in RNA. 52 (43.0%) mutations, with a read depth over 10, had VAF below 0.20. This shows again the limitation of method based only on RNA. Six of the remaining 29 variants were in non-exonic regions and would not be called by our method.

Ovarian cancer: resistant vs. sensitive
Since variant calling from RNA-seq provides both mutational status and gene expression, the number of mutations found by RNA-seq may be associated with pathologic or clinical phenotypes. In contrast, the total number of mutations found at the DNA level may not be associated with pathologic or clinical phenotype because it may be confounded by potentially non-relevant mutations in non-coding region or in genes that are not expressed. To determine if variant calling from RNA-sequencing may provide novel insights into clinical phenotype, we characterized the number of mutations in expressed genes from RNA-seq obtained from 10 chemotherapy-resistant and 11 chemotherapy-sensitive ovarian carcinomas. We considered concordant mutations only (those found by both RNA- and DNA-based callers) for the analysis. The results indicate that concordant rate is higher for Tier1 mu-

tations compared to Tier2 mutations although total number of mutations are higher in Tier2 (Figure 5 (A)). We observed higher amount of mutations in chemotherapy-sensitive ovarian carcinomas compared to chemotherapy-resistant counterparts (Figure 5 (A)). This result is consistent with previous studies indicating that sensitive tumor samples have a higher mutation rate in ovarian cancer [27]. In these samples, number of mutations was significantly higher at either DNA ($pValue = 0.017$ [Two Sample t-test, $t = -2.3474$, $df = 19$]) or RNA ($pValue = 0.03$ [Two Sample t-test, $t = -2.605$, $df = 19$]) levels in sensitive carcinomas compared to resistant carcinoma samples (Figure 5 (B)).

We next focus our analysis to variants that produce nonsynonymous mutations because they are more likely to contribute to a change in phenotype and the divergent evolution of tumor subclones. If a tumor sample is predominantly represented by a tumor subclone, VAF of nonsynonymous SNVs in that subclone will provide the largest fraction of mutations, and thus higher fractions of VAF in nonsynonymous SNVs is expected. On the other hand, if the tumor sample is represented by multiple tumor subclones, each containing subclone-specific mutations, nonsynonymous SNVs will be found at low levels in this tumor. Therefore, VAF of nonsynonymous mutations may represent clonal heterogeneity. Results, shown in Supplementary Figure 9, indicate that differences in VAF between sensitive and resistant samples are not significant. Interestingly, sensitive samples have significantly lower VAFs in non-COSMIC mutations compared to resistant samples both at the RNA ($pValue = 0.034$ [Two Sample t-test, $t = 2.1681$, $df = 62$]) and DNA level ($pValue = 0.017$ [Two Sample t-test, $t = 2.4543$, $df = 62$])(Supplementary Figure 9 (B)).

DISCUSSION

In addition to the consensus calling of variants by three methods, we tested weighted combinations of the three methods with and without considering the vaf [28]. We didn't see any improvements in the numbers of true-positive variants, false-negative variants and false-positive variants . Therefore, the approach that uses weighted average features is not implemented in our tool. However, our workflow provides the possibility of combining calls from any or all callers for further refinement or for adapting to the need of users.

With our approach, we were able to call variants with high precision. Only a small fraction of the variants which are called in RNA but not in DNA are likely false positives. The remaining discordant variants are either RNA-editing sites or are missed by DNA callers. Most of the variants called in DNA but missed by VaDiR are not in coding regions or are not expressed. We also missed many variants that have low VAF. Those are called by none of the callers, MuTect2 only, or SNPiR only. These mutations are observed at low VAFs in tumor DNA, and therefore they likely represent mutations from small subsets of tumor subclones. Finally, our approach missed approximately 15% of variants (127/853) with a high DP and a high VAF. Among the 127, 96 mutations were called by at least one method, indicating that consensus calling is too stringent or that parameters for one of the callers is not optimal. Those data are confirmed by the artificial spiked-in variants where only variants with high VAF could be called by all three callers.

The comparison to RADIA shows that VaDiR misses mainly low-frequency RNA variants while RADIA misses some high-frequency RNA variants. This result confirms the limitation of calling variants only from RNA, but it also shows that VaDiR can be used to call a great number of somatic variants without the need for tumor whole exome sequencing. It should be noted that current workflow is not completely independent of DNA sequencing since we use germline DNA sequencing to filter out germline variants. However, if the goal is to discover variants in RNA sequencing, VaDiR workflow can be modified to use MuTect2 without germline DNA and to leave out the last filtering step for DP and VAF values in germline DNA. VaDiR may be suitable for tiered studies where VaDiR can be used in the initial step to identify common variants from RNA sequencing datasets, and these candidate mutations can be confirmed by targeted DNA sequencing in a larger cohort to uncover biologically relevant somatic mutations for a specific cancer type. By focusing the initial variant discovery to expressed genes in diseased samples, follow-up validation sequencing efforts can be more targeted to limited regions of interest, thereby lowering the total cost of these genomic studies.

We were also able to find new possible RNA-editing sites, which should be investigated in future studies. Therefore, our workflow provides new capabilities that are missing in existing approaches and can be used to gain novel insight into disease phenotype. Our main concern in future studies would be to increase the number of concordant variant calls by adjustment of the filtering steps from SNPiR and RVboost and to investigate the reasons for missed somatic variants with high VAFs. Future work will also include efforts to make this tool available through a web-server for the detection of somatic variants in RNAseq.

METHODS

Software

To process the data, we used STAR, BWA-MEM, Genome Analysis Toolkit (GATK), SNPiR, RVboost, R, Picard, BEDtools, ANNOVAR, SAMtools, and BCFtools which is a part of the SAMtools package [2, 12, 13, 29–36] (Supplementary Table 3). To analyze our results, we used BAMSurgeon, R, and RADIA [15, 37]. We used reference files from Broad Institute's resource bundle [38], including the UCSC hg19 (GRCh37) reference genome, known indels from the 1000 Genomes Project, and known SNPs from dbSNP.

To validate the results that we obtained from RNA, we used somatic variants from DNA called by any two of the variant callers MuSE, MuTect2, SomaticSniper, and VarScan. We retrieved the corresponding VCF files from GDC [22].

We implemented SNPiR with the following modifications: In the file BLAT_candidates.pl at line 94, the developers incorrectly handled the information in the CIGAR-string of hardclipped reads, that resulted in a faulty shift in the base position. We corrected the code to handle CIGAR-strings correctly. This modification was necessary because our workflow differs from the SNPiR workflow in that we use hard-clipped reads. At the same location, we also added an optimization to avoid searching through more base positions than necessary. Further, we changed the filter to use PBLAT instead of BLAT, so we could utilize additional CPU threads to improve execution time. We made similar changes in the file filter_mismatch_first6bp.pl at line 84. In addition, we optimized the search algorithm in filter_intron_near_splicejuncts.pl by skipping exons and genes that do not contain a given variant position (which also introduced the requirement that SNPiR's gene annotation table be sorted by position) and moderately improve code for readability. Finally, we modified convertVCF.sh to filter out any variant whose read depth (DP) value was zero, in order to prevent division-by-zero errors that occurred with our dataset. Rather than replacing the original SNPiR files in our distribution, we have included both versions and prefixed our file names with "revised_".

For comparison with our method, we implemented RADIA with the following modification: During BLAT filtering, RADIA also incorrectly handled the hard-clipped reads. We corrected the code for the same reasons as described for the SNPiR implementation.

For creation of the figures, the R package ggplot2 [39] was used.

Aligning sequences

The procedure for the alignment to the reference genome followed GATK Best Practices [40, 41] (Figure 6). For RNA-seq, we used the STAR aligner in 2-pass mode with the parameters implemented by ENCODE project. The resulting aligned reads were processed to add read groups, sort, mark duplicates, split reads that spanned splice junctions, create an index, realign around known indels, reassign mapping qualities, and recalibrate base quality scores.

For DNA, we used the BWA-MEM aligner with the same reference genome. The resulting aligned reads were processed to add read groups, sort, mark duplicates, create an index, realign around known indels, reassign mapping qualities, and recalibrate base quality scores.

Calling variants

A refined BAM file for each sample is then used to process the variant calling. Three different methods for calling are used: RVboost, SNPiR, and MuTect2. The first two methods are for germline variants in RNA and the last method is for somatic variants in DNA. None of these methods is for somatic variant calling in RNA. RVboost and SNPiR use the same variant caller, UnifiedGenotyper from GATK, but different filtering procedures. RVboost filters variants using a statistical learning method called boosting, whereas SNPiR uses hard filtering in 7 steps (Supplementary Table 4). To adapt MuTect2's results for RNA, we implemented three of SNPiR's hard-filtering steps. RVboost and SNPiR only need the refined RNA BAM file from the tumor tissue. MuTect2 needs both the refined RNA BAM from the tumor tissue and the refined DNA BAM from normal tissue.

Filtering somatic variants by caller intersection and additional hard filters

In addition to the filtering procedures of the variant callers themselves, we further filtered our results by taking an intersection of vcf files from the three callers. We restricted our final, combined callset to the variants called by all three methods (Tier 1) or supplemented by variants called by MuTect2 and SNPiR (Tier2). We also applied our own hard filters, only accepting variants with a read depth (DP) of at least five and a VAF of less than 3% in uniquely mapping reads (Mapping quality of at least 40) in the normal DNA at the corresponding position.

Weighting of Features

For the performance of different weighted combinations of the three callers, namely SNPiR (s), Rvboost (r) and MuTect2 (m), we performed two experiments using all variants in coding regions that have a read depth DP > 10 in RNAseq. The weight w_i of caller i was calculated as follow: $w_i = \frac{v_i}{\sum_{j=1}^{3} v_j}$ where the

values v_i ranged from 0 to 1 in increments of 0.1. To find the best weighted combination, we determined an evalue, which is calculated as sum of all false-negative and all false-positive variants. Next, we calculated the area under the precision-recall curve (AUPRC), sensitivity/recall, specificity and precision.

Experiment 1 (Supplementary Table 5): For all variants called by at least one caller, we calculated the weighted score s as follows: $s = \sum_{i=1}^{3} w_i \cdot c_i$, where c_i represents call (1) or no call (0) made by caller i. We then identified the optimal threshold of s that provides the lowest evalue. This was done for each weighted combination of callers.

Experiment 2 (Supplementary Table 6): We calculated the evalue for each weighted combination to determine the optimal threshold for which the variant is called multiplied by the variant allele frequency (vaf) and adjusted to the dynamic range of the callers as follow: $s = \sum_{i=1}^{3} w_i \cdot \frac{vaf_i - mean_i}{standarddeviation_i}$. The threshold is a value of s between -3 and 3 at which the lowest evalue is achieved.

Processing artificial spiked variants

We used BAMSURGEON to spike in 200 variants in coding regions of two ovarian tumor samples, such that each sample had a different random frequency of spiked-in variants. The samples were then processed by VaDiR.

Processing samples with RADIA

Six samples from TCGA, three from resistant patients and three from sensitive patients, were processed with RADIA. This analysis required three BAM files from each sample: one from normal blood DNA, one from tumor DNA, and one from tumor RNA. We followed the instructions provided by RADIA for filtering. We used all possible filters provided by RADIA.

AVAILABILITY AND REQUIREMENTS

- Project name: somatic VaDiR
- Project home page: http://dx.doi.org/10.5524/100360
- Project RRID:SCR_015797
- Operating system(s): Linux/Unix 64-Bit
- Programming language: Perl, R, Java, Shell
- Other requirements: Java 7 and 8, R 3.3 or higher
- License: MIT
- Any restrictions to use by non-academics: no

AVAILABILITY OF SUPPORTING DATA AND MATERIALS

The data sets supporting the results of this article are available in the open science framework repository, [42], and the GDC repository, [22].

Supporting data and an archival copy of the code are also available via the GigaScience repository GigaDB [43].

For testing purposes we utilised data kindly provided by Dr. Andrea Mariani of Mayo Clinic, Rochester, Minnesota. Due to ethical constraints these data cannot be shared publicly, but if researchers would like to request access to these data please contact Dr. Andrea Mariani (mariani.andrea@mayo.edu) with a short description of why you require access and how you would use the data.

List of abbrevations

- WGS: Whole genome sequencing
- WES: Whole exome sequencing
- RNA-seq: Data from sequencing cDNA derived from RNA
- Tier1: Variants called by each caller (SNPiR, RVBoost, MuTect2)
- Tier2: Variants called by Tier1 and variants called by SNPiR and MuTect2.
- VAF: Variant allele fraction
- DP: read depth

Ethics approved and consent to participate

The datasets were obtained from the Cancer Genome Atlas, and the use of data was approved under the Project #4017 at dbGaP.

Consent for publication

Not applicable

Competing interests

The authors declare that they have no competing interests.

Funding

The study is funded by the University of Kansas Endowment Association, the University of Kansas Cancer Center Support Grant (P30-CA168524) and used the Biostatistics and Informatics Shared Resource (BISR), the Cancer Center Cancer Biology program, and the Department of Defense Ovarian Cancer Research Program under award number (W81XWH-10-1-0386). Views and opinions of, and endorsements by the author(s) do not reflect those of the US Army or the Department of Defense.

Author's contributions

- Development of workflow: Jeremy Chien and Lisa Neums
- Conception and design: Jeremy Chien and Lisa Neums
- Acquisition of data: Dr. Andrea Mariani
- Analysis and interpretation of data: Lisa Neums, Jeremy Chien, Seiji Suenaga, Devin Koestler and Sara Anders
- Writing, review, and revision of the manuscript: Jeremy Chien, Lisa Neums and Seiji Suenaga
- Administration, technical, or material support: Jeremy Chien, Peter Beyerlein Devin Koestler

Acknowledgements

The results published here are in whole or part based upon data generated by the TCGA Research Network: http://cancergenome.nih.gov/. We acknowledge Dr. Devin Koestler for helpful discussions and comments.

Author details

[1]Department of Cancer Biology, University of Kansas Medical Center, 3901 Rainbow Blvd., 66160 Kansas City, KS, USA. [2]Department of Bioinformatics and Biosystems Technology, University of Applied Sciences Wildau, Hochschulring 1, 15745 Wildau, Germany. [3]Obstetrics and Gynecology, Cancer Center, Mayo Clinic, 200 First St. SW, 55905 Rochester, MN, USA. [4]Department of Biostatistics, University of Kansas Medical Center, 3901 Rainbow Blvd., 66160 Kansas City, KS, USA.

References

1. The Cost of Sequencing a Human Genome. https://www.genome.gov/27565109/the-cost-of-sequencing-a-human-genome/
2. McKenna, A., Hanna, M., Banks, E., Sivachenko, A., Cibulskis, K., Kernytsky, A., Garimella, K., Altshuler, D., Gabriel, S., Daly, M., DePristo, M.A.: The genome analysis toolkit: a mapreduce framework for analyzing next-generation dna sequencing data. Genome Research **20**, 1297–1303 (2010)
3. Fan, Y., Xi, L., Hughes, D.S., Zhang, J., Zhang, J., Futreal, P.A., Wheeler, D.A., Wang, W.: Muse: accounting for tumor heterogeneity using a sample-specific error model improves sensitivity and specificity in mutation calling from sequencing data. Genome Biol. **17**(1), 178 (2016)
4. Larson, D.E., Harris, C.C., Chen, K., Koboldt, D.C., Abbott, T.E., Dooling, D.J., Ley, T.J., Mardis, E.R., Wilson, R.K., Ding, L.: Somaticsniper: identification of somatic point mutations in whole genome sequencing data. Bioinformatics **28**(3), 311–317 (2012)
5. Koboldt, D.C., Zhang, Q., Larson, D.E., Shen, D., McLellan, M.D., Lin, L., Miller, C.A., Mardis, E.R., Ding, L., Wilson, R.K.: Varscan 2: somatic mutation and copy number alteration discovery in cancer by exome sequencing. Genome Research **22**(3), 568–576 (2012)
6. Cai, L., Yuan, W., Zhang, Z.: In-depth comparison of somatic point mutation callers based on different tumor next-generation sequencing depth data. Scientific Reports **36540**(6) (2016)
7. Guettouche, T., Zuchner, S.: Improved coverage and accuracy with strand-conserving sequence enrichment. Genome Med **5**(5), 46 (2013)
8. Parla, J.S., Iossifov, I., Grabill, I., Spector, M.S., Kramer, M., McCombie, W.R.: A comparative analysis of exome capture. Genome Biol **12**(9), 97 (2011)
9. Garcia-Ortega, L.F., Martinez, O.: How many genes are expressed in a transcriptome? estimation and results for rna-seq. PLoS One **10**(6), 0130262 (2015)
10. Shah, S.P., Kobel, M., Senz, J., Morin, R.D., Clarke, B.A., Wiegand, K.C., Leung, G., Zayed, A., Mehl, E., Kalloger, S.E., Sun, M., Giuliany, R., Yorida, E., Jones, S., Varhol, R., Swenerton, K.D., Miller, D., Clement, P.B., Crane, C., Madore, J., Provencher, D., Leung, P., DeFazio, A., Khattra, J., Turashvili, G., Zhao, Y., Zeng, T., Glover, J.N., Vanderhyden, B., Zhao, C., Parkinson, C.A., Jimenez-Linan, M., Bowtell, D.D., Mes-Masson, A.M., Brenton, J.D., Aparicio, S.A., Boyd, N., Hirst, M., Gilks, C.B., Marra, M., Huntsman, D.G.: Mutation of foxl2 in granulosa-cell tumors of the ovary. N Engl J Med **360**(26), 2719–29 (2009)
11. Wiegand, K.C., Shah, S.P., Al-Agha, O.M., Zhao, Y., Tse, K., Zeng, T., Senz, J., McConechy, M.K., Anglesio, M.S., Kalloger, S.E., Yang, W., Heravi-Moussavi, A., Giuliany, R., Chow, C., Fee, J., Zayed, A., Prentice, L., Melnyk, N., Turashvili, G., Delaney, A.D., Madore, J., Yip, S., McPherson, A.W., Ha, G., Bell, L., Fereday, S., Tam, A., Galletta, L., Tonin, P.N., Provencher, D., Miller, D., Jones, S.J., Moore, R.A., Morin, G.B., Oloumi, A., Boyd, N., Aparicio, S.A., Shih Ie, M., Mes-Masson, A.M., Bowtell, D.D., Hirst, M., Gilks, B., Marra, M.A., Huntsman, D.G.: Arid1a mutations in endometriosis-associated ovarian carcinomas. N Engl J Med **363**(16), 1532–43 (2010)
12. Wang, C., Davila, J.I., Baheti, S., Bhagwate, A.V., Wang, X., Kocher, J.P., Slager, S.L., Feldman, A.L., Novak, A.J., Cerhan, J.R., Thompson, E.A., Asmann, Y.W.: Rvboost: Rna-seq variants prioritization using a boosting method. Bioinformatics **30**(23), 3414–3416 (2014)
13. Piskol, R., Ramaswami, G., Li, J.B.: Reliable identification of genomic variants from rna-seq data. Am J Hum Genet **93**(4), 641–651 (2013)
14. Spence, J.M., Spence, J.P., Abumoussa, A., Burack, W.R.: Ultradeep analysis of tumor heterogeneity in regions of somatic hypermutation. Genome Med **7**(1), 24 (2015)
15. Radenbaugh, A.J., Ma, S., Ewing, A., Stuart, J.M., Collisson, E.A., Zhu, J., Haussler, D.: Radia: Rna and dna integrated analysis for somatic mutation detection. PLoS One **9**(11) (2014)
16. Chou, K.C.: Some remarks on protein attribute prediction and pseudo amino acid composition. Journal of theoretical biology **1**(273), 236–47 (2011). doi:10.1016/j.jtbi.2010.12.024
17. Xu, Y., Ding, Y.-X., Ding, J., Lei, Y.-H., Wu, L.-Y., Deng, N.-Y.: isuc-pseaac: predicting lysine succinylation in proteins by incorporating peptide position-specific propensity. Scientific Reports **10184**(5) (2015). doi:10.1038/srep10184
18. cBioPortal for Cancer Genomics. http://www.cbioportal.org/
19. Gao, J., Aksoy, B.A., Dogrusoz, U., Dresdner, G., Gross, B., Sumer, S.O., Sun, Y., Jacobsen, A., Sinha, R., Larsson, E., Cerami, E., Sander, C., Schultz, N.: Integrative analysis of complex cancer genomics and clinical profiles using the cbioportal. Sci Signal **6**(269) (2013). pl1
20. Cerami, E., Gao, J., Dogrusoz, U., Gross, B.E., O, S.S., A, A.B., Jacobsen, A., Byrne, C.J., Heuer, M.L., Larsson, E., Antipin, Y., B, R., Goldberg, A.P., Sander, C., Schultz, N.: The cbio cancer genomics portal: An open platform for exploring multidimensional cancer genomics data. Cancer Discovery **2**(5), 401–404 (2012)
21. Cancer Genomics Hub. https://cghub.ucsc.edu/
22. GDC Data Portal - National Institutes of Health. https://gdc-portal.nci.nih.gov/
23. Wang, I.X., So, E., Devlin, J.L., Zhao, Y., Wu, M., Cheung, V.G.: Adar regulates rna editing, transcript stability, and gene expression. Cell Rep. **5**(3), 849–860 (2013)
24. Blanc, V., Davidson, N.O.: Apobec-1 mediated rna editing. Wiley Interdiscip Rev Syst Biol Med. **2**(5), 594–602 (2011)
25. Blanc, V., Park, E., Schaefer, S., Miller, M., Lin, Y., Kennedy, S., Billing, A.M., Ben Hamidane, H., Graumann, J., Mortazavi, A., Nadeau, J.H., Davidson, N.O.: Genome-wide identification and functional analysis of apobec-1-mediated c-to-u rna editing in mouse small intestine and liver. Genome Biol **15**(6), 79 (2014)
26. McPherson, A., Roth, A., Laks, E., Masud, T., Bashashati, A., Zhang, A.W., Ha, G., Biele, J., Yap, D., Wan, A., Prentice, L.M., Khattra, J., Smith, M.A., Nielsen, C.B., Mullaly, S.C., Kalloger, S., Karnezis, A., Shumansky, K., Siu, C., Rosner, J., Chan, H.L., Ho, J., Melnyk, N., Senz, J., Yang, W., Moore, R., Mungall, A.J., Marra, M.A., Bouchard-Côté, A., Gilks, C.B., Huntsman, D.G., McAlpine, J.N., Aparicio, S., Shah, S.P.: Divergent modes of clonal spread and intraperitoneal mixing in high-grade serous ovarian cancer. Nat Genet. **48**(7), 758–57 (2016). doi:10.1038/ng.3573
27. Birkbak, N.J., Kochupurakkal, B., Izarzugaza, J.M.G., Eklund, A.C., Y, L., Liu, J., Szallasi, Z., Matulonis, U.A., Richardson, A.L., Iglehart, J.D., Wang, Z.C.: Tumor mutation burden forecasts outcome in ovarian cancer with brca1 or brca2 mutations. PLoS ONE **8**(11), 80023 (2013)
28. Tulyakov, S., Jaeger, S., Govindaraju, V., Doermann, D.: Review of Classifier Combination Methods. In: F., S.M.H. (ed.) Studies in Computational Intelligence: Machine Learning in Document Analysis and RecognitionStudies in Computational Intelligence: Machine Learning in Document Analysis and Recognition, pp. 361–386. Springer, New York (2008)
29. Wang, K., Li, M., Hakonarson, H.: Annovar: functional annotation of genetic variants from high-throughput sequencing data. Nucleic Acids Res **38**(16), 164 (2010)
30. Dobin, A., Davis, C.A., Schlesinger, F., Drenkow, J., Zaleski, C., Jha, S., Batut, P., Chaisson, M., Gingeras, T.R.: Star: ultrafast universal rna-seq aligner. Bioinformatics **29**(1), 15–21 (2013)
31. Li, H., Durbin, R.: Fast and accurate long-read alignment with burrows-wheeler transform. Bioinformatics **26**(5), 589–95 (2010)
32. Picard. http://broadinstitute.github.io/picard
33. Li, H.: A statistical framework for snp calling, mutation discovery, association mapping and population genetical parameter estimation from sequencing data. Bioinformatics **27**(21), 2987–93 (2011)
34. Li, H., Handsaker, B., Wysoker, A., Fennell, T., Ruan, J., Homer, N., Marth, G., Abecasis, G., Durbin, R., Genome Project Data Processing, S.: The sequence alignment/map format and samtools. Bioinformatics **25**(16), 2078–9 (2009)
35. Team, R.D.C.: R: A Language and Environment for Statistical Computing. R Foundation for Statistical Computing, Vienna, Austria (2008). R Foundation for Statistical Computing. http://www.R-project.org
36. Quinlan, A.R., Hall, I.M.: Bedtools: a flexible suite of utilities for comparing genomic features. Bioinformatics **26**(6), 841–2 (2010)
37. Ewing, A.D., Houlahan, K.E., Hu, Y., Ellrott, K., Caloian, C., Yamaguchi, T.N., Bare, J.C., P'ng, C., Waggott, D., Sabelnykova, V.Y., participants, I.-T.D.S.M.C.C., Kellen, M.R., Norman, T.C., Haussler, D., Friend, S.H., Stolovitzky, G., Margolin, A.A., Stuart,

J.M., Boutros, P.C.: Combining tumor genome simulation with crowdsourcing to benchmark somatic single-nucleotide-variant detection. Nat Methods **12**(7), 623–30 (2015)
38. Broad Institute's Resource Bundle. ftp://ftp.broadinstitute.org/bundle/2.8/hg19/
39. Wickham, H.: Ggplot2: Elegant Graphics for Data Analysis. Springer, ??? (2009). http://ggplot2.org
40. DePristo, M., Banks, E., Poplin, R., Garimella, K., Maguire, J., Hartl, C., Philippakis, A., del Angel, G., Rivas, M.A., Hanna, M., McKenna, A., Fennell, T., Kernytsky, A., Sivachenko, A., Cibulskis, K., Gabriel, S., Altshuler, D., Daly, M.: A framework for variation discovery and genotyping using next-generation dna sequencing data. NATURE GENETICS **43**, 491–498 (2011)
41. Van der Auwera, G.A., Carneiro, M., Hartl, C., Poplin, R., del Angel, G., Levy-Moonshine, A., Jordan, T., Shakir, K., Roazen, D., Thibault, J., Banks, E., Garimella, K., Altshuler, D., Gabriel, S., DePristo, M.: From fastq data to high-confidence variant calls: The genome analysis toolkit best practices pipeline. CURRENT PROTOCOLS IN BIOINFORMATICS **43**, 11101–111033 (2013)
42. Open Science Framework Repository for VaDiR Data. http://www.osf.io/ap5b7
43. Anders, S., Beyerlein, P., Chien, J., Koestler, D., Mariani, A., Neums, L., Suenaga, S.: Supporting software for "VaDiR: an integrated approach to Variant Detection in RNA" GigaScience Database. http://dx.doi.org/10.5524/100360. VaDiR, RRID:SCR_015797 (2017)

Illustrations and figures

Figure 1 Intersection of the three variant calling methods. (A) Intersection of the three methods with all somatic variants. The red triangles represent the amount of concordant variants. **(B)** Intersection of three methods with only concordant somatic variants. All three callers (Tier1) together has the highest number of condordant variants.

Figure 2 Effect of weighted features on the performance Shown is in panel **A** to **D** the performance of each combinationo of weights of the three callers Haplotypecaller, SNPiR and RVboost while evalue means the sum of FP and FN. The blue point marks the equal combination of all three callers, namly Tier1. In Panel **E** is shown the weights of the callers in each combination.

Figure 3 Variants called in tumor DNA. (A) Percentage of concordant calls of all somatic variants from expressed genes for each sample from two datasets (sensitive and resistant tumor samples). A higher percentage of concordant calls was achieved in transcripts with high expression (DP>10) compared to that of all expressed transcripts (DP>0). **(B)** Violin plot of variant fraction for all somatic variant positions with RNA DP>10. Most of the variant positions missed by VaDiR have a low variant fraction (VAF<0.1) in RNA. **(C)** Ranked SNVs called by TCGA and/or different combinations of RNA-seq calling methods. Only those positions with DP>10 in tumor DNA, RNA and normal DNA are included in the analysis. The names in the chart are the first letters of the caller SNPiR (s), RVBoost (r) and MuTect (m) or their combinations.

Tables and captions

Figure 4 Correlation of variant fractions between RNA and DNA. The four charts show the effect of read depth filter on the correlation of variant fractions.

Figure 5 Comparison of sensitive and resistant samples. (A) Numbers of concordant calls in Tier1 and Tier2 by VaDiR. The precision for each sample for Tier1 and Tier2 is shown in percentage above each bar. **(B)** Number of mutations found at the DNA and RNA level in sensitive tumors are significantly higher than in resistant tumor Samples.

Figure 6 VaDiR workflow for processing somatic variant calls from RNA-seq. Sequence alignment is done by STAR and BWA MEM for RNA and DNA respectively. The refined mapping follows GATK Best Practices. The variant calling is done by Unified Genotyper (GATK) and MuTect2 (GATK). The following filtering steps are done by RVBoost and SNPiR. Additional filters such as MAQ > 40, germline read depth (DP) > 5 and germline variant fraction (VAF) < 0.03 are applied to remove germline variants.

Table 1 Performance characteristics of VaDiR with the combination Tier1.

	DNA positive	DNA negative
RNA positive	452	63
RNA negative	1327	

Table 2 Called spiked-in variants.

Sample	Tier1	Tier2
OV10	68 (54.40%)	78 (62.40%)
OV11	61 (52.59%)	68 (58.62%)
OV12	58 (48.74%)	69 (57.98%)

Percentages represent recall rates in each sample. Tier 1 is the consensus of three callers. Tier 2 is the Tier 1 plus consensus of MuTect2 and SNPiR. Total number of recoverable spiked-in variants is 125 (OV10), 116 (OV11), and 119 (OV12).

Table 3 Characteristics of missed spiked-in variants.

Tier1	OV10	OV11	OV12
all spiked in variants	125	116	119
missed by VaDiR	57	55	61
not called by at least one caller	20	20	20
missed in coding region	16	17	18
missed in coding region by RNA VAF>20%	11	9	13
missed in coding region by RNA VAF>20% and normal DNA DP>10	8	7	11
**Tier2**	**OV10**	**OV11**	**OV12**
all spiked in variants	125	116	119
missed by VaDiR	47	48	50
not called by at least one caller	20	20	20
missed in coding region	9	11	12
missed in coding region by RNA VAF>20%	6	5	9
missed in coding region by RNA VAF>20% and normal DNA DP>10	4	4	8

Click here to download Table
Table1_Performance_characteristics.pdf

Performance characteristics

	DNA positive	DNA negative
RNA positive	452	63
RNA negative	1327	

 Click here to download Table Table2_spiked-in_called.pdf

Sample	Tier1	Tier2
OV10	68 (54.40%)	78 (62.40%)
OV11	61 (52.59%)	68 (58.62%)
OV12	58 (48.74%)	69 (57.98%)

Table 3

Click here to download Table Table3_spiked-in_missed.pdf

Tier1	OV10	OV11	OV12
all spiked in variants	125	116	119
variants found by 1+ caller	105	96	99
Missed by VaDiR	37	36	42
missed in coding region	16	17	18
missed in coding region by RNA VAF>20%	11	9	13
missed in coding region by RNA VAF>20% and normal DNA DP>10	8	7	11
**Tier2**	**OV10**	**OV11**	**OV12**
all spiked in variants	125	116	119
variants found by 1+ caller	105	96	99
missed by VaDiR	27	29	31
missed in coding region	9	11	12
missed in coding region by RNA VAF>20%	6	5	9
missed in coding region by RNA VAF>20% and normal DNA DP>10	4	4	8

Figure 1

Click here to download Figure Figure1_all_srm_somatic.pdf

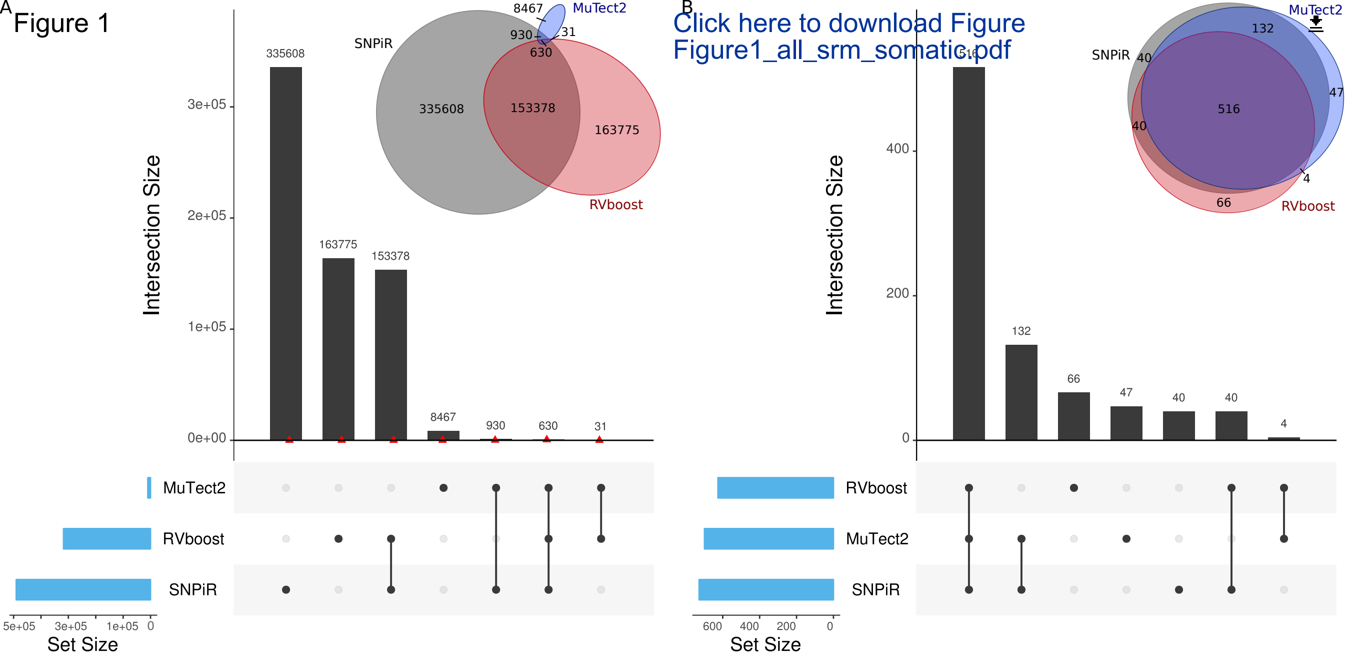

Figure 2

Click here to download Figure

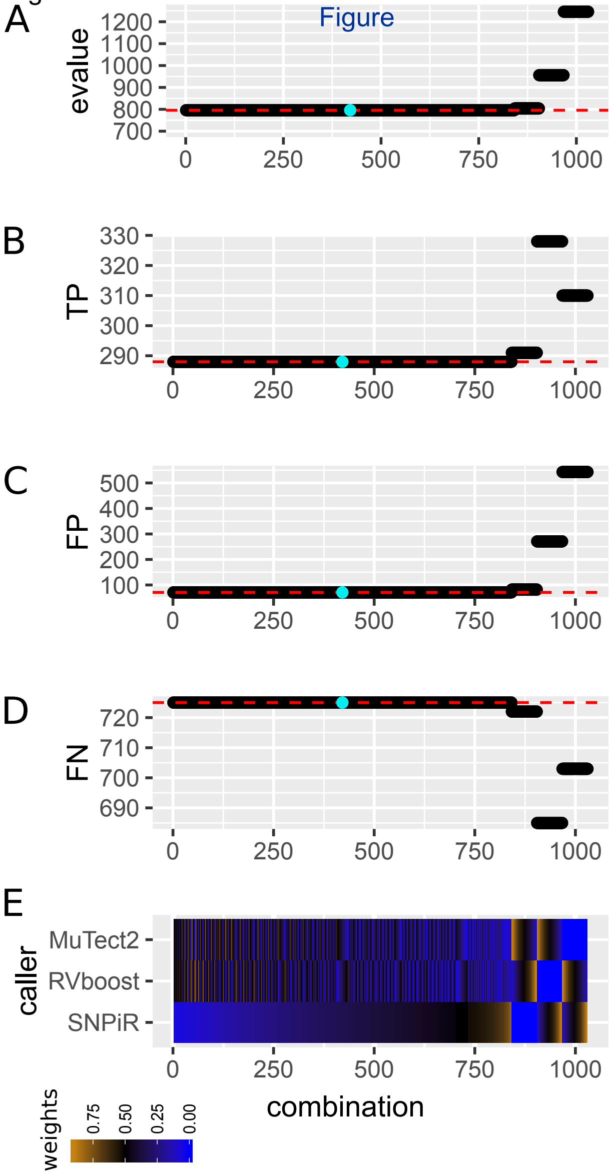

Figure 3

Click here to download Figure Figure3_false_negative_dp5af3c50_Pvalidation.pdf

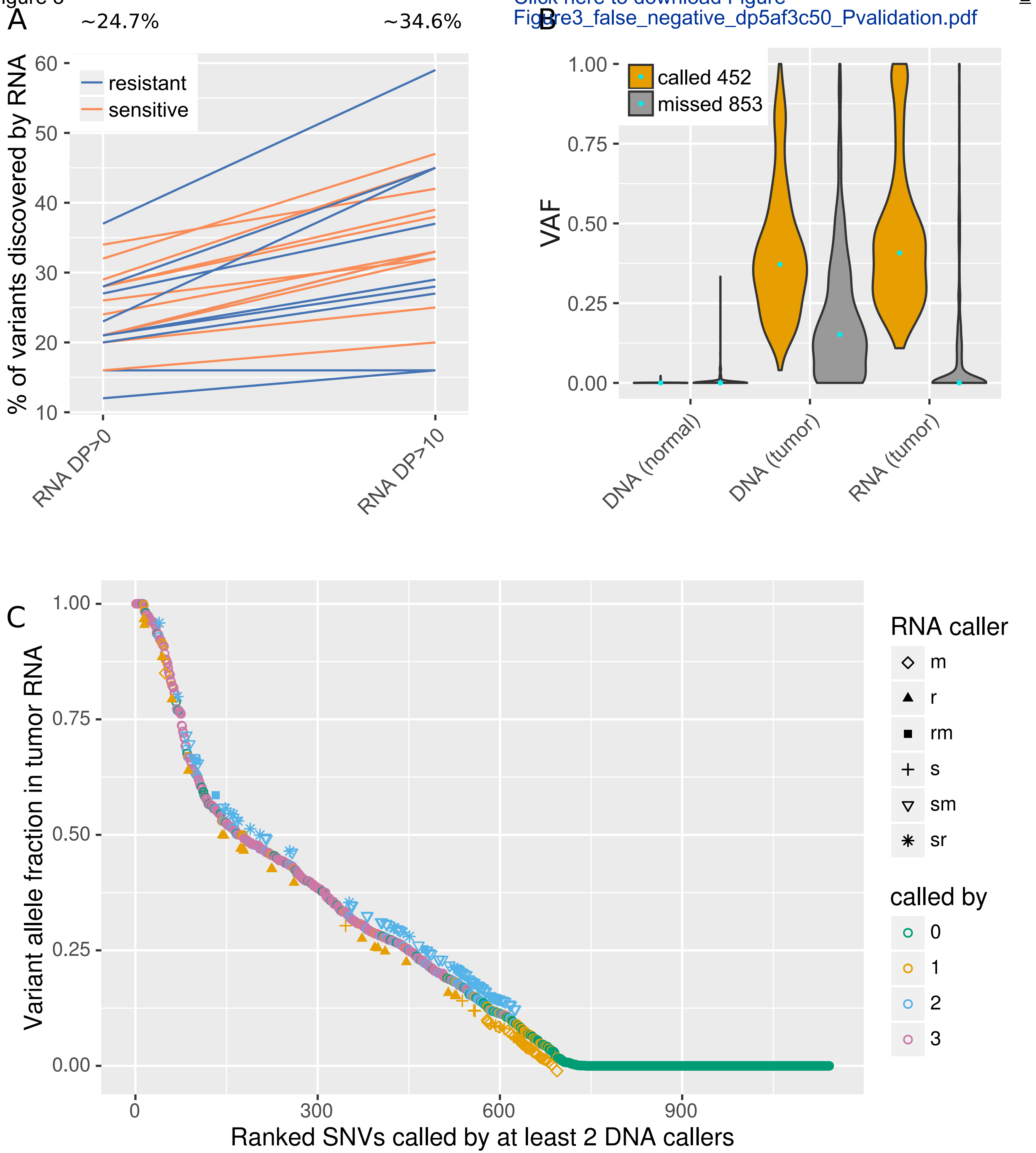

Figure 4

Click here to download Figure Figure4_all_VF_correlation_allDP_c50.pdf

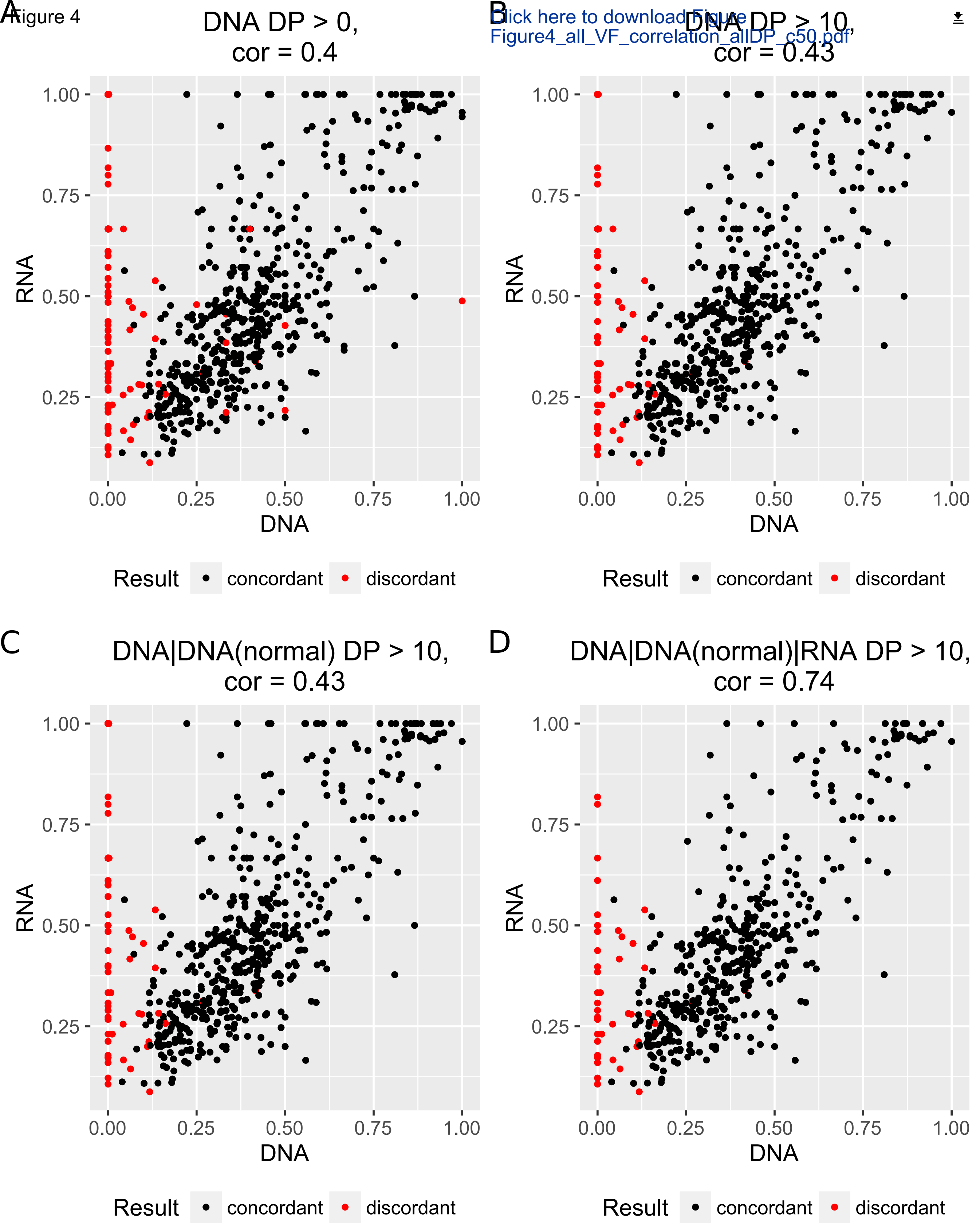

Figure 5

Click here to download Figure Figure5_numbers-resistantVSsensitive_c50.pdf

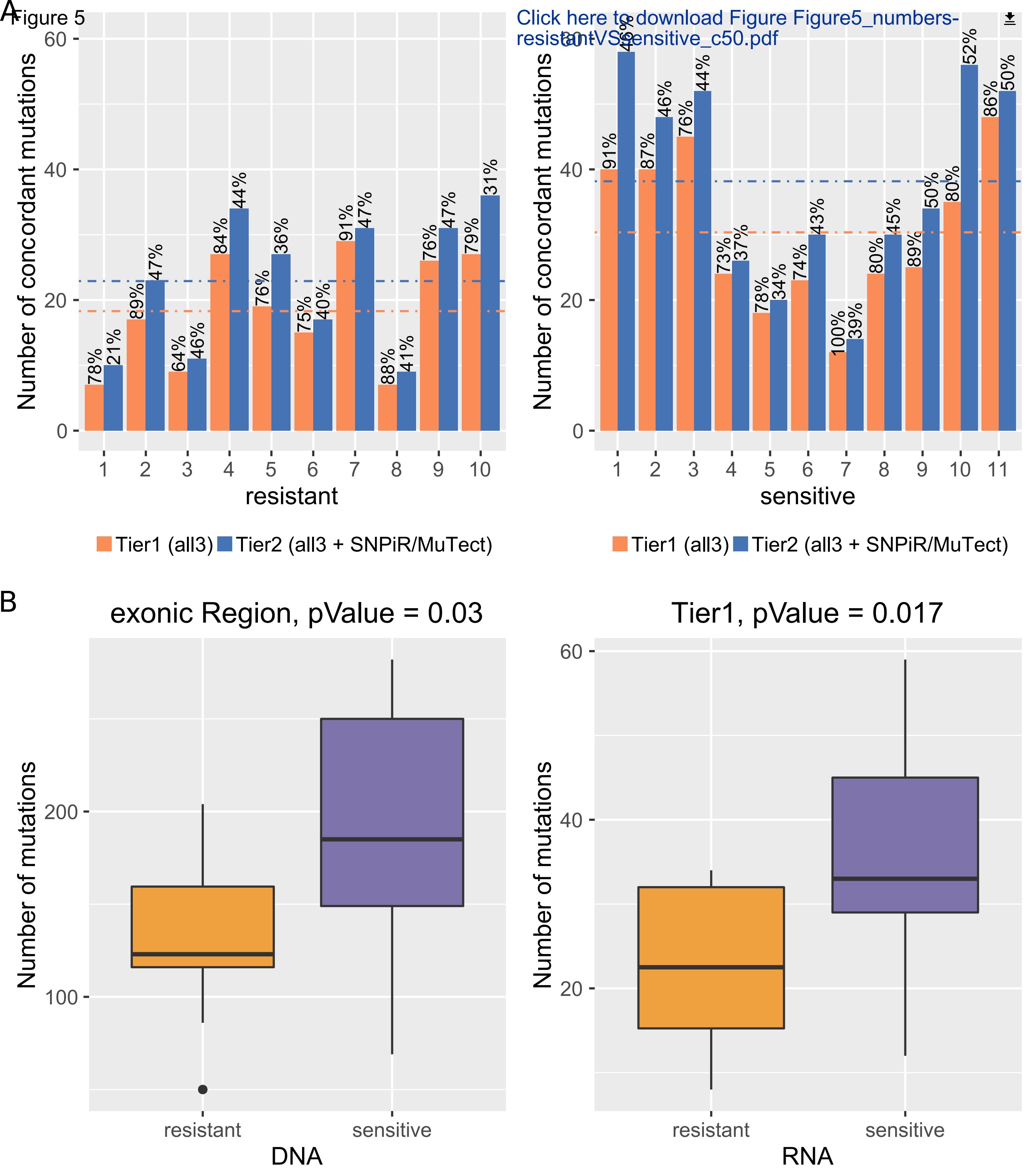

Figure 6

Click here to download Figure Figure6_Calling_methology_VaDiR.pdf

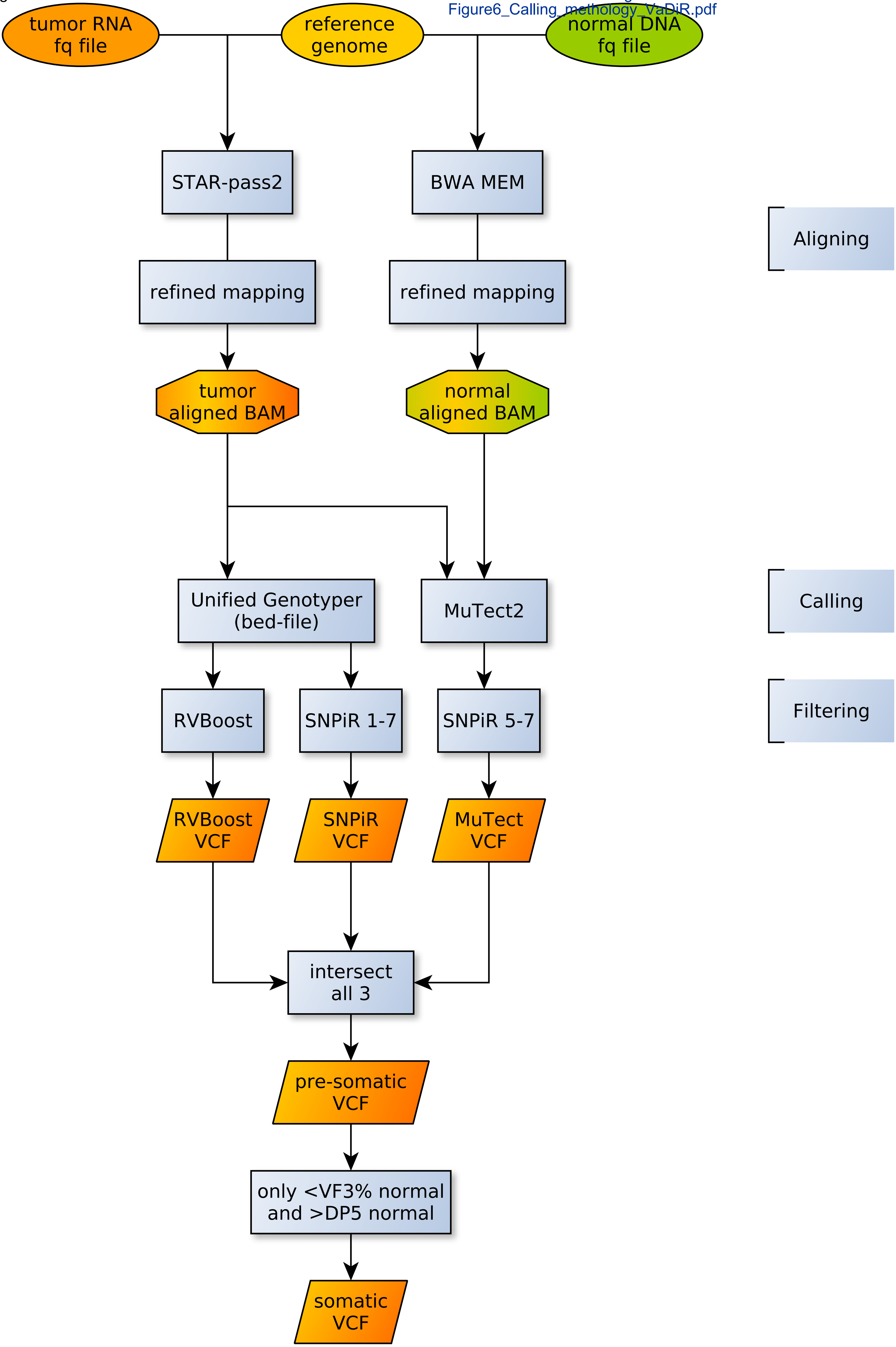

Click here to access/download
Supplementary Material
SupplementaryTable1_Sample_ID_List.pdf

Click here to access/download
Supplementary Material
SupplementaryTable2_performance_callers.pdf

Click here to access/download
Supplementary Material
SupplementaryTable3_used_Software.pdf

Click here to access/download
Supplementary Material
SupplementaryTable4_filtering_of_caller.pdf

Click here to access/download
Supplementary Material
SupplementaryTable5_12samples_weightCombo_unique_sort.txt

Click here to access/download
Supplementary Material
SupplementaryTable6_12samples_weightCombo_vaf_unique_sort.txt

Click here to access/download
Supplementary Material
SupplementaryFigure1_venn-diagram_weighing.pdf

Click here to access/download
Supplementary Material
SupplementaryFigure2_precision_recall_curve_weighting.pdf

Click here to access/download
Supplementary Material
SupplementaryFigure3_missed_timeline.pdf

Click here to access/download
Supplementary Material
SupplementaryFigure4_false_negative_dp5af3c50_missed_pie.pdf

Click here to access/download
Supplementary Material
SupplementaryFigure5_discordant_timeline.pdf

Click here to access/download
Supplementary Material
SupplementaryFigure6_kindMutation_bargraph.pdf

Click here to access/download
Supplementary Material
SupplementaryFigure7_spiked_violin.pdf

Click here to access/download
Supplementary Material
SupplementaryFigure8_radia-violin.pdf

Click here to access/download
Supplementary Material
SupplementaryFigure9_AF-resistantVSsensitive.pdf

Cover letter Click here to download Personal Cover CoverLetter.pdf

UNM COMPREHENSIVE CANCER CENTER

The Official Cancer Center of the State of New Mexico • A Comprehensive Cancer Center Designated by the National Cancer Institute

Hans Zauner
Editor
GigaScience,

Re: GIGA-D-16-00160R2

Dear Dr. Zauner,

Thank you for your suggestions on the revision of our manuscript, titled "VaDiR: an integrated approach to Variant Detection in RNA"

Per your suggestion, we revised the manuscript to include the following:

A reference to the GigaDB entry with DOI.
[43] Neums L, Suenaga S, Beyerlein P, Koestler D, Anders S, Mariani A, Chien J. Supporting software for "VaDiR: an integrated approach to Variant Detection in RNA". 2017. http://dx.doi.org/10.5524/100360

We included the following statement in the "Availability of Supporting Data":
"Supporting data and an archival copy of the code are also available via the GigaScience repository GigaDB [43].
For testing purposes we utilised data kindly provided by Dr. Andrea Mariani of Mayo Clinic, Rochester, Minnesota. Due to ethical constraints these data cannot be shared publicly, but if researchers would like to request access to these data please contact Dr. Andrea Mariani (mariani.andrea@mayo.edu) with a short description of why you require access and how you would use the data."

We updated the manuscript to include the project URL and RRID.

We also included information about third party code in supplemental table 3 (including citation and licence info).

Best regards,

Jeremy Chien

Jeremy Chien, PhD
Endowed Professor in Cancer Genomics and Target Discovery
University of New Mexico Comprehensive Cancer Center
