# Supplementary material for: VaDiR: an integrated approach to Variant Detection in RNA
Source: Gigascience. 2017 Dec 18;7(2):1–13. doi: 10.1093/gigascience/gix122 (PMC5827345; doi:10.1093/gigascience/gix122)
Supplement: Reviewer_3_Original_Submission_(Attachment).pdf [file gix122_reviewer_3_original_submission_(attachment).pdf]

VaDiR: an integrated approach to Variant Detection in RNA.

General comments.

1. The abstract of the paper says (my emphasis)

We investigated the feasibility of uncovering mutations from expressed genes ***using RNA sequencing datasets*** *with a method called "VaDiR: Variant Detection in RNA" that integrate three variant callers, namely: SNPiR, RVBoost and MuTect2.*

What is not stated in the abstract is what we see in Figure 1: the VaDiR pipeline requires a normal DNA fastq file, in addition to a tumor RNA fastq file.

My question. Is VaDiR a pipeline for "uncovering mutations from expressed genes using RNA sequencing datasets", or does it require a normal DNA fastq file as suggested by Figure 1? This is even more puzzling as MuTect2 can be used to call mutations from RNAseq data without matched normal DNA or RNA.

2. Intersecting three mutation-calling methods, each with their own specificity is bound to produce a method whose specificity is as large as the largest of the three specificities. So the fact that the Tier 1 combination leads to a higher percentage of calls validated by DNA is no surprise. The question should then be: what loss in sensitivity has been incurred? The authors note that Tier 2: adding back all MuTect2 and SNPiR calls, "leads to higher sensitivity." Again this is as expected, but they complete this observation by commenting that "the precision is still in a moderate range", and do not mention the magnitude of the inevitable decrease in specificity. Each of the three separate calling methods, and the Tier 1 and Tier 2 combinations will have their own specificity and sensitivity. The authors might like to display all of these using their whole exome sequencing data as truth, and let readers decide. It is usually a trade-off between sensitivity and specificity, though it is not impossible for one method to be best on both criteria.

3. A natural thing to do when combining three callers is to regard the calls as data, and devise a suitable combination of the three that performs better than all three by combining the strengths of all. It seems possible that such a combination would perform better than the Tier 1 and Tier 2 combinations. Is there some reason why the authors did not do this?
