# Supplementary material for: VaDiR: an integrated approach to Variant Detection in RNA
Source: Gigascience. 2017 Dec 18;7(2):1–13. doi: 10.1093/gigascience/gix122 (PMC5827345; doi:10.1093/gigascience/gix122)
Supplement: Supplemental material [file gix122_supp.zip › SupplementaryFigure1_venn-diagram_weighing.pdf]

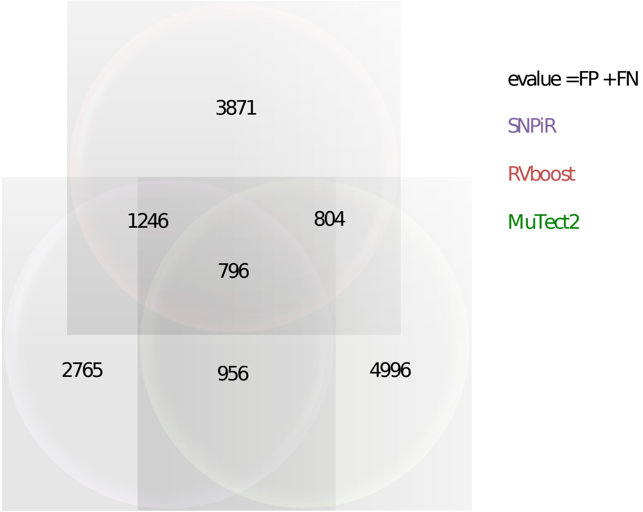

Supplementary Figure 1. Venn-Diagram of evalues of different combinations of callers. Different weighting in one combination of callers leads to the same evalue.
