# Supplementary material for: VaDiR: an integrated approach to Variant Detection in RNA
Source: Gigascience. 2017 Dec 18;7(2):1–13. doi: 10.1093/gigascience/gix122 (PMC5827345; doi:10.1093/gigascience/gix122)
Supplement: Supplemental material [file gix122_supp.zip › SupplementaryFigure2_precision_recall_curve_weighting.pdf]

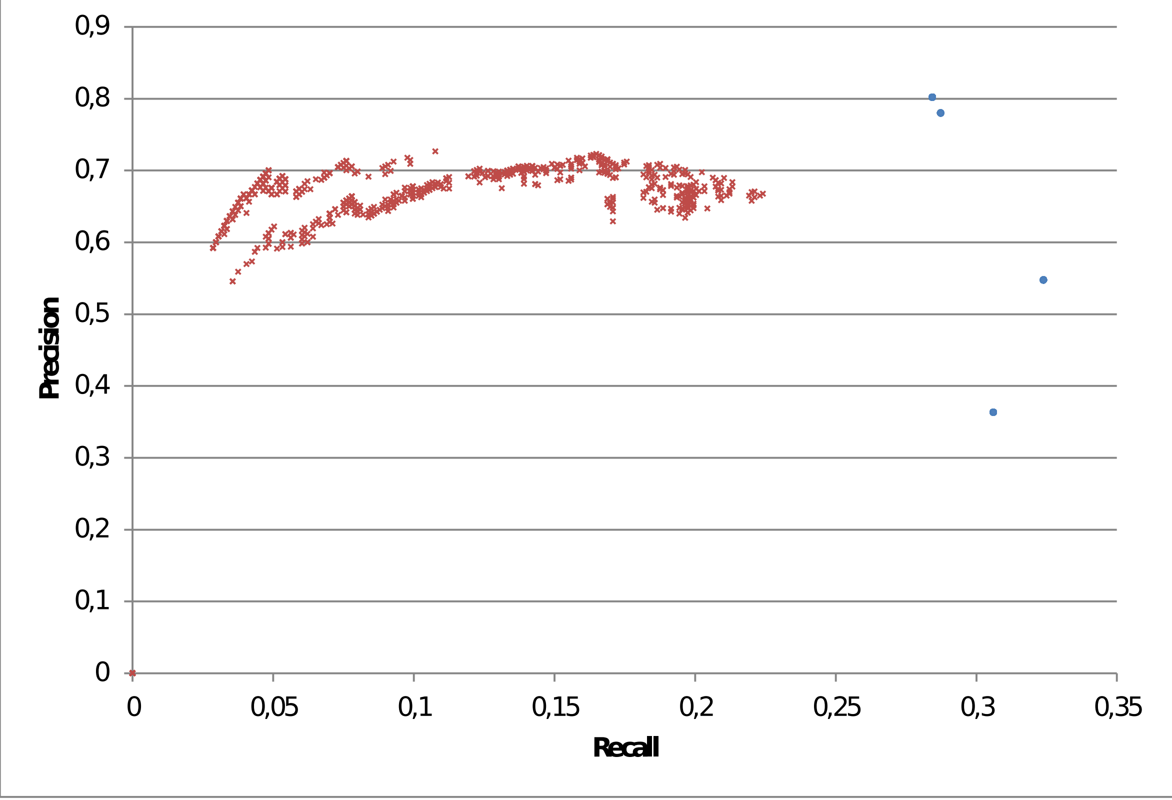

Supplementary Figure 2. Precision-Recall-Curve of the two Experiments with weighting of the callers, where red is Experiment 1 - consider only if the caller has called the variant or not - and blue is Experiment 2 - consider next to the calling the vaf of the called variant. Here we can see that even the best combination of Experiment 2 has a worse recall than each of the combinations of Experiment 2.
