# Supplementary material for: VaDiR: an integrated approach to Variant Detection in RNA
Source: Gigascience. 2017 Dec 18;7(2):1–13. doi: 10.1093/gigascience/gix122 (PMC5827345; doi:10.1093/gigascience/gix122)
Supplement: Supplemental material [file gix122_supp.zip › SupplementaryFigure3_missed_timeline.pdf]

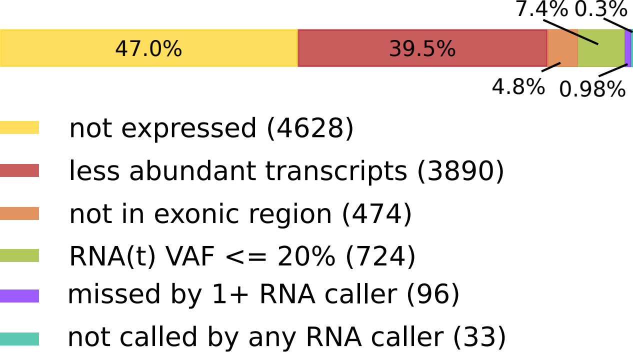

Supplementary Figure 3. Characteristic of all mutations called in tumor DNA by at least two callers, but not by VaDiR. Number of variants are shown in brackets. RNA(t) VAF indicates variant frequency in tumor RNA.
