# Supplementary material for: VaDiR: an integrated approach to Variant Detection in RNA
Source: Gigascience. 2017 Dec 18;7(2):1–13. doi: 10.1093/gigascience/gix122 (PMC5827345; doi:10.1093/gigascience/gix122)
Supplement: Supplemental material [file gix122_supp.zip › SupplementaryFigure4_false_negative_dp5af3c50_missed_pie.pdf]

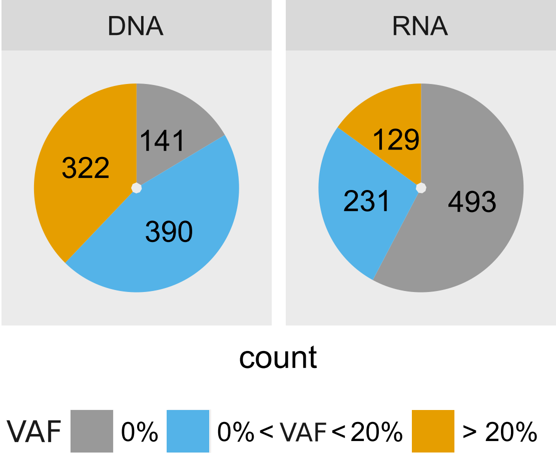

Supplementary Figure 4. Pie chart of number of variants within three specific variant allele frequency (VAF) range produced from SAMtools mpileup with MAQ >= 40. We included all positions called in tumor DNA by at least two callers by TCGA but not by VaDiR with DP>10 in RNA. Those 141 positions with variant frequency = 0 in DNA are the results of filtering out reads with MAQ < 40. These positions may represents false-positive calls by TCGA.
