# Supplementary material for: VaDiR: an integrated approach to Variant Detection in RNA
Source: Gigascience. 2017 Dec 18;7(2):1–13. doi: 10.1093/gigascience/gix122 (PMC5827345; doi:10.1093/gigascience/gix122)
Supplement: Supplemental material [file gix122_supp.zip › SupplementaryFigure5_discordant_timeline.pdf]

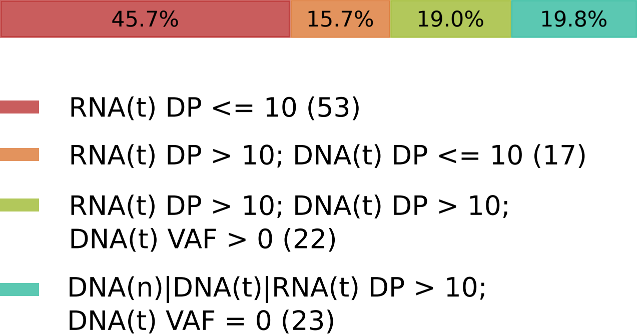

Supplementary Figure 5. Characteristics of all mutations called in RNA by VaDiR but not in tumor DNA by at least two callers. Number of variants are shown in brackets. DP indicates read depth, VAF indicates variant allele frequency, and (t) and (n) indicate tumor and normal respectively.
