# Supplementary material for: VaDiR: an integrated approach to Variant Detection in RNA
Source: Gigascience. 2017 Dec 18;7(2):1–13. doi: 10.1093/gigascience/gix122 (PMC5827345; doi:10.1093/gigascience/gix122)
Supplement: Supplemental material [file gix122_supp.zip › SupplementaryFigure6_kindMutation_bargraph.pdf]

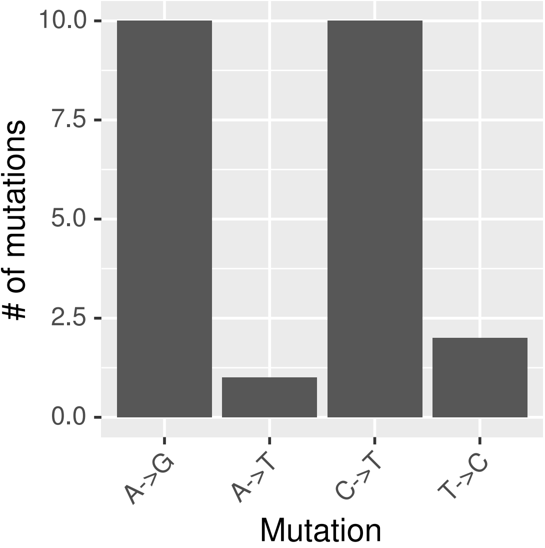

Supplementary Figure 6. Numbers of specific types of mutations with variant allele frequency = 0 in DNA and DP>10 in tumor DNA, RNA and normal DNA. Note that out of 10 A>G variants, 1 is recurrent in 4 samples. Out of 10 C>T variants, 1 is recurrent in 9 samples. These sites likely represents novel RNA-editing sites.
