# Supplementary material for: VaDiR: an integrated approach to Variant Detection in RNA
Source: Gigascience. 2017 Dec 18;7(2):1–13. doi: 10.1093/gigascience/gix122 (PMC5827345; doi:10.1093/gigascience/gix122)
Supplement: Supplemental material [file gix122_supp.zip › SupplementaryFigure7_spiked_violin.pdf]

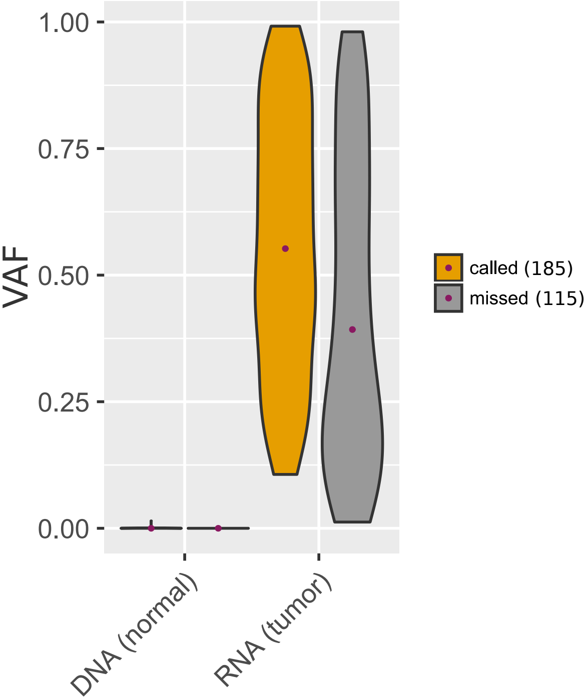

Supplementary Figure 7. Violin plot of variant allele fraction (VAF) of spiked-in variants. Called and missed variants are shown in different colors. Number of variants are shown in brackets
