# Supplementary material for: VaDiR: an integrated approach to Variant Detection in RNA
Source: Gigascience. 2017 Dec 18;7(2):1–13. doi: 10.1093/gigascience/gix122 (PMC5827345; doi:10.1093/gigascience/gix122)
Supplement: Supplemental material [file gix122_supp.zip › SupplementaryFigure8_radia-violin.pdf]

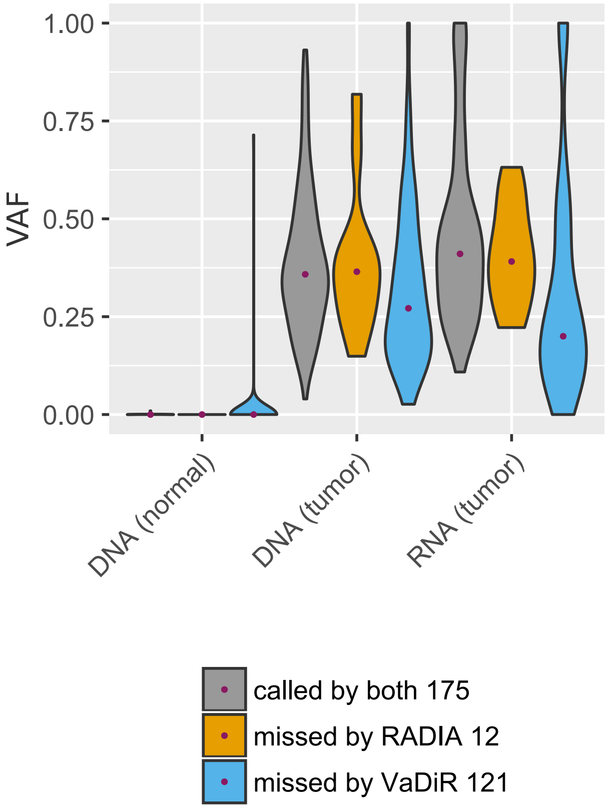

Supplementary Figure 8. Violin plot of variant allele fraction of calls made by RADIA or VaDiR in 6 TCGA samples. Note that most of the variants missed by VaDiR has low vaf whereas those missed by RADIA has high variant fraction.
