# Supplementary material for: VaDiR: an integrated approach to Variant Detection in RNA
Source: Gigascience. 2017 Dec 18;7(2):1–13. doi: 10.1093/gigascience/gix122 (PMC5827345; doi:10.1093/gigascience/gix122)
Supplement: Supplemental material [file gix122_supp.zip › SupplementaryFigure9_AF-resistantVSsensitive.pdf]

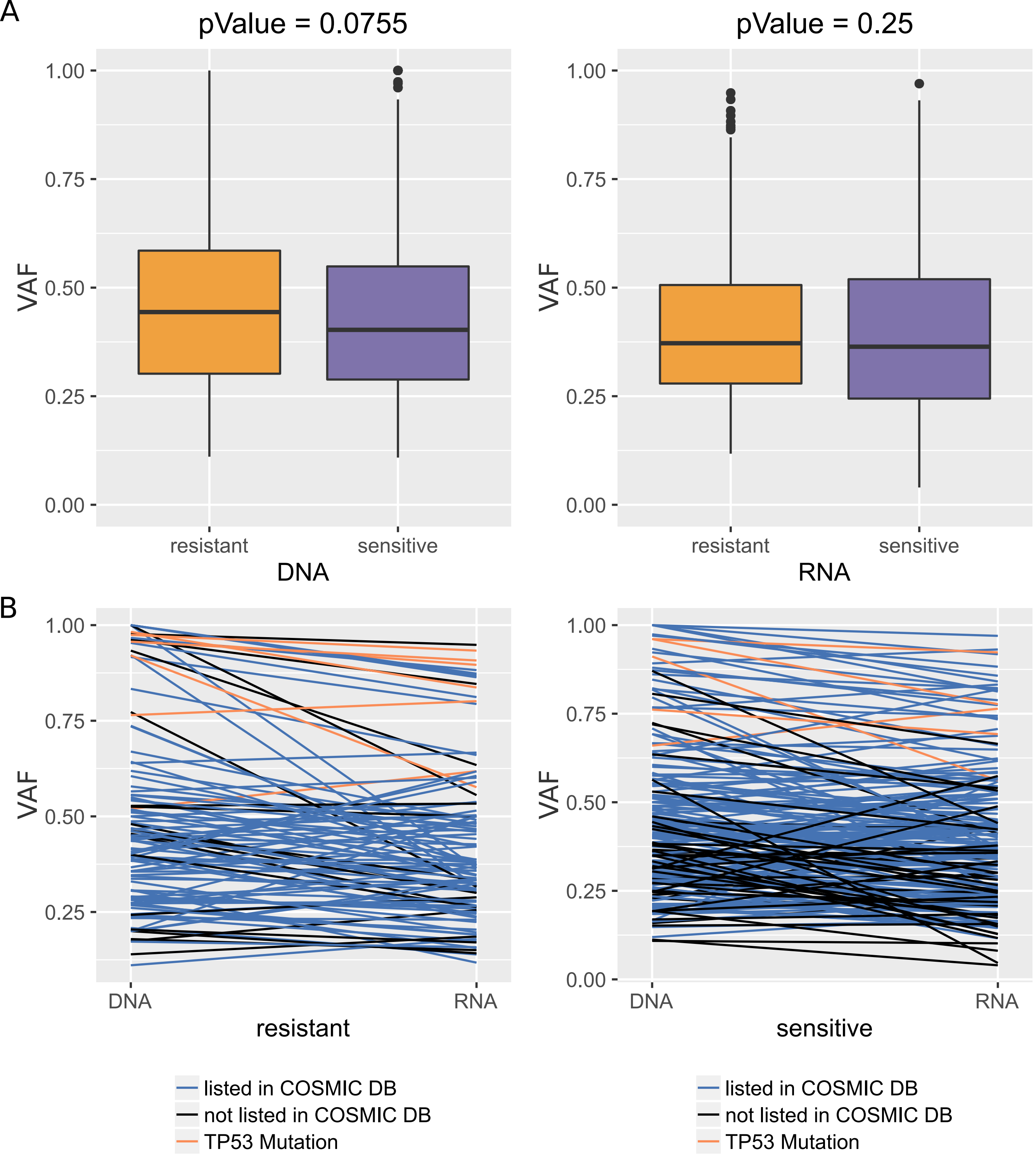

Supplementary Figure 9. Variant fraction of nonsynonymous SNVs in ovarian tumor samples.

(A) Variant allele fraction of SNVs in DNA and RNA are not siginificantly different between sensitive and resistant tumor samples.

(B) Most of the variants have similiar variant allele fraction between DNA and RNA.
