# Supplementary material for: VaDiR: an integrated approach to Variant Detection in RNA
Source: Gigascience. 2017 Dec 18;7(2):1–13. doi: 10.1093/gigascience/gix122 (PMC5827345; doi:10.1093/gigascience/gix122)
Supplement: Supplemental material [file gix122_supp.zip › SupplementaryTable1_Sample_ID_List.pdf]

Supplementary Table 1

Supplementary Table 1. Sample Ids for TCGA samples used in the study

TCGA-ID	GDC-case ID	DNA tumor BAM (gdc ID)	DNA normal BAM (gdc ID)	RNA tumor BAM (cghub ID)	RNA tumor BAM (cghub sample ID)
resistant					
TCGA-24-0970	95e58015-a7c0-4bdc-bf64-7617d24d0784	0911a991-62b5-435f-98ae-7c30fe176cd8	7e280f43-751a-43ce-adc3-2730b06c5aab	0bba3249-a438-4d77-a4cf-555d0e41d5fb	D0URAACXX_7_TACAGC
TCGA-24-1434	5503b3a1-7d03-41b2-9ec8-e478c5414d67	0387b473-1d69-4c97-8996-de9c5225f909	fc91c618-5b4e-4885-8b46-b2c05c1e0044	0eb605ba-036f-48f0-8a4e-1616407c3192	D0RB8ACXX_5_CCACGC
TCGA-13-0724	a9abe7a7-4126-414b-87d2-a6d25abcf1fa	0283ee7f-4901-482e-8588-3a7a5749f5dd	2ee94539-009a-4d3c-9423-ee372d426201	13668db0-12e4-406b-ba42-09f1099161dc	D0DWTACXX_3_AAAGCA
TCGA-13-1483	611600a6-43ec-4029-9682-cd6d6a3312ec	fa4bf71d-dad0-4b4f-95ac-7cbff9d91470	629b79eb-56c5-4878-a820-95cf6a40541f	1496fdd7-65a5-4b56-ae52-e171f305f1d7	C0L8DACXX_3_TGAATG
TCGA-10-0931	e07a61e2-bcd2-4a96-80df-97e04aafbd32	15248f65-b68b-41d0-b902-8eb42e76ee21	cb5ef26c-543c-459c-bf26-f1e7231f9eef	1fd0c6c8-cad5-45e6-ad7b-bfdad7b57faf	D0DWEACXX_2_AACTTG
TCGA-24-1464	4160e048-f0b0-40f5-805b-e277a5893a3b	9e03d2e2-d0b1-4c80-89aa-bd8fa64409c5	b4b9bfa7-eaf6-4101-9abe-3c10aa16a561	247feb7d-4e8e-4d50-a33b-950735993008	C0W1YACXX_3_TGACCA
TCGA-29-1696	8a98a6e6-b763-4824-858b-fd2738e6c9a3	e971f88a-7105-4d03-a76b-2dc00ba800d3	61f1f15c-89b9-4eb6-89ce-2778bfe61f45	29745979-a76b-4351-a8d8-b5f982a20705	D0W3BACXX_8_CAGGCG
TCGA-10-0926	e641aed9-1dd8-4c30-b231-f12b20a76df0	c7b113e3-0fcb-470a-8d9a-7e05c3a683b6	d222d6a4-0b27-4635-9940-e21e89e41245	2f9595fd-c47b-495c-bd91-cb04712587cc	D0ALPACXX_1_GATGCT
TCGA-09-0369	1d9893c8-0de3-4b07-b0ba-a53019b23eb4	6e9a3a0c-f8d6-4ed4-87ca-aab801d96b3f	3d341611-e668-413c-940a-92fcafce635e	3430afc7-6b2a-49c0-b37a-b1e57a987160	C088AACXX_3_CCAACA
TCGA-25-1315	635f5335-b008-428e-b005-615776a6643f	1948ef01-9d57-4880-b692-a5f1aab9759f	d57f7ca3-a936-4ecb-9aff-2b509dfbe6c9	36e131ad-d368-4421-bd3a-9769c853679f	D0URAACXX_4_GCACTT
sensitive					
TCGA-20-1683	ac2e88ff-8b1e-4691-9a96-5a581f98d827	566a6514-acb6-4f20-ba90-eb591ecbab56	3413fcbd-28ca-460d-8f7c-a39ea588436f	240a6232-97bc-4df2-adcf-799b1d7f25fb	D0REMACXX_4_GCCTTA
TCGA-29-1693	6fb71a0c-bc50-48c8-acfa-db94be1e151a	6d301032-ef19-4f48-8139-9fc668ba13ab	279ea305-b34e-4169-b3dd-8e96ec56425c	2b66c6fc-2024-4591-ad7f-14604ad6c414	D0VYMACXX_4_AAACAT
TCGA-13-1497	bc4bc342-20bf-40c3-af26-2c6f942da93d	ec83878e-dee2-4933-a09c-84bda5ad381a	df3e8672-f224-477f-a9d5-101f6f279bc6	4039d5ca-7ad4-40b7-8898-9435b99d213d	C0L6EACXX_7_ATAATT
TCGA-13-1489	7248cd60-be22-44bc-bc58-f644db0940a2	7d5f4c7a-41d6-4041-924e-acae9541a794	d7fd002e-2007-4e2e-9447-bcb58f741222	4272c000-485e-4f16-abff-203c4cf1988e	D0ULKACXX_1_CAGGCG
TCGA-61-1736	0a2d29de-869a-4dc8-ad11-6ee0d0a3a895	c907ba78-903d-4462-ad36-57560bc259be	6f9b430f-391d-4c2e-b603-5a85dd0b01f5	5422975a-635b-4196-9a28-e17eb47910e8	D0W1KACXX_3_AGGTTT
TCGA-29-1769	94769f2c-b6fd-48ec-af34-b41165340b7c	478841a9-9602-4435-8ca7-f51e190314fb	39459643-e5a0-4efd-bade-830a2aeb68cd	5e750b4c-5d47-4f61-8c0e-2a1683f9bb2a	D0W3BACXX_7_AAGACT
TCGA-13-0916	5f60bc2d-738f-43fc-a3fb-61ec6e80e3d4	75bd5eab-b827-4848-9e6d-86ed1f68cb8d	e7ff609d-9472-410d-bacb-8af3925d358a	66364a2d-39ea-4490-a9d0-ab9eaeb353f8	D0ALPACXX_2_GCCGCG
TCGA-24-1551	499a9b57-ee1a-4012-bbab-c6ad955b5e0a	1b62d8ce-fc08-48e2-a634-07f220d2d0ee	2195afaf-06e7-4fe3-9511-5185a733e7ab	7767355e-52d9-4016-b57d-bf571e0f7d7c	D0RMBACXX_4_GAGTGG
TCGA-24-1103	f34aa3b6-e966-49c6-bc55-130545772c53	fa48db21-e7eb-46ab-9d47-f186317c85aa	079572c4-59d2-409f-b0b4-a4c2913e6ab2	877d516e-b9ee-4056-8e7b-feafaca0341d	C0L6EACXX_4_ACGATA
TCGA-23-1114	c0c3caab-9277-4a31-a96c-c607e38d5ccc	039411de-98b0-4e08-a03c-aa75ba981a49	9c696325-5716-4bba-bddd-f1c2ff8cbbff	988ecbd2-252d-4621-8294-3795a33a2e70	D0RB8ACXX_3_ATCTAT
TCGA-23-2084	2cb82948-7cbe-4b6c-8414-c02f662de2d0	6ad58e0a-78aa-471e-bb80-6788ac2daf00	5c132a84-689f-4af9-b2a1-2456682a21fd	affc5de3-8a2f-4b03-b20a-bbc155986783	C0NWCACXX_1_CAGGCG
