# Supplementary material for: VaDiR: an integrated approach to Variant Detection in RNA
Source: Gigascience. 2017 Dec 18;7(2):1–13. doi: 10.1093/gigascience/gix122 (PMC5827345; doi:10.1093/gigascience/gix122)
Supplement: Supplemental material [file gix122_supp.zip › SupplementaryTable2_performance_callers.pdf]

Supplementary Table 2. Performance of the calling tools and specific combinations of the tools.

	SNPiR	Rvboost	MuTect2	Tier1	Tier2
TP	728	626	699	516	648
FP	489,818	317,188	9,359	116	912
FN	5,005	5,107	5,034	5,217	5,085
recall	0.126984127	0.1091923949	0.1219256934	0.0900052329	0.1130298273
precision	0.0014840606	0.0019697056	0.0694969179	0.8164556962	0.4153846154
