# Supplementary material for: VaDiR: an integrated approach to Variant Detection in RNA
Source: Gigascience. 2017 Dec 18;7(2):1–13. doi: 10.1093/gigascience/gix122 (PMC5827345; doi:10.1093/gigascience/gix122)
Supplement: Supplemental material [file gix122_supp.zip › SupplementaryTable3_used_Software.pdf]

Supplementary Table 3. Software used in the study.

Software	Version
Annovar	2015Mar22
BAMSurgeon	1
BCFtools	1.3.1
BEDtools	2.25.0
BWA MEM	0.7.15
GATK	3.6-0-g89b7209
Java	1.8.0_66
PBLAT	1.6
Perl	v5.18.2
Picard	2.1.1
Python	2.7
R	3.3.0
RADIA	1
RVboost	0.1
SAMtools	1.2
SNPiR	20140512 + custom revisions
STAR	2.5.2a
STAR code for long-rna-seq	we modified the script to suit our needs
GATK best practises	we modified the script to suit our needs

Licence
ANNOVAR is freely available to personal, academic and non-profit use only. (http://www.openbioinformatics.org/annovar/annovar_download_form.php)
open source (https://github.com/adamewing/bamsurgeon/blob/master/LICENSE.txt)
MIT/Expat license (https://github.com/samtools/bcftools/blob/develop/LICENSE)
GNU GENERAL PUBLIC LICENSE (https://github.com/arq5x/bedtools/blob/master/LICENSE)
open source (https://github.com/lh3/bwa)
FOR ACADEMIC NON-COMMERCIAL RESEARCH PURPOSES ONLY (https://software.broadinstitute.org/gatk/download/licensing.php)
open source
The source code and executables are freely available for academic, nonprofit and personal use. (http://icebert.github.io/pblat/)
open source
open-source under the MIT license (http://broadinstitute.github.io/picard/)
open source
open source (https://www.r-project.org/Licenses/)
GNU Affero General Public License (https://github.com/aradenbaugh/radia)
open source (http://bioinformaticstools.mayo.edu/research/rvboost/)
CC BY-NC-ND 4.0 (http://www.htslib.org/terms/)
MIT License (https://github.com/meissnert/SNPiR/blob/master/LICENSE)
open source (https://github.com/alexdobin/STAR/blob/master/LICENSE)
MIT License (https://github.com/ENCODE-DCC/long-rna-seq-pipeline/blob/master/LICENSE.txt)
open source

**Citation**
Wang, K., Li, M., Hakonarson, H.: Annovar: functional annotation of genetic variants from high-throughput sequencing data. Nucleic Acids Res 38(16), 164 (2010)
Ewing, A.D., Houlahan, K.E., Hu, Y., Ellrott, K., Caloian, C., Yamaguchi, T.N., Bare, J.C., P'ng, C., Waggott, D., Sabelnykova, V.Y., participants, I.-T.D.S.M.C.C., Kellen, M.R., Norman, T.C., Haussler, D., Friend, S.H., Stolovitzky, G., Margolin, A.A., Stuart, J.M., Boutros, P.C.: Combining tumor genome simulation with crowdsourcing to benchmark somatic single-nucleotide-variant detection. Nat Methods 12(7), 623{30 (2015)
Li, H.: A statistical framework for snp calling, mutation discovery, association mapping and population genetical parameter estimation from sequencing data. Bioinformatics 27(21), 2987{93 (2011); Li, H., Handsaker, B., Wysoker, A., Fennell, T., Ruan, J., Homer, N., Marth, G., Abecasis, G., Durbin, R., Genome Project Data Processing, S.: The sequence alignment/map format and samtools. Bioinformatics 25(16), 2078{9 (2009)
Quinlan, A.R., Hall, I.M.: Bedtools: a exible suite of utilities for comparing genomic features. Bioinformatics 26(6), 841{2 (2010)
Li, H., Durbin, R.: Fast and accurate long-read alignment with burrows-wheeler transform. Bioinformatics 26(5), 589{95 (2010)
McKenna, A., Hanna, M., Banks, E., Sivachenko, A., Cibulskis, K., Kernytsky, A., Garimella, K., Altshuler, D., Gabriel, S., Daly, M., DePristo, M.A.: The genome analysis toolkit: a mapreduce framework for analyzing next-generation dna sequencing data. Genome Research 20, 1297{1303 (2010)
Copyright (C) 2012 - 2017 Wang Meng
Picard. http://broadinstitute.github.io/picard
Team, R.D.C.: R: A Language and Environment for Statistical Computing. R Foundation for Statistical Computing, Vienna, Austria (2008). R Foundation for Statistical Computing. http://www.R-project.org
Radenbaugh, A.J., Ma, S., Ewing, A., Stuart, J.M., Collisson, E.A., Zhu, J., Haussler, D.: Radia: Rna and dna integrated analysis for somatic mutation detection. PLoS One 9(11) (2014)
Wang, C., Davila, J.I., Baheti, S., Bhagwate, A.V., Wang, X., Kocher, J.P., Slager, S.L., Feldman, A.L., Novak, A.J., Cerhan, J.R., Thompson, E.A., Asmann, Y.W.: Rvboost: Rna-seq variants prioritization using a boosting method. Bioinformatics 30(23), 3414{3416 (2014)
Li, H.: A statistical framework for snp calling, mutation discovery, association mapping and population genetical parameter estimation from sequencing data. Bioinformatics 27(21), 2987{93 (2011); Li, H., Handsaker, B., Wysoker, A., Fennell, T., Ruan, J., Homer, N., Marth, G., Abecasis, G., Durbin, R., Genome Project Data Processing, S.: The sequence alignment/map format and samtools. Bioinformatics 25(16), 2078{9 (2009)
Piskol, R., Ramaswami, G., Li, J.B.: Reliable identification of genomic variants from rna-seq data. Am J Hum Genet 93(4), 641{651 (2013)
Dobin, A., Davis, C.A., Schlesinger, F., Drenkow, J., Zaleski, C., Jha, S., Batut, P., Chaisson, M., Gingeras, T.R.: Star: ultrafast universal rna-seq aligner. Bioinformatics 29(1), 15{21 (2013)
Colin Dewey, Alex Dobin, https://github.com/ENCODE-DCC/long-rna-seq-pipeline/blob/master/DAC/STAR_RSEM.sh
https://software.broadinstitute.org/gatk/best-practices/
