# Supplementary material for: VaDiR: an integrated approach to Variant Detection in RNA
Source: Gigascience. 2017 Dec 18;7(2):1–13. doi: 10.1093/gigascience/gix122 (PMC5827345; doi:10.1093/gigascience/gix122)
Supplement: Supplemental material [file gix122_supp.zip › SupplementaryTable4_filtering_of_caller.pdf]

Supplementary Table 4. Filtering steps.

**Rvboost**
Adding Distance to Junction to the VCF file from RNA BAM file
Adding Edit Distance (blat field) to the VCF file from RNA BAM file
Adding Coding/NonCoding Flag to the VCF file from RNA BAM file
Run snpefff on the VCF file
Add RNA editing columns to the INFO fields
add TRAIN flag to the VCF file
Filtering variants with RVboost QScore < 0.05
**SNPiR**
SNPiR - Converting VCF to custom SNPiR format & filtering out variants with quality < 20
SNPiR - Filtering out mismatches in first 6 bp of reads
SNPiR - Using BEDtools subtract to remove sites in repetitive regions based on RepeatMasker annotation
SNPiR - Filtering intronic candidates within 4 bp of splicing junctions
SNPiR - Filtering candidates in homopolymer runs
SNPiR - Using PBLAT to ensure unique mapping
SNPiR - Using BEDtools subtract to filter out known RNA editing sites
**MuTect2**
MuTect2 - Evidence seen in the normal sample
MuTect2 - Clustered events observed in the tumor
MuTect2 - Evidence indicates this site is germline, not somatic
MuTect2 - More than three events were observed in the tumor (homologous mapping)
MuTect2 - Multiple events observed in tumor and normal
MuTect2 - Site filtered due to contraction of short tandem repeat region
MuTect2 - Tumor does not meet likelihood threshold
MuTect2 - Site filtered because more than two alt alleles pass tumor LOD
SNPiR - Filtering intronic candidates within 4 bp of splicing junctions
SNPiR - Using PBLAT to ensure unique mapping
SNPiR - Using BEDtools subtract to filter out known RNA editing sites
