# Supplementary material for: VaDiR: an integrated approach to Variant Detection in RNA
Source: Gigascience. 2017 Dec 18;7(2):1–13. doi: 10.1093/gigascience/gix122 (PMC5827345; doi:10.1093/gigascience/gix122)
Supplement: Response_to_Reviewer_Comments_Original_Submission.pdf [file gix122_response_to_reviewer_comments_original_submission.pdf]

Author's Response To Reviewer Comments

Point by point

We thanked the reviewers for wonderful suggestions and constructive critiques.

Reviewer 1:
(1) To make the structure of this paper logically more clear and practically more useful, the authors should in the end of the Introduction (or right before the beginning of describing their own method) add a prelude, such as: "As demonstrated by a series of recent publications [1-7] in compliance with the 5-step rule [8], to establish a really useful sequence-based statistical predictor for a biological system, we should follow the following five guidelines: (a) construct or select a valid benchmark dataset to train and test the predictor; (b) formulate the biological sequence samples with an effective mathematical expression that can truly reflect their intrinsic correlation with the target to be predicted; (c) introduce or develop a powerful algorithm (or engine) to operate the prediction; (d) properly perform cross-validation tests to objectively evaluate the anticipated accuracy of the predictor; (e) establish a user-friendly web-server for the predictor that is
accessible to the public. Below, we are to describe how to deal with these steps one-by- one." With such a prelude, the outline of this paper and its aim would be crystal clear.

We included a prelude to describe the following explanations in the paper:

“In this study, following the recommendation and practices that are widely adopted in the field of bioinformatics [cite: PMID: 21168420; PMID: 26084794], we chose a validated dataset to perform a detailed comparison of somatic DNA and somatic RNA sequence variations from 21 pairs of whole exome and mRNA sequencing from ovarian cancer genomes. We formulated an approach to utilize three publicly available tools, namely MuTect2, RVboost and SNPiR for variant discovery from RNA sequencing. We evaluated the performance of each tool and established the best combination of these tools that enables discovery of variants from RNA sequence with high precision and recall. We showed that most of the variants which would be classified as false-positives or false-negatives can be explained by biological characteristics. In addition, we investigated the performance of our workflow on artificially spiked variants in coding regions of mRNA sequencing data and we compared the performance of VaDiR to RADIA. Finally, we showed the performance of our workflow on a biologically relevant study: the comparison of variants from resistant and sensitive patients to the treatment against high serous ovarian carcinoma.”

(2) Recently, some very powerful bioinformatics tools for analyzing DNA/RNA sequences have been developed [9-14]. The authors should explicitly mention these powerful tools to provide the readership with an updated background and rapid development in this area. The authors should also mentioned a recent paper [15] in the context relevant to the NGS (next-generation sequencing).

The mentioned tools (Ref. 9-14) are not directly relevant to our workflow, and therefore we did not include the citation. The mentioned paper (Cai, L.; Yuan, W.; Zhang, Z. In-depth comparison

of somatic point mutation callers based on different tumor next-generation sequencing depth data Scientific Reports, 2016, 6, 36540.) was cited in context:

“Sequencing only exonic regions of the genome helps reduce cost, and multiple tools (such as MuTect2 provided by GATK [2], MuSE [3], SomaticSniper [4] and VarScan2 [5]) have been developed for somatic variant discovery using whole exome sequencing (WES) data, and the performance of these tools was recently evaluated [6].”

(3) It is pity that the authors did not provide a web-server for their new method of VaDiR presented in this paper. To attract the readership to their future work and to the GigaScience journal as well, the authors should add a discussion in the end of their paper, such as: "As demonstrated in a series of recent publications (see, e.g., [2,3,5,7,16-22]) in developing new prediction or detection methods, user-friendly and publicly accessible web-servers will significantly enhance their impacts [23], we shall make efforts in our future work to provide a web-server for the detection method reported in this paper."

We agreed with the reviewer and the mission of GigaScience journal that developed tools that are publicly accessible through web servers will significantly enhance the impacts of the study and publication. Therefore, we are committed to efforts in the future to provide such capability.

Reviewer 2:

(1) Methods:
a. The authors simply computed the overlap of the variant calls from three methods, SNPiR, RVBoost, and MuTect2.
Therefore, the census calls could be very sensitive to the results of the three algorithms.
The authors also noticed that some variations with high expression and high variant allele frequencies were either not called by any of the three methods or were filtered out by at least one of the three methods.
A more principled way to combine the outputs of various algorithms is to treat these outputs as features, and optimally compute a weighted average of these features to separate true variants from false positives as the mutationseq method to call somatic mutations from paired tumour-normal sequencing data.
Alternatively, it is also possible to model the joint distribution of these features as a mixture distribution and further compute the posterior probability of a variant to be a true variant.

We performed new analysis with a subset of 12 samples as trainings-set with a combination of weighted calls from now 4 callers: Haplotypecaller, SNPiR, RVboost and MuTect2. We didn’t see any improvements in the error value (the sum of false-positives and false-negatives) even when considering variant allele frequency as a weighted feature. Therefore, we did not change the approach that we used in our workflow. We added an explanation to the discussion: “In addition to the consensus calling of variants by three methods, we tested weighted combinations of the three methods with and without equal dynamic ranges \cite{weight}. We didn't see any improvements in the numbers of true-positive variants, false-negative variants and false-positive variants (see Supplementary Table 5, Supplementary Table 6). Therefore, the approach that uses weighted average features is not implemented in our tool. However, our workflow provides the

possibility of combining calls from any or all callers for further refinement or for adapting to the need of users."

b. An advantage of calling variants from RNA-seq data is the low-cost without sequencing the whole genome or the whole exome.
However, the pipeline in this paper requires normal DNA sequencing data.
The authors should justify why they choose to use normal DNA sequencing data in their pipeline and discuss the influence of these data on the final results.

In the Background, we added a paragraph which justify the need for normal DNA sequencing data: "Additional challenges include the determination of detected mutations either as germline or somatic. In tumor tissues, somatic mutations differ from the germline variations of the patient that are different from the reference genome. To detect somatic sequence variations, it is necessary to compare DNA sequences from normal tissue, such as blood, to DNA or RNA sequences from tumor tissue. If germline sequence variations are not filtered out, it would be difficult to assign detected variations as either somatic or germline. Additionally, it would be improper to assign a variant discovered in the tumor tissue as a somatic mutation when this particular position has no sufficient coverage in germline sequencing."

We also included the following statement in the Discussion:
"It should be noted that current workflow is not completely independent of DNA sequencing since we use germline DNA sequencing to filter out germline variants. However,if the goal is to discover variants in RNA sequencing, VaDiR workflow can be modified to use MuTect2 without germline DNA and to leave out the last filtering step for DP and VAF values in germline DNA. VaDiR may be suitable for tiered studies where VaDiR can be used in the initial step to identify common variants from RNA sequencing datasets, and these candidate mutations can be confirmed by targeted DNA sequencing in a larger cohort to uncover biologically relevant somatic mutations for a specific cancer type. By focusing the initial variant discovery to expressed genes in diseased samples, follow-up validation sequencing efforts can be more targeted to limited regions of interest, thereby lowering the total cost of these genomic studies."

c. When reporting p-values, the statistical test methods and the original data should be provided.

The statistical test method is Two Sample t-test. Original data that were used for all statistical test methods are available at the OSF website using the following urls.
DNA and RNA VAF in sensitive and resistant tumors: https://osf.io/yvc4g/
Number of calls in exonic regions for DNA and Tier1 for RNA in sensitive and resistant tumors: https://osf.io/29p5c/

The following R script can be used to perform t-test:
```
data = read.table("vaf_in_non_cosmic_RNA_and_DNA_between_sensitive_and_ resistant.txt", header=TRUE)
tRNA <- t.test(data$RNA ~ data$Type, var.equal = TRUE)
```

d. Where were the results from the 'additional data' (page 2) presented?

The results are presented in the section “Detection of artificial spiked variants". We clarified this more in the paragraph of the data description: “Additional data used for spiking artificial variants (see section "Detection of artificially spiked variants") were provided by Dr. Andrea Mariani and came from three tumor samples from a patient with serous ovarian carcinoma.”

(2) Presentation:
a. Currently, the paper is a little bit hard to follow, especially for the ANALYSIS section.
Many numbers presented in the main text is not in the tables, and vice versa, some numbers in the tables are not referenced in the main text.
For example, the number 1595677 in Table 1 is never used in the main text.
In addition, the number of DNA positive calls (518 + 9864 = 10382) is different from the number cited in the main text, which is 10099.
These are just some examples, and the authors should go over all the ANALYSIS section to make sure that the results are presented consistently and clearly.
In the current form of the manuscript, it's really difficult to evaluate the results.

We corrected all inconsistencies. We provided all the data in Table formats and also discussed in the main text.

b. For the spiked-in experiments, in the main text, the authors wrote that the experiments were conducted on two tumors, but in Table 2 and Table 3, three tumors were presented.
In addition, why the 'all' rows for both Tier1 and Tier2 variations were the same?

We apologized for the confusing statement. This patient has disseminated ovarian cancer, and we collected multiple tumor samples from different regions/sites. We used three tumor samples collected from two different sites (ovary and omentum) from this patient. We changed the description to make it clear: “To further assess the performance of RNA-based callers, we used BamSurgeon and spiked-in 200 artificial RNA sequence variants at varying variant allele fractions in transcriptomes of three tumor samples from two different tumor sites from one patient.”
In Table 3, the ‘all spiked in variants’ row showed the total of spiked in variants that could be discovered. The 2nd row listed the total of spiked in variants discovered by at least one caller. The 3rd row listed all variants which are not called by VaDiR but are called by at least one caller. Additional rows described the features of missed calls. To clarify the confusing description, we changed the rows so far that the first row show all spiked-in variants, the second row show all variants not called by VaDiR and the third row show all variants not called by VaDiR and are not called by at least one caller.

c. Not sure how the percentages in Table 2 were computed.

The percentages represent the recall rate. Although we spiked in 200 variants, not all spiked in positions are discoverable (because some are located in the regions with low coverage). Also because of some intern filtering processes of the callers not all of the spiked in variants were called. In the process of modifying the parameters of the callers to improve our workflow we

missed to change some resulting numbers in the tables. Those errors are corrected now.

d. To use RNA variants for subclone phylogenetic analysis is interesting but could potentially be challenging given the small number of detected variations in each sample. The author should justify their claim.

We added a citation which explains that targeted sequencing can be used for subclonal phylogenetics: “As shown in [McPherson et al.] subclonal phylogenetics can use limited/targeted sequencing to identify subclones.”

(3) Typos:

RnA - RNA (page 4, line 27)

Corrected.

Reviewer 3:
(1) What is not stated in the abstract is what we see in Figure 1: the VaDiR pipeline requires a normal DNA fastq file, in addition to a tumor RNA fastq file.

My question. Is VaDiR a pipeline for "uncovering mutations from expressed genes using RNA sequencing datasets", or does it require a normal DNA fastq file as suggested by Figure 1? This is even more puzzling as MuTect2 can be used to call mutations from RNAseq data without matched normal DNA or RNA.

VaDiR uses three existing tools to perform variant calls from RNAseq. However, it would be difficult to assign whether discovered variants are somatic or germline without the germline information.
In the Background, we added a paragraph which justify the need for normal DNA sequencing data: “Additional challenges include the determination of detected mutations either as germline or somatic. In tumor tissues, somatic mutations differ from the germline variations of the patient that are different from the reference genome. To detect somatic sequence variations, it is necessary to compare DNA sequences from normal tissue, such as blood, to DNA or RNA sequences from tumor tissue. If germline sequence variations are not filtered out, it would be difficult to assign detected variations as either somatic or germline. Additionally, it would be improper to assign a variant discovered in the tumor tissue as a somatic mutation when this particular position has no sufficient coverage in germline sequencing.”

We agreed with the reviewer that MuTect2 can be used without the germline line data. Therefore, we also included the following statement in the Discussion:
“It should be noted that current workflow is not completely independent of DNA sequencing since we use germline DNA sequencing to filter out germline variants. However,if the goal is to discover variants in RNA sequencing, VaDiR workflow can be modified to use MuTect2 without germline DNA and to leave out the last filtering step for DP and VAF values in germline DNA. VaDiR may be suitable for tiered studies where VaDiR can be used in the initial step to identify common variants from RNA sequencing datasets, and these candidate mutations can be

confirmed by targeted DNA sequencing in a larger cohort to uncover biologically relevant somatic mutations for a specific cancer type. By focusing the initial variant discovery to expressed genes in diseased samples, follow-up validation sequencing efforts can be more targeted to limited regions of interest, thereby lowering the total cost of these genomic studies.”

(2) Intersecting three mutation-calling methods, each with their own specificity is bound to produce a method whose specificity is as large as the largest of the three specificities. So the fact that the Tier 1 combination leads to a higher percentage of calls validated by DNA is no surprise. The question should then be: what loss in sensitivity has been incurred? The authors note that Tier 2: adding back all MuTect2 and SNPiR calls, "leads to higher sensitivity." Again this is as expected, but they complete this observation by commenting that "the precision is still in a moderate range", and do not mention the magnitude of the inevitable decrease in specificity. Each of the three separate calling methods, and the Tier 1 and Tier 2 combinations will have their own specificity and sensitivity. The authors might like to display all of these using their whole exome sequencing data as truth, and let readers decide. It is usually a trade-off between sensitivity and specificity,
though it is not impossible for one method to be best on both criteria.

We have added a table with precision and recall rates for each caller, Tier1, and Tier2.

(3) A natural thing to do when combining three callers is to regard the calls as data, and devise a suitable combination of the three that performs better than all three by combining the strengths of all. It seems possible that such a combination would perform better than the Tier 1 and Tier 2 combinations. Is there some reason why the authors did not do this?

We performed a new analysis with a subset of 12 samples as a training set with a combination of weighted calls using four callers: Haplotypecaller, SNPiR, RVboost and MuTect2. We didn’t see any improvements in the error-value even with an equal dynamic range in the variant allele frequencies. Therefor we will not change our workflow. We added an explanation to the discussion: “In addition to the consensus calling of variants by three methods, we tested weighted combinations of the three methods with and without equal dynamic ranges \cite{weight}. We didn't see any improvements in the numbers of true-positive variants, false-negative variants and false-positive variants (see Supplementary Table 5, Supplementary Table 6). Therefore, the approach that uses weighted average features is not implemented in our tool. However, our workflow provides the possibility of combining calls from any or all callers for further refinement or for adapting to the need of users.”
