# Supplementary material for: VaDiR: an integrated approach to Variant Detection in RNA
Source: Gigascience. 2017 Dec 18;7(2):1–13. doi: 10.1093/gigascience/gix122 (PMC5827345; doi:10.1093/gigascience/gix122)
Supplement: Response_to_Reviewer_Comments_Revision_1.pdf [file gix122_response_to_reviewer_comments_revision_1.pdf]

Author's Response To Reviewer Comments

We thanked the reviewers for insightful suggestion to improve our manuscript.

We provide the following point-by-point response.

Reviewer #2: For a), the authors added some experiment results. However, I still have major concerns about the validity of their analysis and the presentation of the results.

Our response: We included a new figure that demonstrates the approach and the effect of the weighted combination on the performance of variant discovery (Figure 2). The results indicate no improvement gained from the weighted combination in comparison to consensus calling.

1, the authors presented the results in two supplementary tables (without any quantitative comparison), which are very difficult to interpret. Instead, the authors can present the results using figures such as ROC curves for better visualization and interpretation of the results. I suggest the authors quantitatively compare their original approach with the approach based on training classifiers and evaluate the results based on cross-validation analysis (below). Also, the authors can do a clustering analysis of the features (other algorithm outputs).

Our response: To address this comment, we included Figure 2, Precision-Recall curve (Supplementary Figure 2), and Venn's diagram indicating the "evalues" for different combination of callers (Supplementary Figure 1). The Precision-Recall curves show the performance for the best threshold per weighted combination.

2, given that there were not many true mutations, I don't understand why the authors randomly choose a subset of 12 samples for this analysis and completely throw away the rest. Instead, the authors can do a cross-validation analysis on the whole dataset.

We implemented the initial design using two sets (training and test sets). The training set contains 12 samples. However, during the training to determine the best weighted combination, we discovered that weighted approach shows no improvement over the consensus calling. Therefore, we never used the test set.

3, the authors did not present their detailed analysis. If there was not enough space, the authors can at least briefly talked about their analysis in the main text, and left the details in supplementary materials.

We revised the manuscript to briefly describe the methods and analysis of weighted combination of callers in the main text. We provided the details of the approach in the supplement.
