# Supplementary material for: VaDiR: an integrated approach to Variant Detection in RNA
Source: Gigascience. 2017 Dec 18;7(2):1–13. doi: 10.1093/gigascience/gix122 (PMC5827345; doi:10.1093/gigascience/gix122)
Supplement: Response_to_Reviewer_Comments_Revision_2.pdf [file gix122_response_to_reviewer_comments_revision_2.pdf]

Author's Response To Reviewer Comments

Dear Dr. Zauner,

Thank you for your suggestions on the revision of our manuscript, titled “VaDiR: an integrated approach to Variant Detection in RNA”

Per your suggestion, we revised the manuscript to include the following:

A reference to the GigaDB entry with DOI.
[43] Neums L, Suenaga S, Beyerlein P, Koestler D, Anders S, Mariani A, Chien J. Supporting software for "VaDiR: an integrated approach to Variant Detection in RNA". 2017. http://dx.doi.org/10.5524/100360

We included the following statement in the “Availability of Supporting Data”:
"Supporting data and an archival copy of the code are also available via the GigaScience repository GigaDB [43].
For testing purposes we utilised data kindly provided by Dr. Andrea Mariani of Mayo Clinic, Rochester, Minnesota. Due to ethical constraints these data cannot be shared publicly, but if researchers would like to request access to these data please contact Dr. Andrea Mariani (mariani.andrea@mayo.edu) with a short description of why you require access and how you would use the data."

We updated the manuscript to include the project URL and RRID.

We also included information about third party code in supplemental table 3 (including citation and licence info).

Best regards,
Jeremy Chien
