# Supplementary material for: VaDiR: an integrated approach to Variant Detection in RNA
Source: Gigascience. 2017 Dec 18;7(2):1–13. doi: 10.1093/gigascience/gix122 (PMC5827345; doi:10.1093/gigascience/gix122)
Supplement: Reviewer_1_Report_(Original_Submission) -- Kou-Chen Chou — 15 Jan 2017 Reviewed [file gix122_reviewer_1_report_(original_submission).pdf]

Reviewer Report

Title: VaDiR: an integrated approach to Variant Detection in RNA

Version: Original Submission **Date:** 1/15/2017

Reviewer name: Kou-Chen Chou

Reviewer Comments to Author:

In this paper the authors addressed an important problem. In view of this, it holds high potential for publication. But to meet the increasingly quality standard of GigaScience, a careful revision is absolutely needed according to the following points.

(1) To make the structure of this paper logically more clear and practically more useful, the authors should in the end of the Introduction (or right before the beginning of describing their own method) add a prelude, such as: "As demonstrated by a series of recent publications [1-7] in compliance with the 5-step rule [8], to establish a really useful sequence-based statistical predictor for a biological system, we should follow the following five guidelines: (a) construct or select a valid benchmark dataset to train and test the predictor; (b) formulate the biological sequence samples with an effective mathematical expression that can truly reflect their intrinsic correlation with the target to be predicted; (c) introduce or develop a powerful algorithm (or engine) to operate the prediction; (d) properly perform cross-validation tests to objectively evaluate the anticipated accuracy of the predictor; (e) establish a user-friendly web-server for the predictor that is accessible to the public. Below, we are to describe how to deal with these steps one-by- one." With such a prelude, the outline of this paper and its aim would be crystal clear.

(2) Recently, some very powerful bioinformatics tools for analyzing DNA/RNA sequences have been developed [9-14]. The authors should explicitly mention these powerful tools to provide the readership with an updated background and rapid development in this area. The authors should also mentioned a recent paper [15] in the context relevant to the NGS(next-generation sequencing).

(3) It is pity that the authors did not provide a web-server for their new method of VaDiR presented in this paper. To attract the readership to their future work and to the GigaScience journal as well, the authors should add a discussion in the end of their paper, such as: "As demonstrated in a series of recent publications (see, e.g., [2,3,5,7,16-22]) in developing new prediction or detection methods, user-friendly and publicly accessible web-servers will significantly enhance their impacts [23], we shall make efforts in our future work to provide a web-server for the detection method reported in this paper."

This Reviewer believes that the current paper will be significantly improved in quality and substantially enriched in contents, so as to meet the quality standard of GigaScience if the authors can satisfactorily incorporate the aforementioned suggestions into the paper.

REFERENCES

[1] Jia, J.; Liu, Z.; Xiao, X.; Liu, B. pSuc-Lys: Predict lysine succinylation sites in proteins with PseAAC and ensemble random forest approach. Journal of Theoretical Biology, 2016, 394, 223-230.
[2] Jia, J.; Liu, Z.; Xiao, X. iCar-PseCp: identify carbonylation sites in proteins by Monto Carlo sampling and incorporating sequence coupled effects into general PseAAC. Oncotarget, 2016, 7, 34558-34570.
[3] Jia, J.; Zhang, L.; Liu, Z. pSumo-CD: Predicting sumoylation sites in proteins with covariance discriminant algorithm by incorporating sequence-coupled effects into general PseAAC. Bioinformatics, 2016, 32, 3133-3141.
[4] Qiu, W.R.; Sun, B.Q.; Xu, D. iPhos-PseEvo: Identifying human phosphorylated proteins by incorporating evolutionary information into general PseAAC via grey system theory. Molecular Informatics, 2016, doi:10.1002/minf.201600010.
[5] Qiu, W.R.; Sun, B.Q.; Xiao, X.; Xu, Z.C. iHyd-PseCp: Identify hydroxyproline and hydroxylysine in proteins by incorporating sequence-coupled effects into general PseAAC. Oncotarget, 2016, 7, 44310-44321.
[6] Qiu, W.R.; Sun, B.Q.; Xu, Z.C.. iPTM-mLys: identifying multiple lysine PTM sites and their different types. Bioinformatics, 2016, 32, 3116-3123.
[7] Qiu, W.R.; Xiao, X.; Xu, Z.H.. iPhos-PseEn: identifying phosphorylation sites in proteins by fusing different pseudo components into an ensemble classifier. Oncotarget, 2016, 7, 51270-51283.
[8] Chou, K.C. Some remarks on protein attribute prediction and pseudo amino acid composition (50th Anniversary Year Review). Journal of Theoretical Biology, 2011, 273, 236-247.
[9] Chen, W.; Lei, T.Y.; Jin, D.C.; Lin, H. PseKNC: a flexible web-server for generating pseudo K-tuple nucleotide composition. Analytical Biochemistry, 2014, 456, 53-60.
[10] Guo, S.H.; Deng, E.Z.; Xu, L.Q.; Ding, H.. iNuc-PseKNC: a sequence-based predictor for predicting nucleosome positioning in genomes with pseudo k-tuple nucleotide composition. Bioinformatics, 2014, 30, 1522-1529.
[11] Chen, W.; Lin, H. Pseudo nucleotide composition or PseKNC: an effective formulation for analyzing genomic sequences. Mol Biosyst, 2015, 11, 2620-2634.
[12] Liu, B.; Liu, F.; Fang, L.; Wang, X. repDNA: a Python package to generate various modes of feature vectors for DNA sequences by incorporating user-defined physicochemical properties and sequence-order effects. Bioinformatics, 2015, 31, 1307-1309.
[13] Liu, B.; Liu, F.; Fang, L. repRNA: a web server for generating various feature vectors of RNA sequences. Molecular Genetics and Genomics, 2016, 291, 473-481.
[14] Liu, B.; Liu, F.; Wang, X.; Chen, J. Pse-in-One: a web server for generating various modes of pseudo components of DNA, RNA, and protein sequences Nucleic Acids Research, 2015, 43, W65-W71.
[15] Cai, L.; Yuan, W.; Zhang, Z. In-depth comparison of somatic point mutation callers based on different tumor next-generation sequencing depth data Scientific Reports, 2016, 6, 36540.
[16] Chen, W.; Ding, H.; Feng, P. iACP: a sequence-based tool for identifying anticancer peptides. Oncotarget, 2016, 7, 16895-16909.
[17] Chen, W.; Feng, P.; Yang, H.; Ding, H. iRNA-AI: identifying the adenosine to inosine editing sites in RNA sequences. Oncotarget, 2016, doi:10.18632/oncotarget.13758.
[18] Liu, B.; Wu, H.; Zhang, D.. Pse-Analysis: a python package for DNA/RNA and protein/peptide

sequence analysis based on pseudo components and kernel methods. Oncotarget, 2017, doi: 10.18632/oncotarget.14524.
[19] Xiao, X.; Ye, H.X.; Liu, Z.; Jia, J.H. iROS-gPseKNC: predicting replication origin sites in DNA by incorporating dinucleotide position-specific propensity into general pseudo nucleotide composition. Oncotarget, 2016, 7, 34180-34189.
[20] Zhang, C.J.; Tang, H.; Li, W.C.; Lin, H. iOri-Human: identify human origin of replication by incorporating dinucleotide physicochemical properties into pseudo nucleotide composition. Oncotarget, 2016, 7, 69783-69793.
[21] Chen, W.; Tang, H.; Ye, J. iRNA-PseU: Identifying RNA pseudouridine sites Molecular Therapy - Nucleic Acids, 2016, 5, e332.
[22] Liu, Z.; Xiao, X.; Yu, D.J.; Jia, J. pRNAm-PC: Predicting N-methyladenosine sites in RNA sequences via physical-chemical properties. Analytical Biochemistry, 2016, 497, 60-67.
[23] Chou, K.C. Impacts of bioinformatics to medicinal chemistry. Medicinal Chemistry, 2015, 11, 218-234.

Methods

Are the methods appropriate to the aims of the study, are they well described, and are necessary controls included? Yes

Conclusions

Are the conclusions adequately supported by the data shown? Yes

Reporting Standards

Does the manuscript adhere to the journal’s guidelines on minimum standards of reporting? YesChoose an item.

Statistics

Are you able to assess all statistics in the manuscript, including the appropriateness of statistical tests used? No, and I do not feel adequately qualified to assess the statistics.

Quality of Written English

Please indicate the quality of language in the manuscript: Acceptable

Declaration of Competing Interests

Please complete a declaration of competing interests, considering the following questions:

- Have you in the past five years received reimbursements, fees, funding, or salary from an organisation that may in any way gain or lose financially from the publication of this manuscript, either now or in the future?
- Do you hold any stocks or shares in an organisation that may in any way gain or lose financially from the publication of this manuscript, either now or in the future?
- Do you hold or are you currently applying for any patents relating to the content of the manuscript?
- Have you received reimbursements, fees, funding, or salary from an organization that holds or has applied for patents relating to the content of the manuscript?
- Do you have any other financial competing interests?
- Do you have any non-financial competing interests in relation to this paper?

If you can answer no to all of the above, write 'I declare that I have no competing interests' below. If your reply is yes to any, please give details below.

I declare that I have no competing interests

I agree to the open peer review policy of the journal. I understand that my name will be included on my report to the authors and, if the manuscript is accepted for publication, my named report including any attachments I upload will be posted on the website along with the authors' responses. I agree for my report to be made available under an Open Access Creative Commons CC-BY license (http://creativecommons.org/licenses/by/4.0/). I understand that any comments which I do not wish to be included in my named report can be included as confidential comments to the editors, which will not be published.

I agree to the open peer review policy of the journal

To further support our reviewers, we have joined with Publons, where you can gain additional credit to further highlight your hard work (see: https://publons.com/journal/530/gigascience). On publication of this paper, your review will be automatically added to Publons, you can then choose whether or not to claim your Publons credit. I understand this statement.

Yes
