# Supplementary material for: VaDiR: an integrated approach to Variant Detection in RNA
Source: Gigascience. 2017 Dec 18;7(2):1–13. doi: 10.1093/gigascience/gix122 (PMC5827345; doi:10.1093/gigascience/gix122)
Supplement: Reviewer_2_Report_(Original_Submission) -- Jiarui Ding — 26 Jan 2017 Reviewed [file gix122_reviewer_2_report_(original_submission).pdf]

Reviewer Report

Title: VaDiR: an integrated approach to Variant Detection in RNA

Version: Original Submission **Date:** 1/26/2017

Reviewer name: Jiarui Ding

Reviewer Comments to Author:

The paper presents a pipeline to call variants from RNA-seq data by combining the results from three software tools.
The tool could be of interests as single-cell RNA sequencing technology becomes mature, and this method could be extended to call variants from scRNA-seq data.
Here I have some major concerns with the method itself and how the results are presented, which need to be addressed.

Major comments
Methods:

a) The authors simply computed the overlap of the variant calls from three methods, SNPiR, RVBoost, and MuTect2.
Therefore, the census calls could be very sensitive to the results of the three algorithms.
The authors also noticed that some variations with high expression and high variant allele frequencies were either not called by any of the three methods or were filtered out by at least one of the three methods.
A more principled way to combine the outputs of various algorithms is to treat these outputs as features, and optimally compute a weighted average of these features to separate true variants from false positives as the mutationseq method to call somatic mutations from paired tumour-normal sequencing data.
Alternatively, it is also possible to model the joint distribution of these features as a mixture distribution and further compute the posterior probability of a variant to be a true variant.

b) An advantage of calling variants from RNA-seq data is the low-cost without sequencing the whole genome or the whole exome.
However, the pipeline in this paper requires normal DNA sequencing data.
The authors should justify why they choose to use normal DNA sequencing data in their pipeline and discuss the influence of these data on the final results.

c) When reporting p-values, the statistical test methods and the original data should be provided.

d) Where were the results from the 'additional data' (page 2) presented?

Presentation:

a) Currently, the paper is a little bit hard to follow, especially for the ANALYSIS section.
Many numbers presented in the main text is not in the tables, and vice versa, some numbers in the tables are not referenced in the main text.
For example, the number 1595677 in Table 1 is never used in the main text.
In addition, the number of DNA positive calls (518 + 9864 = 10382) is different from the number cited in the main text, which is 10099.
These are just some examples, and the authors should go over all the ANALYSIS section to make sure that the results are presented consistently and clearly.
In the current form of the manuscript, it's really difficult to evaluate the results.
b) For the spiked-in experiments, in the main text, the authors wrote that the experiments were conducted on two tumors, but in Table 2 and Table 3, three tumors were presented.
In addition, why the 'all' rows for both Tier1 and Tier2 variations were the same?
c) Not sure how the percentages in Table 2 were computed.
d) To use RNA variants for subclone phylogenetic analysis is interesting but could potentially be challenging given the small number of detected variations in each sample. The author should justify their claim.

Typos:

RnA - RNA (page 4, line 27)

Methods

Are the methods appropriate to the aims of the study, are they well described, and are necessary controls included? Yes

Conclusions

Are the conclusions adequately supported by the data shown? Yes

Reporting Standards

Does the manuscript adhere to the journal's guidelines on minimum standards of reporting? YesChoose an item.

Statistics

Are you able to assess all statistics in the manuscript, including the appropriateness of statistical tests used? Yes, and I have assessed the statistics in my report.

Quality of Written English

Please indicate the quality of language in the manuscript: Needs some language corrections before being published

Declaration of Competing Interests

Please complete a declaration of competing interests, considering the following questions:

- Have you in the past five years received reimbursements, fees, funding, or salary from an organisation that may in any way gain or lose financially from the publication of this manuscript, either now or in the future?
- Do you hold any stocks or shares in an organisation that may in any way gain or lose financially from the publication of this manuscript, either now or in the future?
- Do you hold or are you currently applying for any patents relating to the content of the manuscript?
- Have you received reimbursements, fees, funding, or salary from an organization that holds or has applied for patents relating to the content of the manuscript?
- Do you have any other financial competing interests?
- Do you have any non-financial competing interests in relation to this paper?

If you can answer no to all of the above, write 'I declare that I have no competing interests' below. If your reply is yes to any, please give details below.

I declare that I have no competing interests

I agree to the open peer review policy of the journal. I understand that my name will be included on my report to the authors and, if the manuscript is accepted for publication, my named report including any attachments I upload will be posted on the website along with the authors' responses. I agree for my report to be made available under an Open Access Creative Commons CC-BY license (http://creativecommons.org/licenses/by/4.0/). I understand that any comments which I do not wish to be included in my named report can be included as confidential comments to the editors, which will not be published.

I agree to the open peer review policy of the journal

To further support our reviewers, we have joined with Publons, where you can gain additional credit to further highlight your hard work (see: https://publons.com/journal/530/gigascience). On publication of this paper, your review will be automatically added to Publons, you can then choose whether or not to claim your Publons credit. I understand this statement.

Yes
