# Supplementary material for: VaDiR: an integrated approach to Variant Detection in RNA
Source: Gigascience. 2017 Dec 18;7(2):1–13. doi: 10.1093/gigascience/gix122 (PMC5827345; doi:10.1093/gigascience/gix122)
Supplement: Reviewer_2_Report_(Revision_1) -- Jiarui Ding — 19 Jun 2017 Reviewed [file gix122_reviewer_2_report_(revision_1).pdf]

Reviewer Report

Title: VaDiR: an integrated approach to Variant Detection in RNA

Version: Revision 1 **Date:** 6/19/2017

Reviewer name: Jiarui Ding

Reviewer Comments to Author:

For a), the authors added some experiment results. However, I still have major concerns about the validity of their analysis and the presentation of the results.1, the authors presented the results in two supplementary tables (without any quantitative comparison), which are very difficult to interpret. Instead, the authors can present the results using figures such as ROC curves for better visualization and interpretation of the results. I suggest the authors quantitatively compare their original approach with the approach based on training classifiers and evaluate the results based on cross-validation analysis (below). Also, the authors can do a clustering analysis of the features (other algorithm outputs).2, given that there were not many true mutations, I don't understand why the authors randomly choose a subset of 12 samples for this analysis and completely throw away the rest. Instead, the authors can do a cross-validation analysis on the whole dataset.
3, the authors did not present their detailed analysis. If there was not enough space, the authors can at least briefly talked about their analysis in the main text, and left the details in supplementary materials.

Level of Interest

Please indicate how interesting you found the manuscript: An article of importance in its field

Quality of Written English

Please indicate the quality of language in the manuscript: Acceptable

Declaration of Competing Interests

Please complete a declaration of competing interests, considering the following questions:

- Have you in the past five years received reimbursements, fees, funding, or salary from an organisation that may in any way gain or lose financially from the publication of this manuscript, either now or in the future?
- Do you hold any stocks or shares in an organisation that may in any way gain or lose financially from the publication of this manuscript, either now or in the future?
- Do you hold or are you currently applying for any patents relating to the content of the manuscript?
- Have you received reimbursements, fees, funding, or salary from an organization that holds or has applied for patents relating to the content of the manuscript?

- Do you have any other financial competing interests?
- Do you have any non-financial competing interests in relation to this paper?

If you can answer no to all of the above, write 'I declare that I have no competing interests' below. If your reply is yes to any, please give details below.

I declare that I have no competing interests

I agree to the open peer review policy of the journal. I understand that my name will be included on my report to the authors and, if the manuscript is accepted for publication, my named report including any attachments I upload will be posted on the website along with the authors' responses. I agree for my report to be made available under an Open Access Creative Commons CC-BY license (http://creativecommons.org/licenses/by/4.0/). I understand that any comments which I do not wish to be included in my named report can be included as confidential comments to the editors, which will not be published.

I agree to the open peer review policy of the journal
