# Supplementary material for: VaDiR: an integrated approach to Variant Detection in RNA
Source: Gigascience. 2017 Dec 18;7(2):1–13. doi: 10.1093/gigascience/gix122 (PMC5827345; doi:10.1093/gigascience/gix122)
Supplement: Reviewer_3_Report_(Original_Submission) -- Terry Speed — 03 Mar 2017 Reviewed [file gix122_reviewer_3_report_(original_submission).pdf]

Reviewer Report

Title: VaDiR: an integrated approach to Variant Detection in RNA

Version: Original Submission **Date:** 3/3/2017

Reviewer name: Terry Speed

Reviewer Comments to Author:

1. The abstract of the paper says

:We investigated the feasibility of uncovering mutations from expressed genes using RNA sequencing datasets with a method called "VaDiR: Variant Detection in RNA" that integrate three variant callers, namely: SNPiR, RVBoost and MuTect2."

What is not stated in the abstract is what we see in Figure 1: the VaDiR pipeline requires a normal DNA fastq file, in addition to a tumor RNA fastq file.

My question. Is VaDiR a pipeline for "uncovering mutations from expressed genes using RNA sequencing datasets", or does it require a normal DNA fastq file as suggested by Figure 1? This is even more puzzling as MuTect2 can be used to call mutations from RNAseq data without matched normal DNA or RNA.

2. Intersecting three mutation-calling methods, each with their own specificity is bound to produce a method whose specificity is as large as the largest of the three specificities. So the fact that the Tier 1 combination leads to a higher percentage of calls validated by DNA is no surprise. The question should then be: what loss in sensitivity has been incurred? The authors note that Tier 2: adding back all MuTect2 and SNPiR calls, "leads to higher sensitivity." Again this is as expected, but they complete this observation by commenting that "the precision is still in a moderate range", and do not mention the magnitude of the inevitable decrease in specificity. Each of the three separate calling methods, and the Tier 1 and Tier 2 combinations will have their own specificity and sensitivity. The authors might like to display all of these using their whole exome sequencing data as truth, and let readers decide. It is usually a trade-off between sensitivity and specificity, though it is not impossible for one method to be best on both criteria.

3. A natural thing to do when combining three callers is to regard the calls as data, and devise a suitable combination of the three that performs better than all three by combining the strengths of all. It seems possible that such a combination would perform better than the Tier 1 and Tier 2 combinations. Is there some reason why the authors did not do this?

Methods

Are the methods appropriate to the aims of the study, are they well described, and are necessary controls included? No

Conclusions

Are the conclusions adequately supported by the data shown? Yes

Reporting Standards

Does the manuscript adhere to the journal's guidelines on minimum standards of reporting? YesChoose an item.

Statistics

Are you able to assess all statistics in the manuscript, including the appropriateness of statistical tests used? There are no statistics in the manuscript.

Quality of Written English

Please indicate the quality of language in the manuscript: Acceptable

Declaration of Competing Interests

Please complete a declaration of competing interests, considering the following questions:

- Have you in the past five years received reimbursements, fees, funding, or salary from an organisation that may in any way gain or lose financially from the publication of this manuscript, either now or in the future?
- Do you hold any stocks or shares in an organisation that may in any way gain or lose financially from the publication of this manuscript, either now or in the future?
- Do you hold or are you currently applying for any patents relating to the content of the manuscript?
- Have you received reimbursements, fees, funding, or salary from an organization that holds or has applied for patents relating to the content of the manuscript?
- Do you have any other financial competing interests?
- Do you have any non-financial competing interests in relation to this paper?

If you can answer no to all of the above, write 'I declare that I have no competing interests' below. If your reply is yes to any, please give details below.

I declare that I have no competing interests

I agree to the open peer review policy of the journal. I understand that my name will be included on my report to the authors and, if the manuscript is accepted for publication, my named report including any attachments I upload will be posted on the website along with the authors' responses. I agree for my report to be made available under an Open Access Creative Commons CC-BY license

(http://creativecommons.org/licenses/by/4.0/). I understand that any comments which I do not wish to be included in my named report can be included as confidential comments to the editors, which will not be published.

I agree to the open peer review policy of the journal

To further support our reviewers, we have joined with Publons, where you can gain additional credit to further highlight your hard work (see: https://publons.com/journal/530/gigascience). On publication of this paper, your review will be automatically added to Publons, you can then choose whether or not to claim your Publons credit. I understand this statement.

Yes
